# Mito-nuclear discordance reveals introgressive hybridization following vicariance and secondary contact in Iberian scorpions (Buthidae: *Buthus*)

**DOI:** 10.1186/s12862-025-02445-0

**Published:** 2025-10-23

**Authors:** Javier Blasco-Aróstegui, Yuri Simone, Octávio S. Paulo, Lorenzo Prendini

**Affiliations:** 1 Centre for Ecology, Evolution and Environmental Changes & CHANGE - Global Change and Sustainability Institute, Departamento de Biologia Animal, Faculdade de Ciências, Universidade de Lisboa, Campo Grande 016, Lisbon, 1749-016 Portugal; 2 Arachnology Lab and Scorpion Systematics Research Group, Division of Invertebrate Zoology, American Museum of Natural History, Central Park West at 79th Street, New York, NY 10024-5192 USA; 3 Laboratory of Functional Morphology, Department of Biology, University of Antwerp, Universiteitsplein 1, 2610, Wilrijk Belgium

**Keywords:** Messinian salinity crisis, Pleistocene climatic oscillations, genetic admixture, intermediate character combinations, environmental niche modeling

## Abstract

Mito-nuclear (or cytonuclear) discordance, evolutionary incongruence between the mitochondrial and nuclear genomes, is a widespread but underappreciated phenomenon that may obscure signals of introgression and hybridization important for understanding evolutionary trajectories and species boundaries. The present study explored the roles of secondary contact and introgressive hybridization in the diversification of *Buthus* Leach, 1815 scorpions in the Iberian Peninsula, a hotspot for mito-nuclear discordance, in which complex topography and glacial history facilitated repeated cycles of isolation and secondary contact. Patterns of mito-nuclear discordance were predicted to be consistent with overlapping distributions, similar ecological niches, and intermediate phenotypes. By integrating genomic, morphological, and ecological data, the intricate evolutionary history of *Buthus*, shaped by vicariance, reticulation and ecological opportunity, was revealed. The results underscore the role of introgressive hybridization in shaping patterns of biodiversity and the need to consider mito-nuclear discordance in species delimitation.

**Supplementary Information:**

The online version contains supplementary material available at 10.1186/s12862-025-02445-0.

## Introduction

Incongruence between the evolutionary histories recovered by a species’ nuclear (nuDNA) and mitochondrial (mtDNA) genomes—known as cytonuclear or mito-nuclear discordance—is often overlooked in molecular studies [1–3]. The sole reliance on mtDNA or on datasets of concatenated nuDNA and mtDNA sequences may obscure detection of introgression, important for understanding evolutionary and phylogeographic patterns [4–6]. When introgression and admixture are concealed, the ability to accurately reconstruct population dynamics and ancestral relationships is reduced [7].

Numerous studies documented cases of mito-nuclear discordance but few explored their taxonomic implications [8–11]. Species delimitation becomes challenging when incongruence, arising from differences in the resolution of markers, is compounded by hybridization events [12, 13] that produce reticulate patterns of lineage formation and conflicting character distributions [4, 8, 11, 14]. Taxonomic decisions are further complicated when reticulation manifests phenotypically as intermediate combinations of morphological characters or transgressive traits relative to parental lineages [15–18].

Europe, and particularly the Iberian Peninsula, appears to be a hotspot for mito-nuclear discordance [18–24]. Temporary ecological corridors and land bridges, shaped by sea-level fluctuations and a rugged landscape with many glacial refugia, promoted repeated cycles of population expansion and contraction [19, 22, 25–28]. These dynamic processes facilitated secondary contact between incipient species when periods of allopatry were too brief to induce reproductive isolation. As a result, incomplete lineage sorting (ILS) became commonplace, contributing to complex genetic patterns in the region. Iberian beetles (*Mesocarabus* Thomson, 1875), fire salamanders (*Salamandra* Garsault, 1764), and toads (*Alytes* Wagler, 1830) provide examples in which introgression of mtDNA haplotypes, e.g., mitochondrial capture by nuDNA lineages, was commonly observed, with patterns of diversification following similar trajectories, e.g., south–north dispersal [8, 21, 29–31].

**Fig. 1:**
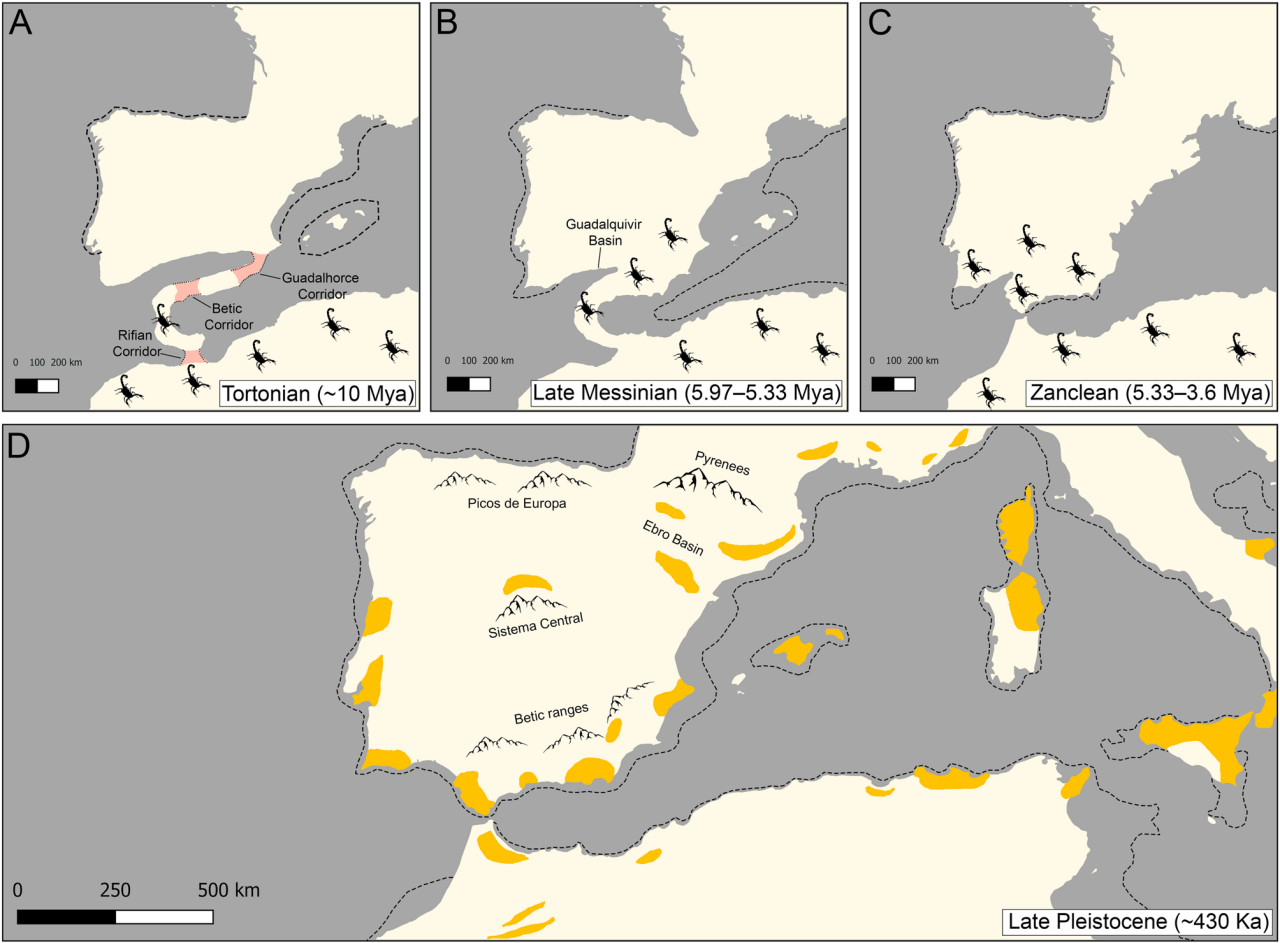
Hypothetical colonization routes and speciation patterns in European species of *Buthus* Leach, 1815, with major geological events potentially influencing diversification and secondary contact (based on data extracted from Paulo et al., 2008, Klesser et al., 2021 and Costa et al., 2022). **A** Tortonian, illustrating three major Eurafrican ecological corridors (Betic, Rifian and Guadalhorce) that facilitated faunal exchanges between North Africa and the Iberian Peninsula. **B** Late Messinian, showing extensive land bridge that reconnected the Iberian Peninsula and North Africa during the Messinian Salinity Crisis. **C** Zanclean, when Atlantic waters refilled the Mediterranean Basin, disrupting the land bridge and isolating the Iberian Peninsula from North Africa. **D** Late Pleistocene, putative glacial refugia for flora and fauna (orange polygons represent mountain ranges), adapted from Médail and Diadema (2009) and Gómez and Lunt (2007). Dashed lines represent extent of exposed continental platform during each period

Scorpions of the genus *Buthus* Leach, 1815 colonized the Iberian Peninsula from North Africa via one or more land bridges, e.g., the Rifian, Betic and Guadalhorce corridors, approximately 10–6 million years ago (Mya) [26, 28, 32–35] (Fig. 1). Gene flow with North African populations was tentatively interrupted between 5.42 and 3.38 Mya [35], encompassing the estimated date for the reopening of the Strait of Gibraltar during the Messinian Salinitiy Crisis (MSC) around 5.33 Mya [26, 28, 36, 37]. Early studies on *Buthus* suggested that these scorpions experienced introgressive hybridization events [38–40]. Orographic barriers, ILS, and the absence of nuDNA data further challenged resolution of their phylogenetic history [33–35, 41, 42]. *Buthus* scorpions possess highly conserved intromittent organs, i.e., the male hemispermatophores [43], sex-biased dispersal, with sedentary females confined to burrows while errant males traverse larger areas in search of mates [44, 45], and mitofusion, a unique form of mitochondrial segregation during early spermatogenesis in which mitochondria fuse into a ring within the primary spermatocytes, thus far reported only in buthids across the animal kingdom [40]. These attributes create a scenario for preferential introgression and recombination of mtDNA [40, 46, 47], promoting homogenization of nuDNA while accentuating mtDNA regionalization, which has been misconstrued as taxonomic diversity. Long considered a single species, the Iberian species of *Buthus* experienced a dramatic inflation in diversity, to twenty species, during the past two decades [33, 42, 48–61]. The brief, uninformative diagnoses provided in these studies, along with the absence of identification keys and rigorous analyses of the morphological and molecular variation underscored the need to reassess the validity of the putative new species and the criteria used for their delimitation [43].

The present study investigated the roles of secondary contact and introgressive hybridization, as well as putative ILS, in the diversification of *Buthus* on the Iberian Peninsula. High levels of genetic divergence were predicted in topographically complex areas (e.g., mountain ranges) which represent refugia and provide barriers to dispersal and gene flow. Lower levels of divergence, i.e., more homogeneity, and mito-nuclear discordance were predicted in less topographically complex areas (e.g., valleys and plains), which represent ancient and/or extant ecological corridors and zones of potential secondary contact. Patterns of mito-nuclear discordance were predicted to be consistent with overlapping distributions, similar ecological niches, and intermediate phenotypes, comprising combinations of morphological characters arising from admixture between distinct lineages, as in other taxa [8, 15–18].

By integrating genomic, morphological, and ecological data, the intricate evolutionary history of *Buthus*, shaped by vicariance, reticulation and ecological opportunity, was revealed. The results underscore the role of introgressive hybridization in shaping patterns of biodiversity and the need to consider mito-nuclear discordance in species delimitation.

## Materials and methods

### Taxon sampling

Targeted fieldwork was conducted across the known distribution of *Buthus* in Europe (France, Portugal, and Spain), including the type localities of each putative species and additional records from the literature [34, 48–52, 55–62] and iNaturalist (https://www.inaturalist.org; Table 1, S2). Freshly collected material, including topotypes [43], preserved in 70% ethanol for morphological examination, is deposited in the collections of the American Museum of Natural History (AMNH), New York. Tissue samples, preserved in 95% ethanol, are deposited in the Ambrose Monell Cryocollection for Molecular and Microbial Research (AMCC) at the AMNH.

**Table 1:** Putative European species of *Buthus* leach, 1815, including type localities, known countries of occurrence, topotypes deposited in the Ambrose Monell cryocollection (AMCC) at the American Museum of Natural History, New York, and nuclear (nuDNA) and mitochondrial (mtDNA) lineages associated with each

Species	Type locality	Countries	AMCC	nuDNA	mtDNA
*Buthus ajax* (C.L. Koch, 1839)	Toledo: Porzuna	Spain	LP 19493	A	L
*Buthus alacanti* Teruel & Turiel, 2020	Alicante: Sant Vicent del Raspeig	Spain	LP 19744	F	F
*Buthus baeticus* Teruel & Turiel, 2020	Sevilla: Sierra de Esparteros	Spain	LP 19801	A	B
*Buthus balmensis* Ythier & Laborieux, 2022	Var: Massif Sainte-Baume	France	LP 19715	H	H
*Buthus castellano* Teruel & Turiel, 2022	Valladolid: Herrera de Duero	Spain	LP 19498	G	L
*Buthus delafuentei* Teruel & Turiel, 2020	Huelva: Pinar del Tableado	Portugal, Spain	LP 19816	A	A
*Buthus elongatus* Rossi, 2012	Málaga: Sierra de las Nieves	Spain	LP 19791	C	C
*Buthus gabani* Ythier, 2021	Faro: Cabo São Vicente	Portugal	LP 18063	A	I
*Buthus garcialorcai* Teruel & Turiel, 2020	Granada: Sierra de Huétor	Spain	LP 19780	D	D
*Buthus gonzalezdelavegai*, González-Moliné & Armas, 2024	Huelva: Matalascañas	Spain	LP 20690	A	A
*Buthus halius* (C.L. Koch, 1839)	Vila Real: Tua riverbank	Portugal	LP 18040	G	G
*Buthus iaspis* Teruel & Turiel, 2022	Almería: Las Amoladeras	Spain	LP 19760	E	J
*Buthus ibericus* Lourenço & Vachon, 2004	Cádiz: Dehesa Picado	Spain	LP 19795	B	B
*Buthus lusitanus* Lourenço, 2021	Guarda: Vale Glaciar do Zêzere	Portugal	LP 19515	A	L
*Buthus manchego* Teruel & Turiel, 2020	Ciudad Real: Peñarroya	Spain	LP 19524	F	F
*Buthus montanus* Lourenço & Vachon, 2004	Granada: Puerto de la Ragua	Spain	LP 19769	E	E
*Buthus occitanus* (Amoreux, 1789)	Gard: Souvignargues	France, Spain	LP 19717	H	H
*Buthus pedrosousai* Teruel & Turiel, 2021	Cuenca: Ribatajadilla	Spain	LP 19533	G	F
*Buthus pyrenaeus* Ythier, 2021	Pyrénées-Orientales: Cerbère	France, Spain	LP 19725	H	H
*Buthus serrano* Teruel & Turiel, 2020	Granada: Orce	Spain	LP 19767	F	F

The ingroup comprised topotypes and additional representatives of all putative European species, 51 terminals in total (Table 1, S2). All except three putative species were represented by two or more terminals. Three African species were included as outgroups. Two of the outgroup species represented the major North African *Buthus* clades found to be most closely related to the European clade in previous analyses [33–35, 38, 39, 41, 42, 53]: *Buthus maroccanus* Birula, 1903, part of the Moroccan *occitanus* complex, represented the Atlas clade; and *Buthus tunetanus* (Herbst, 1800), part of the *tunetanus* complex, represented the Tell-Atlas clade. The phylogeny was rooted on *Buthus elizabethae* Lourenço, 2005, a West African species genetically divergent from the clades of northwestern Africa and Europe.

### Morphological datasets

Morphology was studied using a Nikon SMZ1500 stereoscope and measurements (mm), recorded with Mitutoyo^®^ digital calipers. Twenty-four qualitative characters and twelve quantitative characters, mostly pertaining to the adult stage (especially the adult male), were scored in a morphological matrix that captured variation among the European ingroup species and three African outgroup species of *Buthus* [43]. The matrix was constructed using Mesquite 3.81 [63].

A second dataset, comprising 25 morphometrics, calculated from measurements of 189 adult specimens representing all putative European species of *Buthus* and the three African outgroup species [43], was prepared for multivariate analyses, conducted separately for males and females. Twenty-four morphometric ratios, along with total body length divided by 100 to standardize values between 0 and 1 [64], were used as predictors in Linear Discriminant Analyses (LDA). All variables were log10-transformed, and assessed for multidimensional linearity, homoscedasticity and collinearity.

### DNA extraction, PCR and sequencing

DNA extraction, Polymerase Chain Reaction (PCR) and sequencing were conducted at the AMNH Institute for Comparative Genomics. DNA was extracted using a Qiagen DNAeasy Blood & Tissue kit (Germantown, MD). Leg muscle tissue was dissected and digested with Proteinase K for 24–72 h in an incubator, and eluted in 200 µl of AE buffer.

Three nuclear loci, the small subunit ribosomal RNA or 18S rRNA (18S), the Internal Transcribed Spacer (ITSII) and the D3 region of the large subunit ribosomal RNA or 28S rRNA (28S), and three mitochondrial loci, the small subunit ribosomal RNA or 12S rRNA (12S), the large subunit ribosomal RNA or 16S rRNA (16S), and the Cytochrome *c* Oxidase subunit I (COI), were amplified using standard primers (Table S1). This combination of loci was selected due to its high information content for assessing species-level relationships in other complex taxa [65–69].

PCR was performed in thermocyclers (Eppendorf EP and Eppendorf Pro, Hamburg, Germany) using a variety of protocols and reagents, including EmeraldAmp^®^ DNA Polymerase (Clontech, Takara Bio, New Delhi), optimized for each primer pair. Amplicons were verified under UV light on 4% agarose gels stained with SYBR Safe DNA (ThermoFisher Scientific, Invitrogen, Waltham, MA). PCR clean-up was performed with an AMPure XP (Beckman Coulter, Brea, CA) DNA purification system.

Sanger dideoxy sequencing was conducted with a BigDye Terminator 3.1 cycle sequencing kit (Life Technologies, Carlsbad, CA) according to standard protocols in an ABI 3730XL automated DNA sequencer (Perkin Elmer, Waltham, CA).

Sequences were edited and assembled using Sequencher 5.4.6 (Gene Codes Corporation, Ann Arbor, MI; http://www.genecodes.com) and deposited in GenBank (https://www.ncbi.nlm.nih.gov/genbank; Table S2).

### DNA sequence alignment, statistics and genetic distances

DNA sequences of the three length-variable loci, ITSII, 12S and 16S, were aligned in MAFFT 7.520 [70, 71] using the G-INS-i alignment strategy, which employs global pairwise alignment [72].

DNA sequence alignments were checked by eye using Geneious 2020.2.4 (https://www.geneious.com). Aligned sequences were concatenated using SequenceMatrix 1.9 [73] and exported as a NEXUS interleaved file for phylogenetic analysis. GC content, number of variable positions, and number of parsimony informative sites were calculated for all aligned loci, and intra- and interspecific uncorrected pairwise (*p*) genetic distances calculated for the ITSII, 12S, 16S and COI loci, using MEGA11 [74].

### Phylogenetic analysis

Phylogenetic reconstruction was performed with Maximum Likelihood (ML) using IQ-TREE 2.2.2.6 [75] and Bayesian Inference (BI) using MrBayes 3.2.7 [76, 77]. Command lines for phylogenetic inference with ML are as follows: -*s DNADataset.nex* and/or *-s MorphologyDataset.nex* *-p Partitions.nex* *-m MFP* *-alrt 1000* *-bb 1000* *-bnni*; and with BI are as follows: *mcmc nchains* = *4 print frequency* = *1000 sample frequency = 1000 diagnosing frequency = 1000 burnin = 0.25 savebrlens = yes* (the command* lset nst = mixed* was used prior to the analysis). Final output tree files were visualized and edited using TreeViewer 2.2.0 [78] and Adobe Photoshop CS6.

Morphological and molecular data were analyzed separately and simultaneously. The molecular dataset was analyzed separately as two (i.e., nuDNA and mtDNA) or six (i.e., one per locus) partitions. Molecular substitution models were selected using ModelFinder [79] and the Bayesian Inference Criterion (BIC) for ML, and the reversible jump Markov Chain Monte Carlo algorithm [80], which allows accurate calculation of the Bayes factor for each model while accounting for uncertainty, for BI. An MK evolution model [81], i.e., MK + FQ + ASC + G4, was applied to the morphological matrix.

The morphological and molecular datasets were analyzed simultaneously with the six gene loci and 36 morphological characters divided into seven partitions (one per locus and one for morphology). Simultaneous analysis (total evidence) maximizes the explanatory power of phylogenetic hypotheses in taxa with high levels of morphological stasis [67, 82–86].

One thousand replicates of ultrafast bootstrap approximation (UFBoot [87, 88]), optimized using a hill-climbing nearest neighbor interchange search to reduce the risk of overestimating branch supports [75, 89], were conducted for the ML analysis. An SH-aLRT test [90] with 1,000 replications was also performed to provide more confidence in the nodal support values. The BI analysis was terminated after 5 million generations, when the standard deviation of the split frequencies was below 0.01.

### Hybridization network

A phylogenetic network was assembled by importing the nuDNA trees, obtained from analysis of the concatenated 18S, 28S and ITSII sequences, and the mtDNA trees, obtained from analysis of the concatenated 12S, 16S and COI sequences, into SplitsTree App 6.3.32 [91, 92]. The ‘hybridization network’ option was implemented with the ‘PhyloFusion’ algorithm [93, 94] which searches for the most parsimonious solution (i.e., the minimum number of reticulation events) to explain incompatible splits displayed in each tree topology. The number of terminals included in the analysis was limited to topotypes of each putative species, except for terminals considered to represent hybrid populations, the phylogenetic positions of which conflicted in the nuDNA and mtDNA trees.

### Multivariate morphometric analyses

LDA models were evaluated to estimate the probability that a specimen randomly selected from the morphometrics dataset would be correctly assigned to its putative species. Model performance was assessed by randomly selecting 80% of the observations as the training dataset and using the remaining 20% as a test dataset to validate the model built using the training data (Figure S1). LDA models were constructed using the ‘MASS’ package [95] of R 4.4.3 whereas estimation of posterior probabilities and dataset partitioning were performed using the ‘caret’ package [96]. Due to the relatively small sample size, a single random division might be unrepresentative of the total dataset. The analysis was therefore repeated 10,000 times, each time randomly dividing the data into training and test sets. This resampling approach generated a distribution of posterior probabilities, allowing a more robust evaluation of the predictive accuracy of the model and reducing potential bias associated with a single sampling event. Individuals were classified into nuDNA lineages, mtDNA lineages and total evidence lineages.

### Occurrence records and environmental variables

A dataset of occurrence records for European *Buthus* (Table S7) was assembled by collating and georeferencing localities from multiple sources, including field sites, unpublished data (e.g., Sousa [34]), and verified records from the literature [34, 48–52, 55–62] and iNaturalist. Only records with accurate identifications were included. All species were represented by at least ten occurrence records to ensure robust models [97, 98].

Nineteen bioclimatic variables and an elevation layer at 30-second (ca. 1 km^2^) resolution were obtained from the WorldClim 2.1 database [99] (Table S8). Four bioclimatic variables (i.e., bio8, bio9, bio18, bio19) were omitted due to known spatial artifacts [98, 100]. As *Buthus* scorpions are fossorial, excavating scrapes or burrows under stones or in open ground, soil texture and composition, as well as vegetation cover may affect their locomotion and burrowing ability, ultimately shaping their distributions [101]. Five edaphic variables were therefore added from the Global Soil Information Facilities SoilGrids database at 250 m resolution [102]: soil pH in H_2_O; proportion of sand (> 0.05 mm); silt (≥ 0.002 mm and ≤ 0.05 mm) particles in fine earth fraction (g/100 g); clay (< 0.002 mm) particles in fine earth fraction (g/100 g); and volumetric fraction (vol %) of coarse fragments (> 2.0 mm) (cm^3^/100 cm^3^).

Global remote sensing data for relative humidity (RHU) and the Normalized Difference Vegetation Index (NDVI) were downloaded from Climatologies at High Resolution for the Earth’s Land Surface Areas [103] (https://chelsa-climate.org) and the Copernicus Land Monitoring Service (https://land.copernicus.eu), respectively (Table S7).

### Ecological niche modeling and mapping

Environmental niche models (ENMs) were generated using MaxEnt 3.4.0 [104] with 25,000 random background points sampled across the study area and ten bootstrap replicates as settings [98, 105, 106]. Model performance was evaluated using the Area Under the Curve (AUC) criterion [107]. Percent contribution, variable permutation importance and marginal response curves were analyzed to identify which predictor variables and environmental tolerances most influence habitat suitability and how occurrence probability changes with different environmental conditions [98, 108, 109].

Prior to modeling, correlation analyses among environmental variables were performed using the ‘corrgram’ package [110] in R 4.4.3 and converted to a correlation matrix. Hierarchical cluster analyses based on the correlation matrix were conducted using the Ward-clustering method and plotted as a dendrogram showing similarity among variables [111–114]. The correlation matrix and dendrogram (Figures S2, S3), in which clustered variable pairs or trios with a correlation > 80% were considered collinear [105, 106], were assessed for biological relevance to *Buthus* scorpions. Thirteen variables were selected for the ENMs: bio1, bio2, bio3, bio4, bio12, bio14, bio15, clay, coarse, elevation, pH, sand and silt.

Species occurrence records and ENMs were mapped on a digital elevation layer from DIVA-GIS (https://www.diva-gis.org/data) using QGIS 3.4 (QGIS Geographic Information System; http://www.qgis.org). Paleographical reconstructions of putative colonization routes and corridors, as well as tentative Pleistocene glacial refugia, were adapted from Gómez & Lunt [25], Paulo et al. [26], Médail & Diadema [27], Klesser et al. [35] and Costa et al. [28].

### Environmental association, niche similarity and overlap analyses

Canonical Correlation Analysis (CCA) was conducted using the ‘vegan’ 2.6–10 package in R [115] to detect which environmental factors were strongly associated with species niche divergence or similarity [116]. Species occurrence records were the response variables and the thirteen variables used in the ENMs (Table S7), the explanatory variables. The fit of the CCA model was assessed using *R*² and adjusted *R*² values to estimate variance explained, and 1,000 ANOVA permutation tests to evaluate the significance of the global CCA model, individual CCA axes, and environmental variables. CCA scores, constraints, and explanatory variable loadings were visualized using a biplot to explore niche similarities among candidate species. The influence of each variable was quantified by the length of its corresponding arrow in the biplot. Variables were classified as “determinant” (i.e., solid arrows) or “less determinant” (i.e., dashed arrows) based on a threshold defined as mean arrow length, whereas niche similarity was assessed by the proximity of species. The effects of two abiotic factors (i.e., NDVI and RHU) on species presence were examined using boxplots (Table S7).

Ecological niche interchangeability among species was assessed with niche overlap analyses using the ‘ENMTools’ package in R [117] to test secondary contact hypotheses and predict additional contact areas. Pairwise comparisons were conducted among all species, estimating two metrics of spatial/environmental overlap and resource use similarity: Schoener’s D [118] and the *I*-statistic, based on Hellinger distance [119, 120].

## Results

### Sequence statistics and genetic distances

As in other scorpion taxa [67–69, 83], the COI locus contained the highest number of variable positions (VP) and parsimony-informative (PI) sites, followed by 16S, 12S, ITSII and 28S (Table 2). The 18S contained four VPs but none were PI.

**Table 2:** Length (nucleotide base-pairs, bp) of unaligned and aligned sequences, average percent GC content, number of variable positions (VP), number of parsimony informative sites (PI), and model selected for Maximum Likelihood (ML) using the Bayesian Information Criterion (BIC) in phylogenetic analysis of European species of *Buthus* leach, 1815, based on three nuclear loci, 18S rDNA, 28S rDNA and ITSII, and three mitochondrial loci, 12S rDNA, 16S rDNA and Cytochrome *c* Oxidase Subunit I (COI)

Locus	Unaligned (bp)	Aligned (bp)	GC	VP	PI	BIC
18S	1762	1762	50	4	0	JC
28S	513	513	57	7	2	K2P + FQ + I
ITSII	452	478	52	115	57	SYM + FQ + R2
12S	344	344	26	126	93	TPM2u + F + I + G4
16S	400	487	29	128	100	TPM2u + F + I + G4
COI	1078	1078	38	297	248	GTR + F + I + G4

**Fig. 2:**
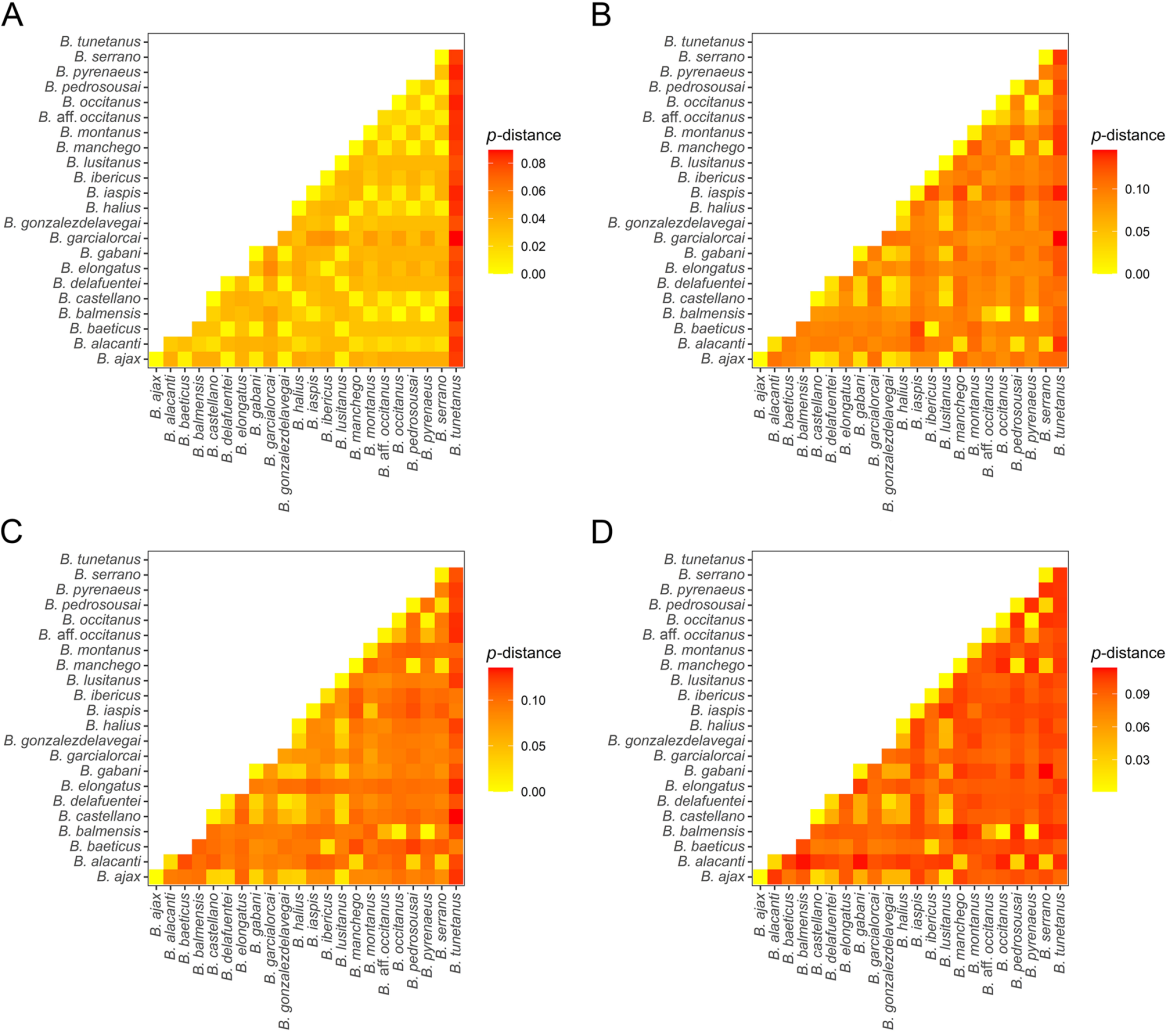
Heatmaps depicting pairwise genetic distances for variable loci in putative species of European *Buthus* Leach, 1815, with the Tunisian species, *Buthus tunetanus* (Herbst, 1800) for context. **A** *p*-distances of Internal Transcribed Spacer (ITSII). **B** *p*-distances of 12S rDNA (12S). **C** *p*-distances of 16S rDNA (16S). **D** *p*-distances of Cytochrome *c* Oxidase Subunit I (COI). Raw data in Tables S3–S6

Mean pairwise distances in the nuclear (ITSII) and mitochondrial (12S, 16S, and COI) loci among the species of *Buthus* were comparable to interspecific distances reported for other related buthid genera, e.g., *Buthacus* Birula, 1908, *Hottentotta* Birula, 1908 and *Odontobuthus* Vachon, 1950 [67, 86, 121, 122] (Fig. 2; Table S3). Genetic divergence in the nuclear ITSII locus between the European and Tunisian samples was notably higher than among the European samples (Fig. 2A; Table S3), suggesting an interruption of gene flow across the Strait of Gibraltar. However, some mtDNA distances among European species were comparable to those between European and Tunisian samples (Figs. 2B–D; Tables S4‒S5). Whereas little to no variation was observed in the nuclear ITSII locus across extensive parts of southeastern France and the Iberian Peninsula, substantial differences were evident in the mitochondrial loci, even among geographically proximate populations, implying marked regionalization of the maternally inherited mtDNA (Tables S3‒S5).

### Phylogenetic analyses

All analyses recovered a monophyletic group comprising the European species of *Buthus*. The Tunisian species, *B. tunetanus*, was consistently placed sister to the European clade, and the Moroccan species, *B. maroccanus*, sister to the clade comprising *B. tunetanus* and the European species.

**Fig. 3:**
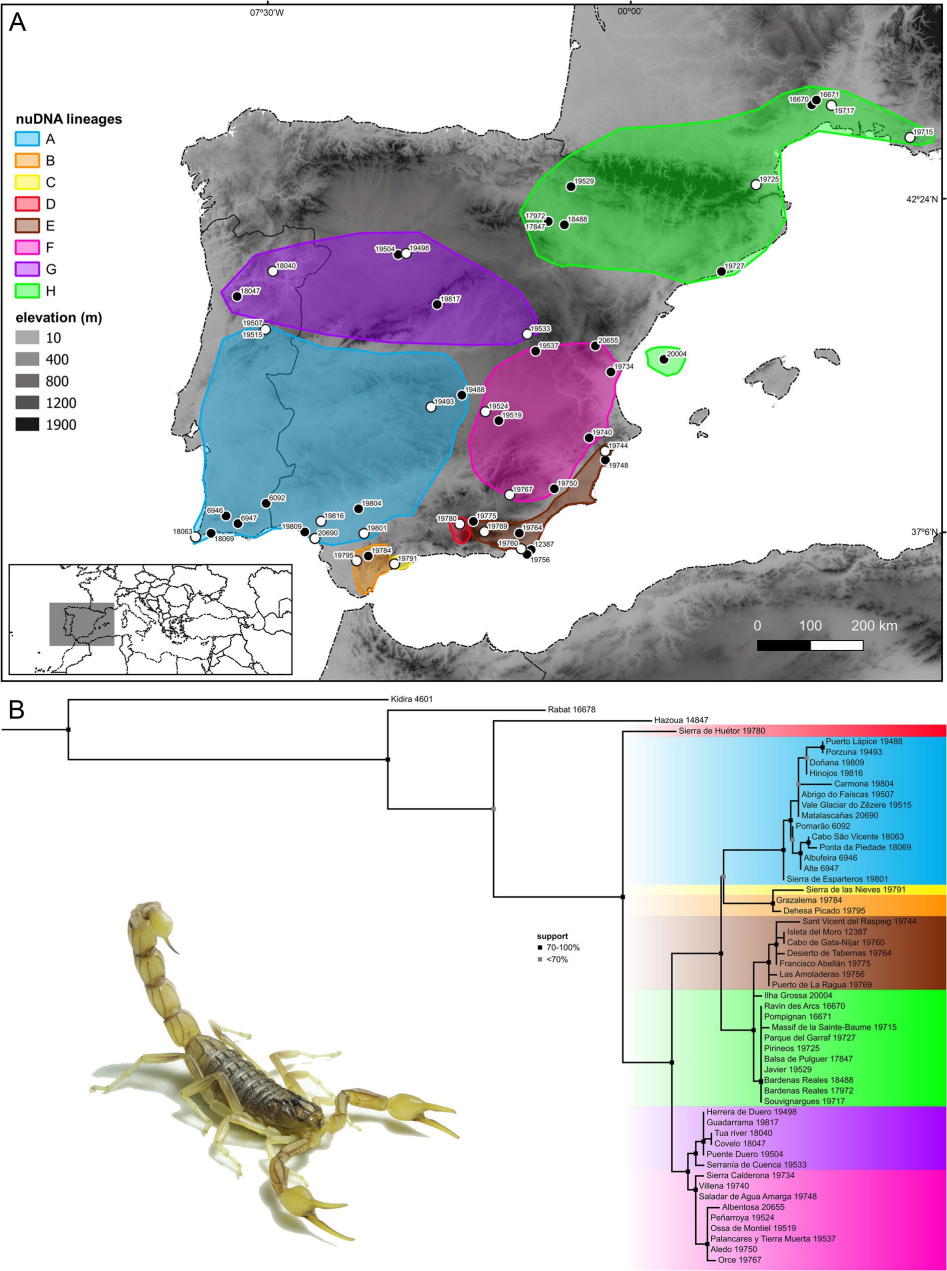
**A** Map of the Iberian Peninsula and southwestern France with sample locations and approximate distributions of distinct nuclear DNA (nuDNA) lineages of European species of *Buthus* Leach, 1815. White circles indicate type localities. **B** nuDNA phylogeny of European species of *Buthus* obtained by Maximum Likelihood (ML) analysis of 2753 bp of DNA sequence from three nuclear gene loci (18S rDNA, 28S rDNA and ITSII) for 54 samples. Georeferences and GenBank accession codes in Table S2. Photo courtesy of Ignazio Avella

**Fig. 4:**
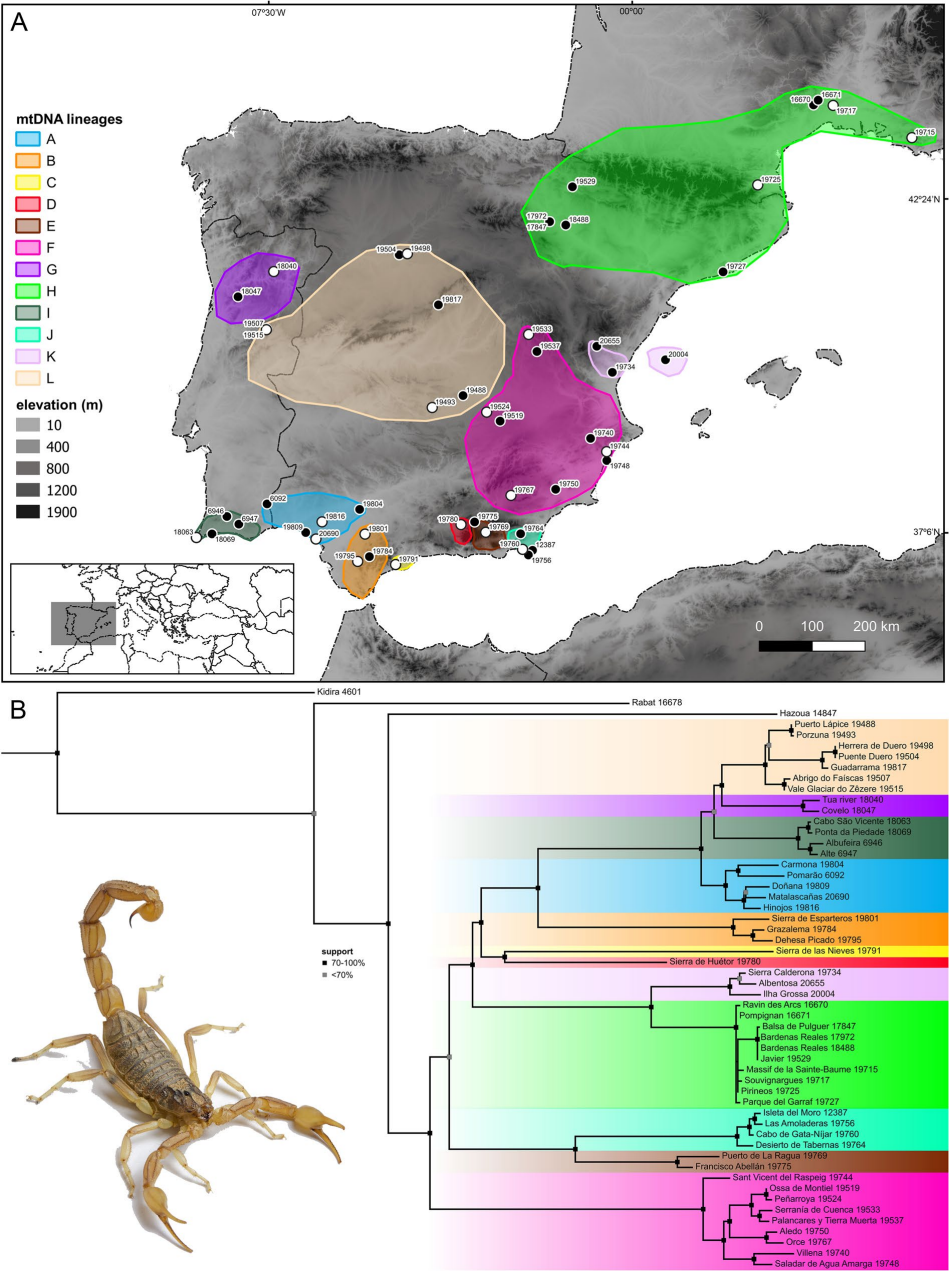
**A** Map of the Iberian Peninsula and southwestern France with sample locations and approximate distributions of distinct mitochondrial DNA (mtDNA) lineages of European species of *Buthus* Leach, 1815. White circles indicate type localities. **B** mtDNA phylogeny of European species of *Buthus* obtained by 1909 bp of DNA sequence from three mitochondrial gene loci (12S rDNA, 16S rDNA, and COI) for 54 samples. Georeferences and GenBank accession codes in Table S2. Photo courtesy of Ignazio Avella

Tree topologies obtained by separate analyses of the nuclear and mitochondrial loci were incongruent in the placement of certain samples and differed from the topology obtained by simultaneous (total evidence) analysis of the molecular and morphological data concerning relationships among the major lineages (Figs. 3B, 4B and 7B). The topology obtained by separate analysis of the nuDNA recovered eight lineages (A–H) spanning extensive geographical areas (Fig. 3), with high support values for internal branches but moderate to low support for terminal branches, whereas the topology obtained by separate analysis of the mitochondrial DNA recovered twelve lineages (A–L) occupying more restricted geographical areas, particularly in southern Portugal and Spain (Fig. 4), with high support values for internal branches and high to moderate support for terminal branches.

Topologies obtained by simultaneous analysis with BI and ML were almost identical, differing primarily in the phylogenetic position of *B. garcialorcai*, which was placed sister to *B. elongatus* in the analysis with BI, apparently a long-branch attraction artifact [123], and as sister to all other European species of *Buthus* in the analysis with ML. BI posterior probabilities were consistently higher (always > 90%) than bootstrap support values in the analysis with ML (Figs. 3B, 4B and 7B). Although high Bayesian posterior probabilities values are often interpreted as strong evidence for clade validity, Bayesian methods have been shown to exhibit overconfidence, particularly under model misspecification [124]. In contrast, the ML bootstrap approach is more conservative and therefore potentially less likely to strongly support an incorrect phylogenetic hypothesis [124–126]. Besides, sampling gaps, ILS, and introgression may have contributed to the lower support or topological uncertainty observed in certain clades. Nonetheless, both analyses recovered a clade comprising the European species of *Buthus* and nine subclades, corresponding to the species recognized by Blasco-Aróstegui et al. [43]: three species widespread across the northwestern-central and central-northeastern Iberian Peninsula to southwestern France, i.e., *B. halius*, *B. manchego* and *B. occitanus*, and six species with more restricted distributions, occupying distinct basins and mountain ranges in the topographically complex southern Iberian Peninsula, i.e., *B. delafuentei*, *B. elongatus*, *B. garcialorcai*, *B. iaspis*, *B. ibericus* and *B. montanus* (Fig. 7). The tree topology obtained from simultaneous analysis with ML (log − 16437.1711) was selected as the preferred hypothesis (Fig. 7B), based on morphological character optimization [43]. The inclusion of morphology increased the bootstrap support values and clarified the relationships among several clades.

### Hybridization analyses

**Fig. 5:**
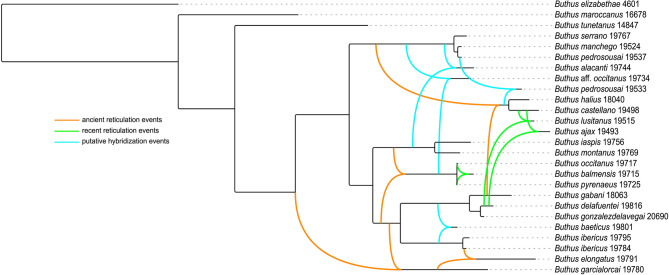
A. Hybridization network illustrating mito-nuclear discordance in nuDNA and mtDNA phylogenies of putative European species of *Buthus* Leach, 1815 (Table 1). Coloured reticulation events indicate most parsimonious explanation. Orange lines indicate ancient reticulation events; green lines, recent reticulation events; and blue lines, hybridization events manifest as intermediate morphological character combinations. Georeferences and GenBank accession codes in Table S2

**Fig. 6:**
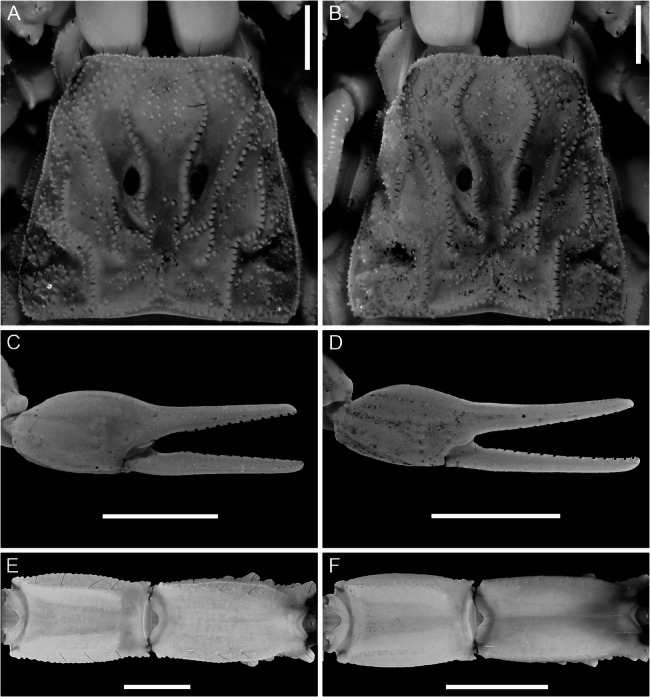
Examples of intermediate morphological character combinations observed in putative cases of hybridization. **A** *Buthus occitanus* (Amoreux, 1789), ♂ (AMNH [LP 19717]), Souvignargues, Occitanie, France, carapace with incomplete median ocular carinae. Same carination pattern as *Buthus alacanti* Teruel & Turiel, 2020 = *Buthus manchego* Teruel & Turiel, 2020, ♂ (AMNH [LP 19744]), Sant Vicent del Raspeig, Com. Valenciana, Spain. **B ***Buthus occitanus* (Amoreux, 1789), ♂ (AMNH [LP 19734]), Sierra Calderona, Com. Valenciana, Spain, carapace with complete median ocular carinae. Same carination pattern as *Buthus manchego* Teruel & Turiel, 2020, ♂ (AMNH [LP 19524]), Embalse de Peñarroya, Castilla-La Mancha, Spain. **C**,** D ***Buthus pedrosousai* Teruel & Turiel, 2021= *Buthus manchego* Teruel & Turiel, 2020, pedipalp chela, dorsolateral aspect. **C** ♂ (AMNH [LP 19533]), Serranía de Cuenca, Castilla-La Mancha, Spain, chela fingers curved basally, emarginate when closed. **D** ♂ (AMNH [LP 19537]), Torcas de Palancares y Tierra Muerta, Castilla-La Mancha, Spain, chela fingers sublinear basally, not emarginate when closed. **E** *Buthus baeticus* Teruel & Turiel, 2020 = *Buthus ibericus* Lourenço & Vachon, 2004, ♀ (AMNH [LP 19801]), Sierra de Esparteros, Andalucía, Spain, metasomal segments IV and V, dorsal aspect, illustrating presence of setae. **F** *Buthus ibericus*, ♀ (AMNH [LP 19795]), Dehesa Picado, Andalucía, Spain, metasomal segments IV and V, dorsal aspect, illustrating absence of setae

The hybridization network analysis revealed seven reticulation events, ancient and recent (Fig. 5). Putative hybridization events were also identified between four species, sometimes manifested phenotypically as populations with intermediate character combinations (Fig. 6), suggesting recent or historical gene flow.

### Multivariate morphometric analyses

Consistent with the sexual dimorphism typical of *Buthus* species, greater morphological divergence was observed in the LDAs of adult males than adult females. Several distinct clusters were evident in the male plots (Figs. 8A, C, E) compared to the female plots, in which there was more overlap (Figs. 8B, D, F). LDAs of morphometric data for males revealed four distinct clusters when grouped by nuDNA lineages and at least eight distinct clusters when grouped by mtDNA lineages or by the nine valid species. Whereas five distinct clusters, corresponding to *B. elongatus*, *B. iaspis*, *B. ibericus*, *B. montanus* and *B. occitanus*, were consistently recovered in the male plots, only one cluster, corresponding to *B. garcialorcai*, was consistently recovered in the female plots.

### Ecological niche modeling

Model validation results were generally robust, with AUC > 0.95, AICc from 24.21 to 3898.89, and a 10% omission rate, from 0.0 to 0.094, in all species (Table S9). Some models predicted small areas with no occurrence records which may account for false positives [97]. Habitat suitability predictions, permutation analyses and response curves revealed differences in bioclimatic and soil variable dependence among the nine valid species (Fig. 9; Table S10). The most influential variables in the species models were three bioclimatic variables, bio14 (67%), bio15 (56%) and bio12 (56%), along with elevation (67%), pH (44%) and coarseness (44%) (Table S10).

### Environmental associations, niche similarity and spatial overlap analyses

The environmental CCA model was globally significant (ANOVA, *df* = 13, χ^2^ = 2.87, *F* = 11.36, *p* < 0.001) and explained 36% of the variance (adjusted *R*^2^ = 0.33). Both CCA axes were significant and explained 31.4% of the variance (CCA1 ->ANOVA, *df* = 1, χ^2^ = 0.9, *F* = 47.19, *p* < 0.001) and 21.8% of the variance (CCA2 ->ANOVA, *df* = 1, χ^2^ = 0.63, *F* = 32.75, *p* < 0.001), respectively. Only bio14 and bio15 exhibited correlation coefficients greater than |0.6| on CCA1, whereas only bio2 and bio4 exhibited correlation coefficients greater than |0.55| on CCA2 (Table S11).

Permutation tests revealed statistical significance within all explanatory environmental variables (*p*-value < 0.05). The most determinant variables in species niche similarities were bio15, bio14, bio4, bio2 and elevation (continuous arrows), whereas pH, silt, bio3, bio12, bio1, sand, coarse and clay where less determinant (dashed arrows).

Based on Schoener’s D, low to moderate spatial niche overlap was observed among all valid species of *Buthus*, with higher values among the more geographically restricted, southern species, e.g., *B. ibericus* vs. *B. elongatus* or *B. garcialorcai* vs. *B. montanus*, compared with the more widespread central and eastern species, e.g., *B. halius*, *B. manchego* and *B. occitanus*, which exhibited considerably lower values (Table 3). The pattern based on the *I*-statistic was identical, except that the values indicated moderate to high niche overlap (Table 3). The lowest niche overlap (i.e., most different niches) was between *B. ibericus* and *B. manchego* (D = 0.01, *I* = 0.03) whereas the highest overlap (i.e., most similar niches) was between *B. garcialorcai* and *B. montanus* (D = 0.66, *I* = 0.88).

**Table 3:** Niche spatial overlap among valid species European species of *Buthus* Leach, 1815 based on Schoener’s D (D) and the *I*-statistic (*I*) metrics. Values closer to 0 indicate low overlap, whereas values closer to 1 indicate high overlap. Abbreviations: *del*, *Buthus delafuentei* Teruel & Turiel, 2020;* elo*, *Buthus elongatus* Rossi, 2012; *gar*, *Buthus garcialorcai* Teruel & Turiel, 2020; *hal*, *Buthus halius* (C.L. Koch, 1839); *ias*, *Buthus iaspis* Teruel & Turiel, 2022; *ibe*, *Buthus ibericus* Lourenço & Vachon, 2004; *man*, *Buthus manchego* Teruel & Turiel, 2020;* mon*, *Buthus montanus* Lourenço & Vachon, 2004;* occ*,* Buthus occitanus* (Amoreux, 1789)

		***del***	***elo***	***gar***	***hal***	***ias***	***ibe***	***man***	***mon***	***occ***
**D**	***del***	**-**								
	***elo***	0.1892	**-**							
	***gar***	0.0773	0.1596	**-**						
	***hal***	0.1070	0.0787	0.2135	**-**					
	***ias***	0.0357	0.0265	0.2313	0.0591	**-**				
	***ibe***	0.1838	0.5275	0.1037	0.0989	0.0117	**-**			
	***man***	0.0145	0.0077	0.1112	0.1121	0.1101	0.0057	**-**		
	***mon***	0.0542	0.1015	0.6670	0.2677	0.2819	0.0608	0.1571	**-**	
	***occ***	0.0117	0.0035	0.0238	0.1101	0.0226	0.0059	0.1844	0.0755	**-**
***I***	***del***	**-**								
	***elo***	0.4107	**-**							
	***gar***	0.2111	0.3861	**-**						
	***hal***	0.2501	0.2287	0.4547	**-**					
	***ias***	0.1631	0.0842	0.4691	0.2165	**-**				
	***ibe***	0.4162	0.7809	0.2829	0.2622	0.0501	**-**			
	***man***	0.0618	0.0332	0.3128	0.3220	0.3187	0.0291	**-**		
	***mon***	0.1670	0.2872	0.8897	0.5441	0.5429	0.1987	0.4215	**-**	
	***occ***	0.0488	0.0213	0.0996	0.3187	0.0877	0.0264	0.4060	0.1888	**-**

## Discussion

### Hybridization, introgression and incomplete lineage sorting

Nuclear markers are important for clarifying phylogenetic relationships [21, 29, 127]; their omission from phylogenetic analyses may obscure the detection of introgression and admixture between populations [1, 3, 8, 11]. In the present study of Iberian *Buthus* scorpions, a pattern of mito-nuclear discordance was revealed by incongruent nuclear and mitochondrial gene tree topologies (Figs. 4B, 5B and 6A). Although detected in early phylogenetic and phylogeographic studies of *Buthus* [38–40], this discordance was ignored in subsequent studies [33, 35, 41, 42, 53], which relied exclusively on mtDNA markers, specifically 16S and COI. Maternally inherited mtDNA generally evolves at a faster pace and may result in incomplete or misleading conclusions about species relationships [1, 2].

Patterns of mito-nuclear discordance in *Buthus* species are consistent with the geographical distributions of nuDNA lineages, suggesting mitochondrial capture following secondary contact [21, 29, 31, 128–130] (Figs. 3A, 5A and 10). Male-biased gene flow, previously suggested by Gantenbein & Largiadèr [38] and Habel et al. [41], may explain mitochondrial capture by nuDNA lineages. As in many other arachnid taxa, including scorpions [131–134], *Buthus* males are vagile, travelling greater distances in search of food or mates, whereas females are sedentary [44, 45]. Limited female dispersal tends to preserve the geographical structure of maternally inherited mtDNA haplotypes whereas male-mediated gene flow tends to homogenize nuclear genetic variation [21, 38, 129, 132, 134]. Therefore, the combination of female philopatry and male-biased dispersal creates mito-nuclear discordance and the coexistence of multiple mitochondrial lineages within a single nuDNA lineage [127, 128]. Although gene flow is expected among closely related or genetically similar lineages, this is not always the case. For example, the *halius* and *manchego* complexes, which represent distinct nuDNA and mtDNA lineages E and F (Figs. 3B and 4B), also display a pattern of historical reticulation (Fig. 5). Evidence of genetic exchange between lineages, despite profound mitochondrial divergence (*p*-distances of 9–10% in 12S, 16S and COI; Fig. 2; Tables S4‒S6) raises the question: how can profoundly divergent mtDNA lineages interbreed? Comparison of the male intromittent organ (hemispermatophore) among species of *Buthus* revealed high levels of morphological similarity, even among distantly related lineages [43]. *Buthus* hemispermatophores do not appear to provide prezygotic isolation [135], likely allowing gene flow between genetically divergent lineages, regardless of deep mitochondrial divergence [4, 6, 23, 47]. This hypothesis is supported by the unique mitofusion process in *Buthus* [40, 46]. This phenomenon probably increases the opportunity for homologous (intermolecular) or non-homologous (intramolecular) recombination of mtDNA, facilitating the persistence of ancient and diverse mtDNA lineages while allowing for hybridization [40].

**Fig. 7:**
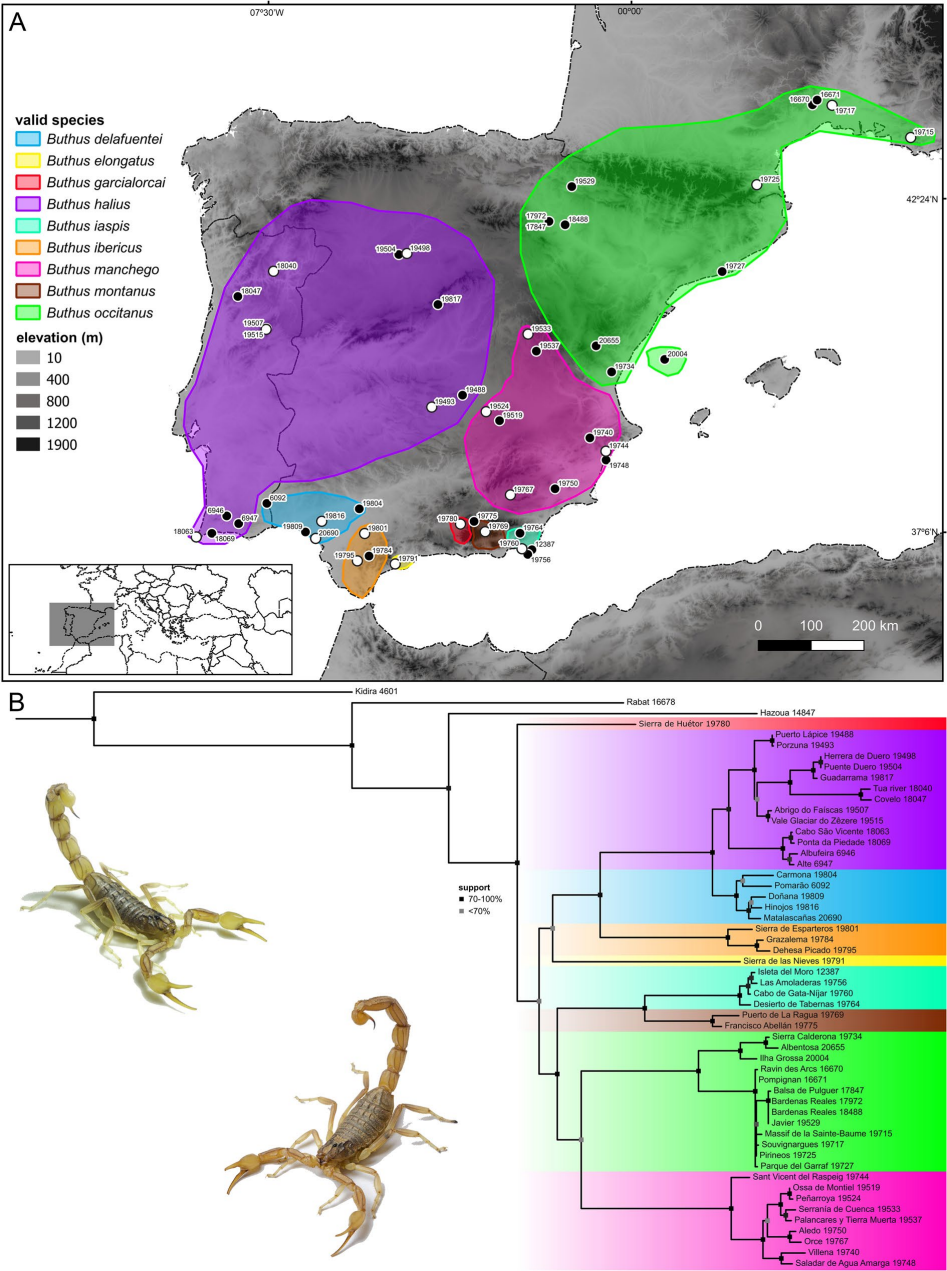
**A** Map of the Iberian Peninsula and southwestern France with sample locations and approximate distributions of valid European species of *Buthus* Leach, 1815. White circles indicate type localities. **B** Phylogeny obtained by Maximum Likelihood (ML) analysis of 36 morphological characters and 4575 bp of DNA sequences from three nuclear (18S rDNA, 28S rDNA and ITSII) and three mitochondrial (12S rDNA, 16S rDNA, and COI) gene loci, for 54 samples. Georeferences and GenBank accession codes in Table S2. Photos courtesy of Ignazio Avella

**Fig. 8:**
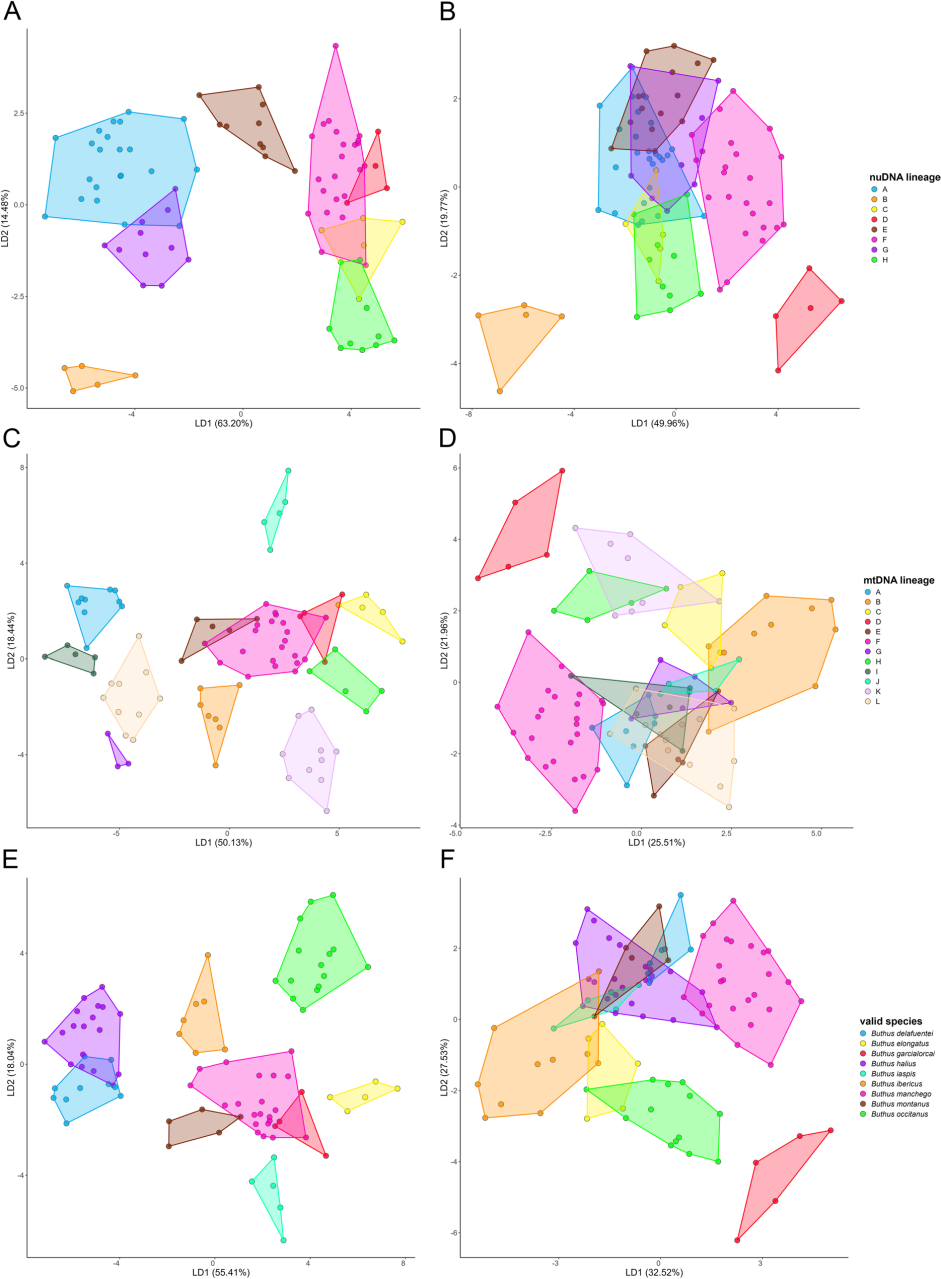
Linear Discriminant Analysis plots for males (**A**,** B**,** C**) and females (**D**,** E**,** F**) of European species of *Buthus* Leach, 1815, based on 25 morphometrics for 189 adult specimens grouped by unique nuDNA lineage (**A**,** B**), unique mitochondrial lineage (**C**,** D**), and species (**E**,** F**)

**Fig. 9:**
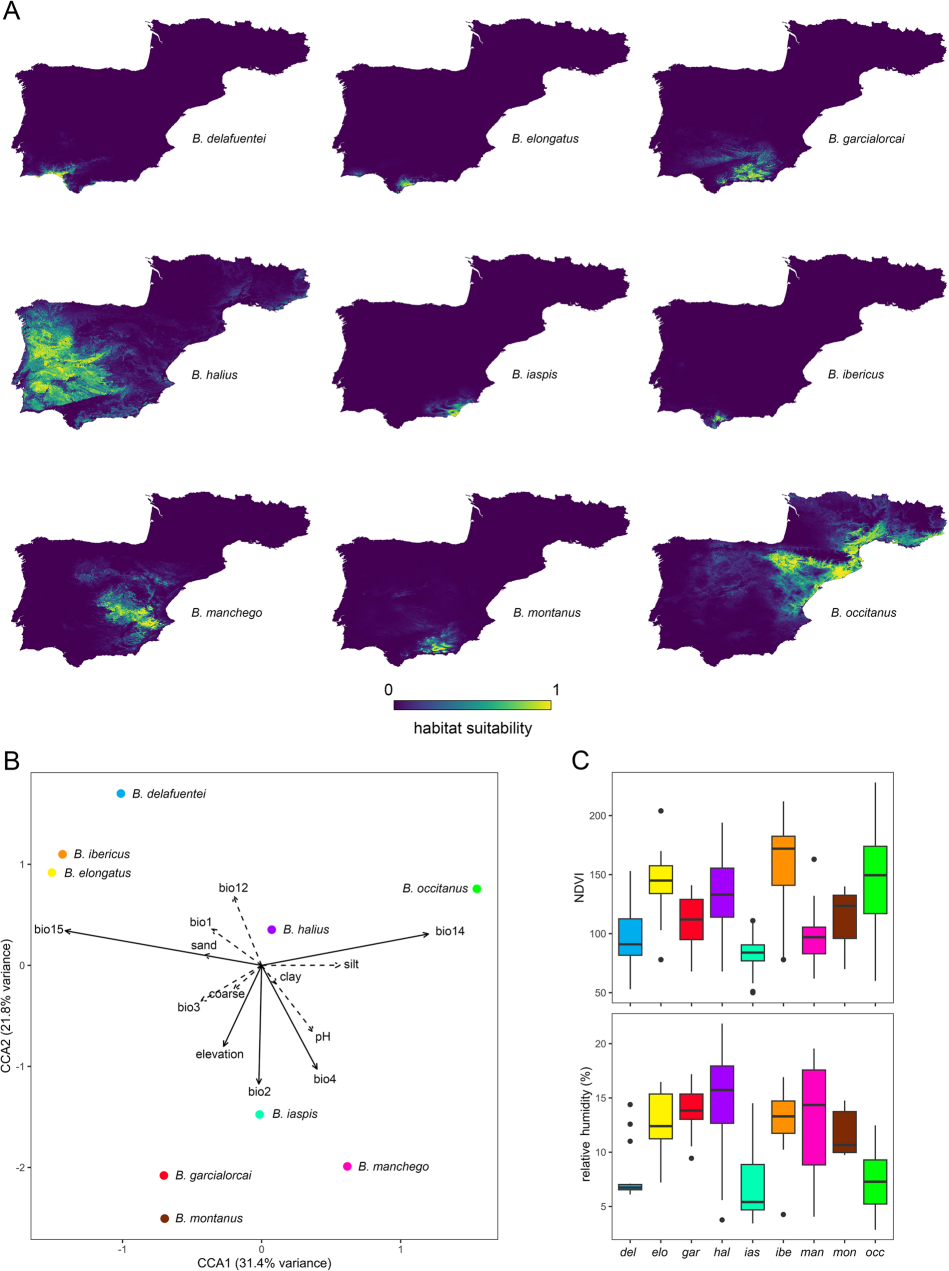
Environmental space occupied by European species of *Buthus* Leach, 1815. **A** Environmental Niche Models (ENMs) predicted based on bioclimatic and soil layers. **B** Canonical Correlation Analysis (CCA) biplot of relationship between species and environmental and soil variables used in ENM. Arrows indicate biplot loadings, with solid and dashed arrows representing most determinant and least determinant variables, respectively. **C** Boxplots illustrating species response to remote sensing data of NDVI and RHU

Despite the lack of conclusive evidence compared to introgressive hybridization, ILS remains a plausible alternative hypothesis to explain part of the diversification of *Buthus* in the Iberian Peninsula [41, 136–138]. For example, populations within the *halius* complex exhibit at least four distinct mtDNA haplotypes (i.e., A, G, I and L) but only two nuDNA lineages (A and G), resulting in the recognition of only two fully differentiated species (Figs. 3, 4 and 7‒9; [43]). Such temporal asymmetry in lineage sorting suggests that mtDNA has undergone more rapid differentiation, whereas nuDNA still retains ancestral polymorphisms—likely because genetic drift has not yet had sufficient time to fix nuclear loci before subsequent divergence events in mitochondrial loci [137]. A similar pattern is observed in the *occitanus* complex, where *B. iaspis* displays deep mtDNA divergence from *B. montanus*, yet both share nearly identical nuDNA profiles (Figs. 3, 4 and 7). Likewise, the *B. occitanus* population from Columbretes Islands (i.e., 20004; Figs. 3, 4 and 7) exhibits mitochondrial differentiation despite sharing nuDNA with mainland populations (i.e., 17972, 17847, 18488, 19529, 19715, 19717, 19725 and 19727; Figs. 3, 4 and 7).

Discrepancies between mitochondrial and nuclear markers, and more broadly between gene trees and species trees (Figs. 3, 4 and 7), highlight the potential role of both introgressive hybridization and ILS in shaping current phylogenetic patterns in Iberian *Buthus* and underscore the importance of further integrative research to completely understand the evolutionary processes occurring at shallow time depths.

### Biogeographical patterns

**Fig. 10:**
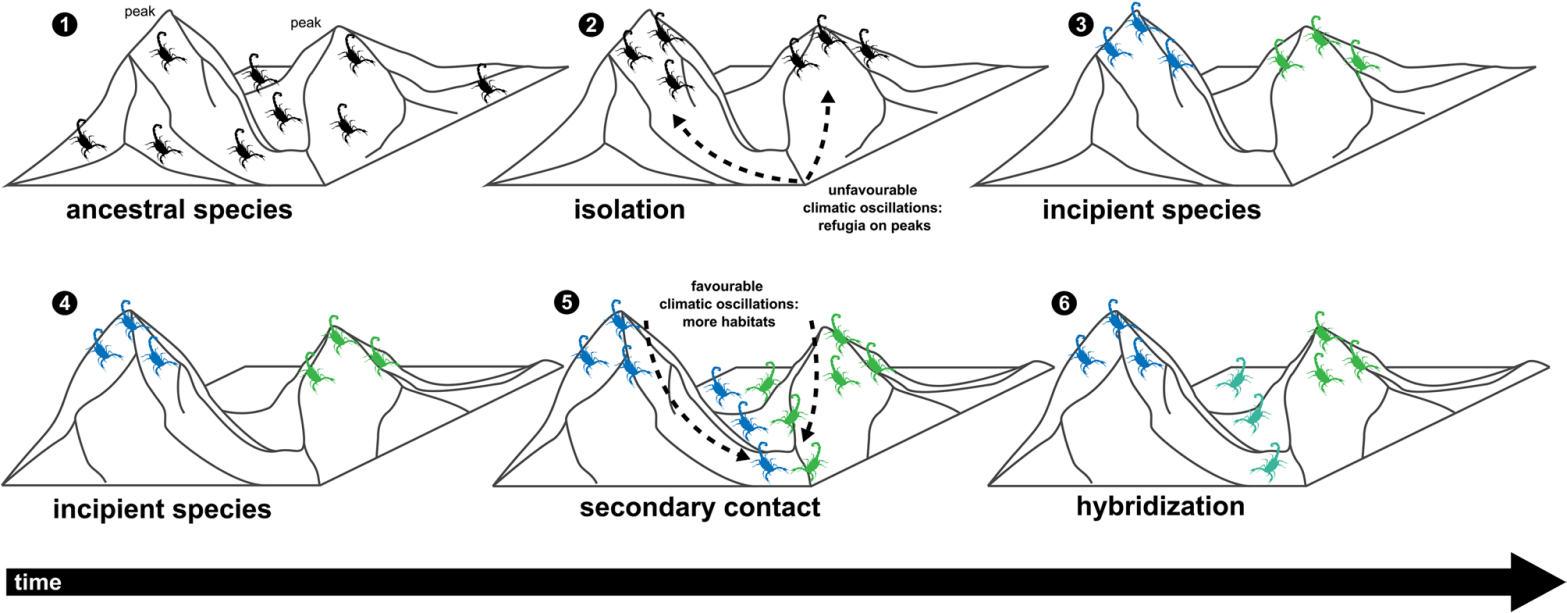
Hypothetical model of speciation for European species of *Buthus* Leach, 1815 adapted from ©Andrew Z. Colvin, CC BY-SA 4.0: Prolonged isolation of ancestral species populations on mountains, i.e., glacial refugia (1‒3); secondary contact between genetically diverged populations facilitated by male-biased dispersal in favourable climatic periods, e.g., interglacials (4‒5); hybridization through disassortative mating when reproductive isolation not fully developed (6)

The Late Miocene, particularly the Tortonian (11.6–7.2 Mya) and Messinian (7.2–5.3 Mya) stages [34, 35, 41, 139] was among the most significant periods in the diversification of Iberian taxa. Sea-level fluctuations and the formation of land bridges and ecological corridors, e.g., the Rifian, Betic or Guadalhorce corridors (Fig. 1A), played a crucial role in faunal exchanges with North Africa and dispersal across the Iberian Peninsula [26, 28]. Whereas these corridors allowed gene flow among populations and incipient species, changing climate and sea levels, e.g., the MSC and Pleistocene climatic oscillations (Figs. 1B–D), subsequently fragmented populations, leading to isolation and differentiation (Fig. 10). Pleistocene glaciations (2.58 Mya–11,700 years) also left a genetic imprint on the Iberian biota, with prolonged periods of isolation in climatic refugia causing pronounced intraspecific divergence in mtDNA [19, 20, 22]. As the climate warmed and geographical barriers (e.g., rivers, lakes, or ice sheets) disappeared, previously isolated populations came into secondary contact, leading to hybridization when reproductive isolation was not fully established [8, 18, 21, 29, 31, 130] (Fig. 10).

Comparison of the geographical areas occupied by Iberian *Buthus* lineages E and F (Figs. 4A and 5A) provides an example. The present distribution of nuDNA lineage E matches the historical extent of the Betic and Guadalhorce corridors [26, 28] (Figs. 1 and 3A;), suggesting the entire southeastern area may have been colonized, allowing panmixis prior to isolation in glacial refugia, as suggested by the mtDNA [25, 27] (Figs. 1, 5A and 10). In contrast, nuDNA lineage F and mDNA lineage F occupy a broader, central-eastern area, overlapping with part of the region occupied by nuDNA lineage E. As the climate warmed and geographical barriers disappeared, populations of both lineages may have expanded their distributions and come into secondary contact. If males from one nuDNA lineage, e.g., lineage E (‘father species’ *sensu* Wirtz [140]) mated with females from a different mtDNA lineage, e.g., lineage F (‘mother species’ *sensu* Wirtz [140]), a pattern of mito-nuclear discordance would result. In such a scenario, hybridization and introgression results from secondary contact between previously isolated lineages due to male-biased dispersal facilitated by historical events (in this case, climate fluctuation; Figs. 1 and 10).

### Morphological stasis vs. variation in contact zones

As Iberian *Buthus* species appear to have hybridized in various areas (Figs. 3, 4 and 5), the impact on intraspecific morphological variation, especially in putative contact zones, cannot be ignored. Taxonomic confusion arose in several Iberian taxa, e.g., vipers, in which intermediate combinations of morphological characters arose from admixture between distinct species [18], a pattern also evident in several Iberian *Buthus*, the most notable being the species described as *Buthus pedrosousai* Teruel & Turiel, 2021. Populations assigned to *B. pedrosousai* from two locations, the Serranía de Cuenca (i.e., 19533) and Torcas de Palancares y Tierra Muerta (i.e., 19537), shared the same mtDNA lineage F (Fig. 4) but represented distinct nuDNA lineages G and F (Fig. 3), respectively, and were distinguished by a striking morphological difference. The pedipalp chela manus is globose and the chela fixed and movable fingers curved basally and emarginate when closed in males from the Serranía de Cuenca whereas the manus is slender and the fingers sublinear basally, not emarginate when closed in males from Torcas de Palancares y Tierra Muerta, consistent with the morphology of the males of nuDNA lineages G and F, respectively (Figs. 3A, 4A and 6C and D). Although sexual selection (e.g., disassortative mating) could explain this variation, such intraspecific variation in pedipalp chela shape is atypical and usually fixed in scorpion species, including *Buthus* [54, 86, 141, 142]. The distinct chela shape of *Buthus* males from the Serranía de Cuenca led Teruel & Turiel (2021b) to describe this population as a new species. However, the results presented here suggest a different interpretation. As the geographical distributions of the nuDNA lineage G and mtDNA lineage F are presently contiguous (Figs. 3 and 4), males of the nuDNA lineage G may have colonized and interbred with females of the mtDNA lineage F leading to nuclear homogenization through mitochondrial capture. Similar patterns of mitochondrial introgression and nuclear homogenization, resulting in intermediate phenotypes, have been reported across contact zones of Iberian fire salamanders and *Mesocarabus* beetles [8, 21].

Other likely cases of hybridization following secondary contact between distinct nuDNA and mtDNA lineages of *Buthus* suggest a widespread phenomenon. Overlapping distributions, incongruence between nuDNA and mtDNA tree topologies, and intermediate combinations of morphological characters largely consistent with nuDNA lineages were also identified in the populations assigned to the putative species *Buthus alacanti* Teruel & Turiel, 2020 and *Buthus baeticus* Teruel & Turiel, 2020, and some populations of *Buthus occitanus* (Amoreux, 1789) (Figs. 6A, B, E, F; Blasco-Aróstegui et al. [43]). The correlation between morphology and nuDNA lineages suggests that species of *Buthus* are best delimited in accordance with the latter (Figs. 3B, 7B and 8A and E), as noted in other taxa (e.g., [144]). Mounting evidence suggests that morphology conflicts with species delimitation based on mtDNA lineages when introgressive hybridization is prevalent [8, 11, 18, 40, 143, 144]. By relying solely on mtDNA trees and morphometrics (Figs. 4 and 8B and C), previous studies [34, 41, 48–50, 56, 58–62] overestimated the species diversity of Iberian *Buthus*. For example, although genetically divergent mtDNA lineages J–L could be interpreted as a complex of morphologically similar cryptic species, this interpretation ignores their intricate evolutionary history, which includes multiple reticulation events that probably blurred morphological differentiation (Figs. 4, 5 and 6). Morphological variation within mtDNA lineages B, F, and K could likewise be misconstrued as evidence of speciation, were it not for the mito-nuclear discordance among some of these lineages. A more nuanced approach, considering evidence from genomics, morphology and ecology is essential for refining species limits with taxa with complex evolutionary histories, like *Buthus* [43].

### Ecological factors

Ecological niche modeling revealed several drivers of habitat suitability in Iberian *Buthus*, with precipitation (i.e., seasonality and driest month) and annual temperature being the most common (Table S10). Although these scorpions predominantly occupy xeric, sparsely vegetated shrublands or open-canopy pine forests [58–61] (Fig. 9C), some species, such as *B. elongatus* and *B. ibericus*, thrive in mixed forests characterized by high seasonal precipitation, e.g., in the Sierra de Grazalema and Sierra de Las Nieves, whereas others, like *B. delafuentei*, *B. iaspis* and *B. occitanus*, inhabit dune systems and semi-deserts with the lowest relative humidity and precipitation in Europe, e.g., Bardenas Reales, Desierto de Tabernas, and Doñana (i.e., samples 17847, 19764 and 19809 respectively; Figs. 7A and 9A and C). Soil composition also influences the distributions of *Buthus*. Coarse substrata and high alkalinity are preferentially selected by some species (Table S10), e.g., *B. garcialorcai* occurs in silt deposits derived from glacial grinding [25, 27, 145] (Fig. 1D) and *B. manchego* prefers high salinity soils (e.g., salt marshes and sodic sites). *Buthus delafuentei*, a species restricted to dune systems and sandy deposits of the Guadalquivir Basin (Figs. 1, 7 and 9; Table S10), exhibits positive psammotropism and psammophilous adaptations to soft substrates [43], an example of speciation driven by substratum specialization, consistent with the “Effect Hypothesis” [101, 146, 147].

Elevation also contributed to habitat differentiation in the ENMs, implying speciation by niche divergence across altitudinal gradients [41, 148, 149]. For example, the sister species, *B. iaspis* and *B. montanus*, exhibit elevational segregation (Figs. 7 and 9), with *B. iaspis* restricted to valleys and basins and *B. montanus* to mountain peaks [43] (Figs. 9A and B and 10).

Despite the narrow habitat requirements of *Buthus* species detected by the ENMs (Table S10; Fig. 9A), much spatial overlap exists among the modelled projections (Figs. 9A, B; Table 3). Southern, range-restricted species inhabiting adjacent mountain ranges and valleys exhibited higher niche overlap than more widespread central and eastern species (Figs. 7 and 9; Table 3). This overlap suggests that a level of ecological continuity in geographically proximate areas of the Iberian Peninsula [150] may have enabled these scorpions, with their similar habits, e.g., generalist predation, burrowing [44, 45], to colonize suboptimal habitats, provided deviations from the realized niche remained within a tolerable range [151]. Many other examples of arthropods rapidly colonizing broader areas of Europe and the Iberian Peninsula via homogeneous climate islands and corridors (i.e., potentially colonizable areas) have been documented [113, 114, 152].

Secondary contact zones detected by hybridization analyses were also implied by the analysis of environmental space (Fig. 9; Table 3). ENMs, CCA analysis and niche overlap metrics revealed low to moderate overlap between predicted distributions for *B. delafuentei* and *B. ibericus*, as well as for *B. manchego* and *B. occitanus* (Fig. 9A, B; Table 3). The secondary contact zone between *B. halius* and *B. manchego* was less predictable (D = 0.11, *I* = 0.32; Table 3), probably due to the small number of identifiable records of both species in the zone of overlap. All environmental space analyses also recovered high niche similarity and overlap between *B. elongatus* and *B. ibericus*, and between *B. garcialorcai* and *B. montanus* (Fig. 9; Table 3). Secondary contact zones may exist between these species, but their identification requires further sampling. The sister species *B. delafuentei* and *B. halius* exhibited very low overlap (D = 0.1, *I* = 0.25; Table 3), reinforcing the evidence for their evolutionary distinctiveness.

## Conclusions

Repeated cycles of habitat expansion and contraction, caused by glaciation, climate change, and sea level fluctuations, have left a lasting imprint on the biota of the Iberian Peninsula. Consistent with other Iberian invertebrates as well as poikilothermic vertebrates, isolated, range-restricted lineages of *Buthus* scorpions are concentrated in the south of the Peninsula, where mountain barriers and glacial refugia are abundant, with more widespread lineages distributed across the more homogeneous, low elevation landscape of the central and northern areas. Deep mtDNA genetic divergences, mito-nuclear discordance, and patterns of morphological stasis vs. variation in European *Buthus* reflect broad evolutionary processes that shaped the region’s biodiversity, including isolation in glacial refugia followed by hybridization upon secondary contact and/or ILS. Future studies should expand the sampling along potential contact zones to better understand the role of hybridization and putative ILS in shaping the morphological and genetic structure of *Buthus* in the Iberian Peninsula, as well as to minimize potential biases in tree topology that may have arisen from uneven sampling.

## Supplementary Information

Supplementary Material 1.

## Data Availability

The datasets generated and/or analysed during the current study are available in the Genbank repository [https://www.ncbi.nlm.nih.gov/genbank]; see Table S2 for accession codes).
