# Supplementary material for: Mito-nuclear discordance reveals introgressive hybridization following vicariance and secondary contact in Iberian scorpions (Buthidae: Buthus)
Source: BMC Ecol Evol. 2025 Oct 23;25:112. doi: 10.1186/s12862-025-02445-0 (PMC12548245; doi:10.1186/s12862-025-02445-0)
Supplement: Supplementary file 1 — Supplementary Material 1. [file 12862_2025_2445_MOESM1_ESM.docx]

**SUPPORTING INFORMATION**

Mito-nuclear discordance reveals

introgressive hybridization after vicariance and secondary

contact in Iberian scorpions (Buthidae: *Buthus*)

Javier Blasco-Aróstegui^1, 2,*^ | Yuri Simone^3^ | Octávio S. Paulo^1^ | Lorenzo Prendini^2^

^1^*Centre for Ecology, Evolution and Environmental Changes, Departamento de Biologia Animal,*

*Faculdade de Ciências, Universidade de Lisboa, Campo Grande 016, 1749-016 Lisbon, Portugal;*

email: [javierblasco77@gmail.com](mailto:javierblasco77@gmail.com)

email: [ofpaulo@fc.ul.pt](mailto:ofpaulo@fc.ul.pt)

^2^*Arachnology Lab and Scorpion Systematics Research Group, Division of Invertebrate Zoology,*

*American Museum of Natural History;* email: [lorenzo@amnh.org](mailto:lorenzo@amnh.org)

^3^*Laboratory of Functional Morphology, Department of Biology, University of Antwerp, Wilrijk, Belgium*

**CONTENTS**

**Supplementary Table S1 ………………………………………………………………………... 2**

**Supplementary Table S2 …………………………………………………..………………….... 3**

**Supplementary Table S3 ……………………………………………………..…………….…... 5**

**Supplementary Table S4 ……………………………………………………………………...... 6**

**Supplementary Table S5 ………………………………………………………………..………. 7**

**Supplementary Table S6 ………………………………………………………………….…..... 8**

**Supplementary Table S7 …………………………………………………..………………..… 10**

**Supplementary Table S8 ……………………………………………….……………………... 18**

**Supplementary Table S9 ……………………………………………………...…………….… 19**

**Supplementary Table S10 ………………………………………………….………………… 20**

**Supplementary Table S11 ………………………………………………….………………… 22**

**Supplementary Figure S1 ………………………………………………...………………..… 23**

**Supplementary Figure S2 ……………………………………………...…..………………… 24**

**Supplementary Figure S3 …………………………………………...………..……………… 25**

**Supplementary References …………………………………………………….................. 26**

**Supplementary Tables**

**Table S1.** Primers used to generate DNA sequences from the 18S rDNA (18S), Internal Transcribed Spacer (ITSII), 28S rDNA (28S), 12S rDNA (12S), 16S rDNA (16S), and Cytochrome *c* Oxidase Subunit I (COI) loci for phylogenetic analysis of the European species of *Buthus* Leach, 1815.

| **Locus** | **Primer** | **Sequence** | **Reference** |
| --- | --- | --- | --- |
| 18S | 18S1F | TACCTGGTTGATCCTGCCAGTAG | Giribet et al. [1] |
|  | 18S5R | CTTGGCAAATGCTTTCGC | Giribet et al. [1] |
|  | 18S3F | GTTCGATTCCGGAGAGGGA | Giribet et al. [1] |
|  | 18Sbi | GAGTCTCGTTCGTTATCGGA | Wheeler et al. [2] |
|  | 18Sa2.0 | ATGGTTGCAAAGCTGAAAC | Wheeler et al. [2] |
|  | 18S9R | GATCCTTCCGCAGGTTCACCTAC | Giribet et al. [1] |
| 28S | 28SA | GACCCGTCTTGAAGCACG | Nunn et al. [3] |
|  | 28SBout | CCCACAGCGCCAGTTCTGCTTACC | Prendini & Wheeler [4] |
| ITSII | CAS18sF1 | TACACACCGCCCGTCGCTACTA | Ji et al. [5] |
|  | CAS5p8sB1d | ATGTGCGTTCRAAATGTCGATGTTCA | Ji et al. [5] |
|  | CAS5p8dFc | TGAACATCGACATTTYGAACGCACAT | Ji et al. [5] |
|  | CAS28sB1d-spid | TTCTTTTCCTCCGCTTATTTATATGCTTAA | Ji et al. [5] |
| 12S | 12Sai | AAACTAGGATTAGATACCCTATTAT | Kocher et al. [6] |
|  | 12Sbi | AAGAGCGACGGGCGATGTGT | Kocher et al. [6] |
| 16S | 16Sa | GTGCAAAGGTAGCATAATCA | Gantenbein et al. [7] |
|  | 16SB | CGCCTGTTTATCAAAAACAT | Simon et al. [8] |
| COI | LCO | GGTCAACAAATCATAAAGATATTGG | Folmer et al. [9] |
|  | HCO | TAAACTTCAGGGTGACCAAAAAATCA | Folmer et al. [9] |
|  | COImodF | ATCATAAGGATATTGGGACTATGT | Bryson et al. [10] |
|  | HCOoutout | GTAAATATATGRTGDGCTC | Prendini et al. [11] |
|  | CI-J01718 | GGNGGATTTGGAAATTGRTTRGTTCC | Simon et al. [8] |
|  | CI-N02776 | GGATAATCAGAATANCGNCGAGG | Simon et al. [8] |
|  | EXTA | GAAGTTTATATTTTAATTTTACCTGG | Simon et al. [8] |
|  | EXTB | CCTATTGAWARAACATARTGAAAATG | Simon et al. [8] |
|  | CruzR | CATACCCAAAGARCCAAAAGG | Valdez-Cruz et al. [12] |
|  | Nancy | CCCGGTAAAATTAAAATATAAACTTC | Harrison et al. [13] |

**Table S2.** Tissue samples and GenBank accession codes for DNA sequences of the 18S rDNA (18S), 28S rDNA, Internal Transcribed Spacer (ITSII), 12S rDNA (12S), 16S rDNA (16S) and Cytochrome *c* Oxidase Subunit I (COI) loci used for phylogenetic analyses and hybridization network reconstruction of the European species of *Buthus* Leach, 1815 and three African outgroup species. Classification follows Blasco-Aróstegui et al. (in press).

| **Species** | **AMCC** | **Country** | **Georeference** | **18S** | **ITSII** | **28S** | **12S** | **16S** | **COI** |
| --- | --- | --- | --- | --- | --- | --- | --- | --- | --- |
| **Outgroup** |  |  |  |  |  |  |  |  |  |
| *Buthus elizabethae* | LP 4601 | Senegal | 14°23'22.9"N 12°21'30.9"W | PV828518 | PV828311 | PV828572 | PV828203 | PV828257 | PV831780 |
| *Buthus maroccanus* | LP 16678 | Morocco | 33°58'36.5"N 06°48'05.5"W | PV828519 | PV828312 | PV828573 | PV828204 | PV828258 | PV831781 |
| *Buthus tunetanus* | LP 14847 | Tunisia | 33°35'46.6"N 07°37'26.3"E | PV828520 | PV828313 | PV828574 | PV828205 | PV828259 | PV831782 |
| **Ingroup** |  |  |  |  |  |  |  |  |  |
| *Buthus delafuentei* | LP 6092 | Portugal | 37°34'41.9"N 07°32'01.9"W | PV828479 | PV828272 | PV828533 | PV828164 | PV828218 | PV831741 |
|  | LP 19804 | Spain | 37°29'09"N 05°37'30"W | PV828478 | PV828271 | PV828532 | PV828163 | PV828217 | PV831740 |
|  | LP 19809 |  | 37°06'18.9"N 06°44'13.6"W | PV828476 | PV828269 | PV828530 | PV828161 | PV828215 | PV831738 |
|  | LP 19816 |  | 37°16'52.5"N 06°24'02.3"W | PV828477 | PV828270 | PV828531 | PV828162 | PV828216 | PV831739 |
|  | LP 20690 |  | 36°59'48.3"N 06°31'09.3"W | PV828489 | PV828282 | PV828543 | PV828174 | PV828228 | PV831751 |
| *Buthus elongatus* | LP 19791 | Spain | 36°34'34.1"N 04°52'58.2"W | PV828483 | PV828276 | PV828537 | PV828168 | PV828222 | PV831745 |
| *Buthus garcialorcai* | LP 19780 | Spain | 37°14'09.2"N 03°32'02.7"W | PV828488 | PV828281 | PV828542 | PV828173 | PV828227 | PV831750 |
| *Buthus halius* | LP 6946 | Portugal | 37°22'11"N 08°21'51.8"W | PV828486 | PV828279 | PV828540 | PV828171 | PV828225 | PV831748 |
|  | LP 6947 |  | 37°14'37.1"N 08°07'03.6"W | PV828487 | PV828280 | PV828541 | PV828172 | PV828226 | PV831749 |
|  | LP 18040 |  | 41°16'54.4"N 07°23'44.8"W | PV828490 | PV828283 | PV828544 | PV828175 | PV828229 | PV831752 |
|  | LP 18047 |  | 40°53'21.1"N 08°08'04.3"W | PV828491 | PV828284 | PV828545 | PV828176 | PV828230 | PV831753 |
|  | LP 18063 |  | 37°01'24.3"N 08°59'35.6"W | PV828484 | PV828277 | PV828538 | PV828169 | PV828223 | PV831746 |
|  | LP 18069 |  | 37°04'57.4"N 08°40'17.5"W | PV828485 | PV828278 | PV828539 | PV828170 | PV828224 | PV831747 |
|  | LP 19507 |  | 40°23'08.9"N 07°32'47"W | PV828498 | PV828291 | PV828552 | PV828183 | PV828237 | PV831760 |
|  | LP 19515 |  | 40°22'21.5"N 07°32'57.5"W | PV828499 | PV828292 | PV828553 | PV828184 | PV828238 | PV831761 |
|  | LP 19488 | Spain | 39°19'42.4"N 03°29'28.8"W | PV828470 | PV828263 | PV828524 | PV828155 | PV828209 | PV831732 |
|  | LP 19493 |  | 39°08'13.4"N 04°07'40"W | PV828471 | PV828264 | PV828525 | PV828156 | PV828210 | PV831733 |
|  | LP 19498 |  | 41°33'28.2"N 04°38'25"W | PV828473 | PV828266 | PV828527 | PV828158 | PV828212 | PV831735 |
|  | LP 19504 |  | 41°32'31"N 04°48'33"W | PV828474 | PV828267 | PV828528 | PV828159 | PV828213 | PV831736 |
|  | LP 19817 |  | 40°45'47.2"N 03°59'59.8"W | PV828475 | PV828268 | PV828529 | PV828160 | PV828214 | PV831737 |

**Table S2.** Continued.

| **Species** | **AMCC** | **Country** | **Georeference** | **18S** | **ITSII** | **28S** | **12S** | **16S** | **COI** |
| --- | --- | --- | --- | --- | --- | --- | --- | --- | --- |
| **Ingroup** |  |  |  |  |  |  |  |  |  |
| *Buthus iaspis* | LP 12387 | Spain | 36°48'44"N 02°03'26"E | PV828494 | PV828285 | PV828548 | PV828179 | PV828233 | PV831756 |
|  | LP 19756 |  | 36°44'02.8"N 02°08'35"W | PV828492 | PV828286 | PV828546 | PV828177 | PV828231 | PV831754 |
|  | LP 19760 |  | 36°49'41.3"N 02°15'47.2"W | PV828493 | PV828287 | PV828547 | PV828178 | PV828232 | PV831755 |
|  | LP 19764 |  | 37°05'06.7"N 02°18'12.3"W | PV828495 | PV828288 | PV828549 | PV828180 | PV828234 | PV831757 |
| *Buthus ibericus* | LP 19784 | Spain | 36°42'26.4"N 07°32'01.9"W | PV828496 | PV828289 | PV828550 | PV828181 | PV828235 | PV831758 |
|  | LP 19795 |  | 36°37'40.3"N 05°39'52.5"W | PV828497 | PV828290 | PV828551 | PV828182 | PV828236 | PV831759 |
|  | LP 19801 |  | 37°04'46.9"N 05°30'49.3"W | PV828472 | PV828265 | PV828526 | PV828183 | PV828211 | PV831762 |
| *Buthus manchego* | LP 19519 | Spain | 38°55'08.7"N 02°43'35.4"W | PV828500 | PV828293 | PV828554 | PV828185 | PV828239 | PV831763 |
|  | LP 19524 |  | 38°55'08.7"N 02°43'35.4"W | PV828501 | PV828294 | PV828555 | PV828186 | PV828240 | PV831764 |
|  | LP 19533 |  | 40°17'53.6"N 02°08'14.3"W | PV828514 | PV828307 | PV828568 | PV828199 | PV828255 | PV831776 |
|  | LP 19537 |  | 40°01'44.3"N 01°57'59.4"W | PV828515 | PV828308 | PV828569 | PV828200 | PV828256 | PV831777 |
|  | LP 19740 |  | 38°38'27.4"N 00°51'36.8"W | PV828467 | PV828260 | PV828521 | PV828152 | PV828206 | PV831729 |
|  | LP 19744 |  | 38°25'34.5"N 00°31'41.7"W | PV828469 | PV828262 | PV828523 | PV828154 | PV828208 | PV831731 |
|  | LP 19748 |  | 38°16'57"N 00°31'30.7"W | PV828468 | PV828261 | PV828522 | PV828153 | PV828207 | PV831730 |
|  | LP 19750 |  | 37°48'52"N 01°34'49.2"W | PV828517 | PV828309 | PV828571 | PV828202 | PV828254 | PV831779 |
|  | LP 19767 |  | 37°42'51.2"N 02°30'06.3"W | PV828516 | PV828310 | PV828570 | PV828201 | PV828253 | PV831778 |
| *Buthus montanus* | LP 19769 | Spain | 37°06'35.5"N 03°01'23.4"W | PV828502 | PV828295 | PV828556 | PV828187 | PV828241 | PV831764 |
|  | LP 19775 |  | 37°16'46.1"N 03°15'11.7"W | PV828503 | PV828296 | PV828557 | PV828188 | PV828242 | PV831765 |
| *Buthus occitanus* | LP 16670 | France | 43°48'53.4"N 03°44'19.9"E | PV828504 | PV828297 | PV828558 | PV828189 | PV828243 | PV831766 |
|  | LP 16671 |  | 43°53'19.2"N 03°50'10.5"E | PV828505 | PV828298 | PV828559 | PV828190 | PV828244 | PV831767 |
|  | LP 19715 |  | 43°19'38.9"N 05°46'12.1"E | PV828512 | PV828303 | PV828566 | PV828197 | PV828251 | PV831774 |
|  | LP 19717 |  | 43°48'34.8"N 04°08'44"E | PV828510 | PV828304 | PV828564 | PV828195 | PV828249 | PV831772 |
|  | LP 19725 |  | 42°36'46.8"N 02°35'24.9"E | PV828513 | PV828305 | PV828567 | PV828198 | PV828252 | PV831775 |
|  | LP 17847 | Spain | 42°03'06.8"N 01°42'16.4"E | PV828506 | PV828299 | PV828560 | PV828191 | PV828245 | PV831768 |
|  | LP 17972 |  | 42°00'01.4"N 01°22'17.8"E | PV828507 | PV828300 | PV828561 | PV828192 | PV828246 | PV831769 |
|  | LP 18488 |  | 42°00'01.4"N 01°22'17.8"E | PV828508 | PV828301 | PV828562 | PV828193 | PV828247 | PV831770 |
|  | LP 19529 |  | 42°35'03.6"N 01°14'03.3"W | PV828509 | PV828302 | PV828563 | PV828194 | PV828248 | PV831771 |
|  | LP 19727 |  | 41°16'38.4"N 01°52'27.8"E | PV828511 | PV828306 | PV828565 | PV828196 | PV828250 | PV831773 |
|  | LP 19734 |  | 39°41'55.3"N 00°24'38"W | PV828480 | PV828274 | PV828534 | PV828165 | PV828219 | PV831742 |
|  | LP 20004 |  | 39°53'53.7"N 00°41'06.9"E | PV828481 | PV828273 | PV828535 | PV828166 | PV828220 | PV831743 |
|  | LP 20655 |  | 40°06'42.6"N 00°43'53.4"W | PV828482 | PV828275 | PV828536 | PV828167 | PV828221 | PV831744 |

**Table S3.** Pairwise genetic distances for the nuclear Internal Transcribed Spacer II locus among putative species of *Buthus* Leach, 1815 scorpions in the Iberian Peninsula and Southeastern France. **A**, *B. ajax* (C.L. Koch, 1839); **B,** *B. alacanti* Teruel & Turiel, 2020; **C,** *B. baeticus* Teruel & Turiel, 2020; **D,** *B. balmensis* Ythier & Laborieux, 2022; **E,** *B. castellano* Teruel & Turiel, 2022; **F,** *B. delafuentei* Teruel & Turiel, 2020; **G,** *B. elongatus* Rossi, 2012; **H,** *B. gabani* Ythier, 2021; **I,** *B. garcualorcai* Teruel & Turiel, 2020; **J,** *B. gonzalezdelavegai* González-Moliné & Armas, 2024; **K,** *B. halius* (C.L. Koch, 1839); **L,** *B. iaspis* Teruel & Turiel, 2020; **M,** *B. ibericus* Lourenço & Vachon, 2004; **N,** *B. lusitanus* Lourenço, 2021; **O,** *B. manchego* Teruel & Turiel, 2020; **P,** *B. montanus* Lourenço & Vachon, 2004; **Q,** *B.* aff. *occitanus*; **R,** *B. occitanus* (Amoreux, 1789); **S,** *B. pedrosousai* Teruel & Turiel, 2021; **T,** *B. pyrenaeus* Ythier, 2021; **U,** *B. serrano* Teruel & Turiel, 2020; **V,** *B. tunetanus* (Herbst, 1800).

|  |  | **A** | **B** | **C** | **D** | **E** | **F** | **G** | **H** | **I** | **J** | **K** | **L** | **M** | **N** | **O** | **P** | **Q** | **R** | **S** | **T** | **U** | **V** |
| --- | --- | --- | --- | --- | --- | --- | --- | --- | --- | --- | --- | --- | --- | --- | --- | --- | --- | --- | --- | --- | --- | --- | --- |
| **ITSII** | **A** | **0.000** |  |  |  |  |  |  |  |  |  |  |  |  |  |  |  |  |  |  |  |  |  |
|  | **B** | 0.040 | **0.026** |  |  |  |  |  |  |  |  |  |  |  |  |  |  |  |  |  |  |  |  |
|  | **C** | 0.007 | 0.031 | **-** |  |  |  |  |  |  |  |  |  |  |  |  |  |  |  |  |  |  |  |
|  | **D** | 0.040 | 0.022 | 0.030 | **-** |  |  |  |  |  |  |  |  |  |  |  |  |  |  |  |  |  |  |
|  | **E** | 0.040 | 0.015 | 0.030 | 0.022 | **0.000** |  |  |  |  |  |  |  |  |  |  |  |  |  |  |  |  |  |
|  | **F** | 0.006 | 0.035 | 0.004 | 0.035 | 0.035 | **0.004** |  |  |  |  |  |  |  |  |  |  |  |  |  |  |  |  |
|  | **G** | 0.037 | 0.038 | 0.030 | 0.031 | 0.039 | 0.032 | **-** |  |  |  |  |  |  |  |  |  |  |  |  |  |  |  |
|  | **H** | 0.011 | 0.037 | 0.006 | 0.036 | 0.036 | 0.006 | 0.032 | **0.001** |  |  |  |  |  |  |  |  |  |  |  |  |  |  |
|  | **I** | 0.042 | 0.039 | 0.042 | 0.046 | 0.029 | 0.040 | 0.055 | 0.046 | **-** |  |  |  |  |  |  |  |  |  |  |  |  |  |
|  | **J** | 0.005 | 0.032 | 0.002 | 0.032 | 0.032 | 0.002 | 0.030 | 0.006 | 0.040 | **-** |  |  |  |  |  |  |  |  |  |  |  |  |
|  | **K** | 0.042 | 0.017 | 0.032 | 0.024 | 0.002 | 0.037 | 0.041 | 0.039 | 0.031 | 0.035 | **0.000** |  |  |  |  |  |  |  |  |  |  |  |
|  | **L** | 0.037 | 0.026 | 0.032 | 0.009 | 0.030 | 0.033 | 0.028 | 0.036 | 0.050 | 0.030 | 0.033 | **0.004** |  |  |  |  |  |  |  |  |  |  |
|  | **M** | 0.041 | 0.034 | 0.033 | 0.028 | 0.035 | 0.035 | 0.005 | 0.035 | 0.052 | 0.033 | 0.038 | 0.025 | **0.002** |  |  |  |  |  |  |  |  |  |
|  | **N** | 0.007 | 0.035 | 0.005 | 0.035 | 0.035 | 0.004 | 0.032 | 0.008 | 0.042 | 0.002 | 0.037 | 0.032 | 0.036 | **0.000** |  |  |  |  |  |  |  |  |
|  | **O** | 0.040 | 0.015 | 0.030 | 0.029 | 0.005 | 0.035 | 0.041 | 0.036 | 0.031 | 0.032 | 0.007 | 0.037 | 0.037 | 0.035 | **0.000** |  |  |  |  |  |  |  |
|  | **P** | 0.035 | 0.023 | 0.030 | 0.006 | 0.028 | 0.030 | 0.029 | 0.034 | 0.047 | 0.027 | 0.030 | 0.003 | 0.027 | 0.030 | 0.035 | **0.000** |  |  |  |  |  |  |
|  | **Q** | 0.040 | 0.017 | 0.031 | 0.022 | 0.010 | 0.035 | 0.039 | 0.037 | 0.036 | 0.033 | 0.012 | 0.029 | 0.035 | 0.036 | 0.009 | 0.026 | **0.017** |  |  |  |  |  |
|  | **R** | 0.040 | 0.022 | 0.030 | 0.000 | 0.022 | 0.035 | 0.031 | 0.037 | 0.046 | 0.032 | 0.024 | 0.009 | 0.028 | 0.035 | 0.029 | 0.006 | 0.022 | **0.000** |  |  |  |  |
|  | **S** | 0.040 | 0.016 | 0.030 | 0.027 | 0.002 | 0.035 | 0.041 | 0.036 | 0.031 | 0.032 | 0.005 | 0.035 | 0.037 | 0.035 | 0.002 | 0.033 | 0.010 | 0.027 | **0.005** |  |  |  |
|  | **T** | 0.040 | 0.022 | 0.030 | 0.000 | 0.022 | 0.035 | 0.031 | 0.036 | 0.046 | 0.032 | 0.024 | 0.009 | 0.028 | 0.035 | 0.029 | 0.006 | 0.022 | 0.000 | 0.027 | **-** |  |  |
|  | **U** | 0.040 | 0.015 | 0.030 | 0.029 | 0.005 | 0.035 | 0.041 | 0.036 | 0.031 | 0.032 | 0.007 | 0.037 | 0.037 | 0.035 | 0.000 | 0.035 | 0.009 | 0.029 | 0.002 | 0.029 | **0.000** |  |
|  | **V** | 0.082 | 0.084 | 0.077 | 0.087 | 0.082 | 0.077 | 0.082 | 0.077 | 0.090 | 0.077 | 0.084 | 0.086 | 0.081 | 0.077 | 0.082 | 0.085 | 0.084 | 0.087 | 0.082 | 0.087 | 0.082 | **-** |

**Table S4.** Pairwise genetic distances for the mitochondrial 12S rRNA locus among putative species of *Buthus* Leach, 1815 scorpions in the Iberian Peninsula and Southeastern France. **A**, *B. ajax* (C.L. Koch, 1839); **B,** *B. alacanti* Teruel & Turiel, 2020; **C,** *B. baeticus* Teruel & Turiel, 2020; **D,** *B. balmensis* Ythier & Laborieux, 2022; **E,** *B. castellano* Teruel & Turiel, 2022; **F,** *B. delafuentei* Teruel & Turiel, 2020; **G,** *B. elongatus* Rossi, 2012; **H,** *B. gabani* Ythier, 2021; **I,** *B. garcualorcai* Teruel & Turiel, 2020; **J,** *B. gonzalezdelavegai* González-Moliné & Armas, 2024; **K,** *B. halius* (C.L. Koch, 1839); **L,** *B. iaspis* Teruel & Turiel, 2020; **M,** *B. ibericus* Lourenço & Vachon, 2004; **N,** *B. lusitanus* Lourenço, 2021; **O,** *B. manchego* Teruel & Turiel, 2020; **P,** *B. montanus* Lourenço & Vachon, 2004; **Q,** *B.* aff. *occitanus*; **R,** *B. occitanus* (Amoreux, 1789); **S,** *B. pedrosousai* Teruel & Turiel, 2021; **T,** *B. pyrenaeus* Ythier, 2021; **U,** *B. serrano* Teruel & Turiel, 2020; **V,** *B. tunetanus* (Herbst, 1800).

|  |  | **A** | **B** | **C** | **D** | **E** | **F** | **G** | **H** | **I** | **J** | **K** | **L** | **M** | **N** | **O** | **P** | **Q** | **R** | **S** | **T** | **U** | **V** |
| --- | --- | --- | --- | --- | --- | --- | --- | --- | --- | --- | --- | --- | --- | --- | --- | --- | --- | --- | --- | --- | --- | --- | --- |
| **12S** | **A** | **0.000** |  |  |  |  |  |  |  |  |  |  |  |  |  |  |  |  |  |  |  |  |  |
|  | **B** | 0.104 | **0.024** |  |  |  |  |  |  |  |  |  |  |  |  |  |  |  |  |  |  |  |  |
|  | **C** | 0.095 | 0.100 | **-** |  |  |  |  |  |  |  |  |  |  |  |  |  |  |  |  |  |  |  |
|  | **D** | 0.089 | 0.092 | 0.098 | **-** |  |  |  |  |  |  |  |  |  |  |  |  |  |  |  |  |  |  |
|  | **E** | 0.014 | 0.100 | 0.093 | 0.081 | **0.004** |  |  |  |  |  |  |  |  |  |  |  |  |  |  |  |  |  |
|  | **F** | 0.029 | 0.099 | 0.095 | 0.083 | 0.037 | **0.023** |  |  |  |  |  |  |  |  |  |  |  |  |  |  |  |  |
|  | **G** | 0.092 | 0.089 | 0.098 | 0.089 | 0.084 | 0.095 | **-** |  |  |  |  |  |  |  |  |  |  |  |  |  |  |  |
|  | **H** | 0.027 | 0.103 | 0.092 | 0.089 | 0.033 | 0.036 | 0.096 | **0.008** |  |  |  |  |  |  |  |  |  |  |  |  |  |  |
|  | **I** | 0.107 | 0.089 | 0.098 | 0.092 | 0.105 | 0.107 | 0.074 | 0.097 | **-** |  |  |  |  |  |  |  |  |  |  |  |  |  |
|  | **J** | 0.021 | 0.097 | 0.089 | 0.083 | 0.029 | 0.016 | 0.092 | 0.030 | 0.107 | **-** |  |  |  |  |  |  |  |  |  |  |  |  |
|  | **K** | 0.040 | 0.101 | 0.095 | 0.077 | 0.042 | 0.041 | 0.083 | 0.047 | 0.104 | 0.034 | **0.009** |  |  |  |  |  |  |  |  |  |  |  |
|  | **L** | 0.090 | 0.122 | 0.131 | 0.102 | 0.098 | 0.093 | 0.107 | 0.099 | 0.095 | 0.093 | 0.105 | **0.008** |  |  |  |  |  |  |  |  |  |  |
|  | **M** | 0.089 | 0.101 | 0.006 | 0.092 | 0.087 | 0.092 | 0.098 | 0.087 | 0.092 | 0.089 | 0.089 | 0.125 | **0.000** |  |  |  |  |  |  |  |  |  |
|  | **N** | 0.006 | 0.098 | 0.095 | 0.089 | 0.020 | 0.035 | 0.086 | 0.033 | 0.101 | 0.027 | 0.040 | 0.093 | 0.089 | **0.000** |  |  |  |  |  |  |  |  |
|  | **O** | 0.113 | 0.026 | 0.098 | 0.101 | 0.109 | 0.108 | 0.095 | 0.112 | 0.092 | 0.107 | 0.110 | 0.131 | 0.098 | 0.107 | **0.000** |  |  |  |  |  |  |  |
|  | **P** | 0.093 | 0.110 | 0.112 | 0.088 | 0.089 | 0.090 | 0.095 | 0.088 | 0.079 | 0.091 | 0.093 | 0.047 | 0.107 | 0.093 | 0.118 | **0.012** |  |  |  |  |  |  |
|  | **Q** | 0.100 | 0.079 | 0.089 | 0.037 | 0.091 | 0.095 | 0.104 | 0.093 | 0.081 | 0.094 | 0.087 | 0.110 | 0.083 | 0.100 | 0.089 | 0.093 | **0.008** |  |  |  |  |  |
|  | **R** | 0.088 | 0.092 | 0.097 | 0.001 | 0.080 | 0.081 | 0.089 | 0.087 | 0.091 | 0.082 | 0.076 | 0.102 | 0.091 | 0.088 | 0.101 | 0.088 | 0.036 | **0.002** |  |  |  |  |
|  | **S** | 0.105 | 0.022 | 0.096 | 0.093 | 0.101 | 0.100 | 0.087 | 0.105 | 0.091 | 0.099 | 0.102 | 0.123 | 0.096 | 0.099 | 0.010 | 0.111 | 0.082 | 0.094 | **0.003** |  |  |  |
|  | **T** | 0.090 | 0.093 | 0.099 | 0.000 | 0.082 | 0.083 | 0.090 | 0.089 | 0.093 | 0.084 | 0.078 | 0.103 | 0.093 | 0.090 | 0.101 | 0.088 | 0.038 | 0.001 | 0.094 | **-** |  |  |
|  | **U** | 0.111 | 0.028 | 0.102 | 0.096 | 0.107 | 0.105 | 0.093 | 0.111 | 0.091 | 0.105 | 0.108 | 0.120 | 0.102 | 0.105 | 0.019 | 0.111 | 0.084 | 0.097 | 0.021 | 0.097 | **0.003** |  |
|  | **V** | 0.113 | 0.136 | 0.119 | 0.116 | 0.103 | 0.110 | 0.125 | 0.122 | 0.146 | 0.110 | 0.110 | 0.142 | 0.113 | 0.113 | 0.136 | 0.135 | 0.124 | 0.115 | 0.129 | 0.117 | 0.135 | **-** |

**Table S5.** Pairwise genetic distances for the mitochondrial 16S rRNA locus putative species of *Buthus* Leach, 1815 scorpions in the Iberian Peninsula and Southeastern France. **A**, *B. ajax* (C.L. Koch, 1839); **B,** *B. alacanti* Teruel & Turiel, 2020; **C,** *B. baeticus* Teruel & Turiel, 2020; **D,** *B. balmensis* Ythier & Laborieux, 2022; **E,** *B. castellano* Teruel & Turiel, 2022; **F,** *B. delafuentei* Teruel & Turiel, 2020; **G,** *B. elongatus* Rossi, 2012; **H,** *B. gabani* Ythier, 2021; **I,** *B. garcualorcai* Teruel & Turiel, 2020; **J,** *B. gonzalezdelavegai* González-Moliné & Armas, 2024; **K,** *B. halius* (C.L. Koch, 1839); **L,** *B. iaspis* Teruel & Turiel, 2020; **M,** *B. ibericus* Lourenço & Vachon, 2004; **N,** *B. lusitanus* Lourenço, 2021; **O,** *B. manchego* Teruel & Turiel, 2020; **P,** *B. montanus* Lourenço & Vachon, 2004; **Q,** *B.* aff. *occitanus*; **R,** *B. occitanus* (Amoreux, 1789); **S,** *B. pedrosousai* Teruel & Turiel, 2021; **T,** *B. pyrenaeus* Ythier, 2021; **U,** *B. serrano* Teruel & Turiel, 2020; **V,** *B. tunetanus* (Herbst, 1800).

|  |  | **A** | **B** | **C** | **D** | **E** | **F** | **G** | **H** | **I** | **J** | **K** | **L** | **M** | **N** | **O** | **P** | **Q** | **R** | **S** | **T** | **U** | **V** |
| --- | --- | --- | --- | --- | --- | --- | --- | --- | --- | --- | --- | --- | --- | --- | --- | --- | --- | --- | --- | --- | --- | --- | --- |
| 16S | **A** | **0.000** |  |  |  |  |  |  |  |  |  |  |  |  |  |  |  |  |  |  |  |  |  |
|  | **B** | 0.093 | **0.026** |  |  |  |  |  |  |  |  |  |  |  |  |  |  |  |  |  |  |  |  |
|  | **C** | 0.095 | 0.117 | **-** |  |  |  |  |  |  |  |  |  |  |  |  |  |  |  |  |  |  |  |
|  | **D** | 0.098 | 0.101 | 0.108 | **-** |  |  |  |  |  |  |  |  |  |  |  |  |  |  |  |  |  |  |
|  | **E** | 0.032 | 0.110 | 0.100 | 0.099 | **0.010** |  |  |  |  |  |  |  |  |  |  |  |  |  |  |  |  |  |
|  | **F** | 0.030 | 0.090 | 0.094 | 0.091 | 0.042 | **0.017** |  |  |  |  |  |  |  |  |  |  |  |  |  |  |  |  |
|  | **G** | 0.106 | 0.101 | 0.103 | 0.095 | 0.107 | 0.099 | **-** |  |  |  |  |  |  |  |  |  |  |  |  |  |  |  |
|  | **H** | 0.031 | 0.084 | 0.080 | 0.093 | 0.043 | 0.031 | 0.093 | **0.005** |  |  |  |  |  |  |  |  |  |  |  |  |  |  |
|  | **I** | 0.080 | 0.093 | 0.085 | 0.090 | 0.084 | 0.074 | 0.087 | 0.074 | **-** |  |  |  |  |  |  |  |  |  |  |  |  |  |
|  | **J** | 0.036 | 0.088 | 0.098 | 0.088 | 0.051 | 0.017 | 0.101 | 0.034 | 0.069 | **-** |  |  |  |  |  |  |  |  |  |  |  |  |
|  | **K** | 0.033 | 0.096 | 0.094 | 0.096 | 0.038 | 0.030 | 0.100 | 0.030 | 0.077 | 0.033 | **0.010** |  |  |  |  |  |  |  |  |  |  |  |
|  | **L** | 0.083 | 0.113 | 0.094 | 0.104 | 0.078 | 0.081 | 0.109 | 0.079 | 0.077 | 0.084 | 0.085 | **0.005** |  |  |  |  |  |  |  |  |  |  |
|  | **M** | 0.085 | 0.109 | 0.022 | 0.102 | 0.093 | 0.081 | 0.101 | 0.066 | 0.087 | 0.085 | 0.081 | 0.088 | **0.021** |  |  |  |  |  |  |  |  |  |
|  | **N** | 0.017 | 0.098 | 0.089 | 0.091 | 0.026 | 0.031 | 0.099 | 0.030 | 0.076 | 0.037 | 0.027 | 0.077 | 0.079 | **0.003** |  |  |  |  |  |  |  |  |
|  | **O** | 0.085 | 0.027 | 0.118 | 0.093 | 0.104 | 0.091 | 0.095 | 0.085 | 0.095 | 0.090 | 0.098 | 0.114 | 0.110 | 0.091 | **0.000** |  |  |  |  |  |  |  |
|  | **P** | 0.084 | 0.104 | 0.094 | 0.107 | 0.091 | 0.081 | 0.099 | 0.075 | 0.068 | 0.078 | 0.087 | 0.042 | 0.087 | 0.082 | 0.108 | **0.013** |  |  |  |  |  |  |
|  | **Q** | 0.088 | 0.098 | 0.108 | 0.054 | 0.095 | 0.089 | 0.104 | 0.079 | 0.089 | 0.089 | 0.083 | 0.102 | 0.095 | 0.080 | 0.097 | 0.094 | **0.009** |  |  |  |  |  |
|  | **R** | 0.099 | 0.107 | 0.109 | 0.010 | 0.100 | 0.098 | 0.098 | 0.094 | 0.091 | 0.094 | 0.098 | 0.105 | 0.103 | 0.093 | 0.101 | 0.108 | 0.053 | **0.009** |  |  |  |  |
|  | **S** | 0.087 | 0.032 | 0.123 | 0.095 | 0.106 | 0.094 | 0.101 | 0.087 | 0.098 | 0.093 | 0.100 | 0.117 | 0.112 | 0.094 | 0.008 | 0.111 | 0.100 | 0.102 | **0.005** |  |  |  |
|  | **T** | 0.095 | 0.102 | 0.105 | 0.003 | 0.096 | 0.094 | 0.098 | 0.091 | 0.087 | 0.090 | 0.094 | 0.102 | 0.099 | 0.089 | 0.095 | 0.104 | 0.052 | 0.007 | 0.098 | **-** |  |  |
|  | **U** | 0.080 | 0.032 | 0.112 | 0.085 | 0.100 | 0.087 | 0.097 | 0.078 | 0.090 | 0.085 | 0.094 | 0.111 | 0.102 | 0.089 | 0.021 | 0.102 | 0.090 | 0.094 | 0.024 | 0.088 | **0.010** |  |
|  | **V** | 0.127 | 0.120 | 0.090 | 0.121 | 0.135 | 0.115 | 0.131 | 0.117 | 0.101 | 0.114 | 0.122 | 0.091 | 0.096 | 0.125 | 0.116 | 0.104 | 0.129 | 0.128 | 0.121 | 0.124 | 0.115 | **-** |

**Table S6.** Pairwise genetic distances for the mitochondrial Cytochrome *c* Oxidase Subunit I locus putative species of *Buthus* Leach, 1815 scorpions in the Iberian Peninsula and Southeastern France. **A**, *B. ajax* (C.L. Koch, 1839); **B,** *B. alacanti* Teruel & Turiel, 2020; **C,** *B. baeticus* Teruel & Turiel, 2020; **D,** *B. balmensis* Ythier & Laborieux, 2022; **E,** *B. castellano* Teruel & Turiel, 2022; **F,** *B. delafuentei* Teruel & Turiel, 2020; **G,** *B. elongatus* Rossi, 2012; **H,** *B. gabani* Ythier, 2021; **I,** *B. garcualorcai* Teruel & Turiel, 2020; **J,** *B. gonzalezdelavegai* González-Moliné & Armas, 2024; **K,** *B. halius* (C.L. Koch, 1839); **L,** *B. iaspis* Teruel & Turiel, 2020; **M,** *B. ibericus* Lourenço & Vachon, 2004; **N,** *B. lusitanus* Lourenço, 2021; **O,** *B. manchego* Teruel & Turiel, 2020; **P,** *B. montanus* Lourenço & Vachon, 2004; **Q,** *B.* aff. *occitanus*; **R,** *B. occitanus* (Amoreux, 1789); **S,** *B. pedrosousai* Teruel & Turiel, 2021; **T,** *B. pyrenaeus* Ythier, 2021; **U,** *B. serrano* Teruel & Turiel, 2020; **V,** *B. tunetanus* (Herbst, 1800).

|  |  | **A** | **B** | **C** | **D** | **E** | **F** | **G** | **H** | **I** | **J** | **K** | **L** | **M** | **N** | **O** | **P** | **Q** | **R** | **S** | **T** | **U** | **V** |
| --- | --- | --- | --- | --- | --- | --- | --- | --- | --- | --- | --- | --- | --- | --- | --- | --- | --- | --- | --- | --- | --- | --- | --- |
| COI | **A** | **0.001** |  |  |  |  |  |  |  |  |  |  |  |  |  |  |  |  |  |  |  |  |  |
|  | **B** | 0.106 | **0.025** |  |  |  |  |  |  |  |  |  |  |  |  |  |  |  |  |  |  |  |  |
|  | **C** | 0.081 | 0.103 | **-** |  |  |  |  |  |  |  |  |  |  |  |  |  |  |  |  |  |  |  |
|  | **D** | 0.094 | 0.113 | 0.099 | **-** |  |  |  |  |  |  |  |  |  |  |  |  |  |  |  |  |  |  |
|  | **E** | 0.023 | 0.103 | 0.078 | 0.090 | **0.001** |  |  |  |  |  |  |  |  |  |  |  |  |  |  |  |  |  |
|  | **F** | 0.043 | 0.100 | 0.080 | 0.095 | 0.050 | **0.024** |  |  |  |  |  |  |  |  |  |  |  |  |  |  |  |  |
|  | **G** | 0.094 | 0.094 | 0.074 | 0.093 | 0.089 | 0.094 | **-** |  |  |  |  |  |  |  |  |  |  |  |  |  |  |  |
|  | **H** | 0.050 | 0.113 | 0.092 | 0.093 | 0.052 | 0.052 | 0.105 | **0.010** |  |  |  |  |  |  |  |  |  |  |  |  |  |  |
|  | **I** | 0.084 | 0.097 | 0.077 | 0.087 | 0.088 | 0.084 | 0.085 | 0.088 | **-** |  |  |  |  |  |  |  |  |  |  |  |  |  |
|  | **J** | 0.044 | 0.101 | 0.081 | 0.090 | 0.053 | 0.021 | 0.092 | 0.052 | 0.084 | **-** |  |  |  |  |  |  |  |  |  |  |  |  |
|  | **K** | 0.045 | 0.106 | 0.085 | 0.094 | 0.048 | 0.047 | 0.096 | 0.053 | 0.090 | 0.048 | **0.009** |  |  |  |  |  |  |  |  |  |  |  |
|  | **L** | 0.103 | 0.100 | 0.087 | 0.101 | 0.101 | 0.102 | 0.088 | 0.102 | 0.097 | 0.102 | 0.102 | **0.009** |  |  |  |  |  |  |  |  |  |  |
|  | **M** | 0.082 | 0.103 | 0.023 | 0.094 | 0.077 | 0.080 | 0.077 | 0.093 | 0.078 | 0.083 | 0.085 | 0.087 | **0.007** |  |  |  |  |  |  |  |  |  |
|  | **N** | 0.016 | 0.105 | 0.083 | 0.091 | 0.025 | 0.043 | 0.096 | 0.048 | 0.091 | 0.045 | 0.044 | 0.108 | 0.085 | **0.002** |  |  |  |  |  |  |  |  |
|  | **O** | 0.102 | 0.030 | 0.100 | 0.111 | 0.098 | 0.098 | 0.091 | 0.106 | 0.095 | 0.096 | 0.100 | 0.101 | 0.103 | 0.102 | **0.003** |  |  |  |  |  |  |  |
|  | **P** | 0.092 | 0.093 | 0.098 | 0.105 | 0.092 | 0.101 | 0.094 | 0.099 | 0.091 | 0.103 | 0.100 | 0.083 | 0.096 | 0.095 | 0.094 | **0.019** |  |  |  |  |  |  |
|  | **Q** | 0.089 | 0.100 | 0.096 | 0.049 | 0.092 | 0.094 | 0.092 | 0.099 | 0.088 | 0.091 | 0.093 | 0.098 | 0.095 | 0.090 | 0.100 | 0.100 | **0.015** |  |  |  |  |  |
|  | **R** | 0.093 | 0.110 | 0.099 | 0.005 | 0.089 | 0.093 | 0.092 | 0.091 | 0.084 | 0.088 | 0.093 | 0.100 | 0.093 | 0.090 | 0.109 | 0.104 | 0.050 | **0.004** |  |  |  |  |
|  | **S** | 0.099 | 0.029 | 0.105 | 0.109 | 0.097 | 0.095 | 0.094 | 0.105 | 0.096 | 0.095 | 0.102 | 0.101 | 0.103 | 0.100 | 0.012 | 0.092 | 0.100 | 0.108 | **0.008** |  |  |  |
|  | **T** | 0.092 | 0.111 | 0.097 | 0.002 | 0.088 | 0.093 | 0.091 | 0.091 | 0.085 | 0.088 | 0.092 | 0.099 | 0.092 | 0.089 | 0.109 | 0.103 | 0.047 | 0.003 | 0.108 | **-** |  |  |
|  | **U** | 0.099 | 0.030 | 0.102 | 0.109 | 0.102 | 0.103 | 0.095 | 0.114 | 0.099 | 0.102 | 0.105 | 0.102 | 0.099 | 0.103 | 0.028 | 0.095 | 0.094 | 0.107 | 0.027 | 0.108 | **0.009** |  |
|  | **V** | 0.088 | 0.108 | 0.092 | 0.108 | 0.095 | 0.097 | 0.106 | 0.092 | 0.093 | 0.102 | 0.094 | 0.100 | 0.095 | 0.099 | 0.105 | 0.103 | 0.100 | 0.105 | 0.107 | 0.106 | 0.106 | **-** |

**Table S7.** Georeferenced localities and environmental variables values used in ENM, CCA analyses and boxplots of *Buthus* Leach, 1815 in the Iberian Peninsula amd SE France. Data from field sites, unpublished data and verified records from the literature and online databases.

| **Species** | **x** | **y** | **silt** | **sand** | **ph** | **elevation** | **coarse** | **clay** | **bio15** | **bio14** | **bio12** | **bio4** | **bio3** | **bio2** | **bio1** |
| --- | --- | --- | --- | --- | --- | --- | --- | --- | --- | --- | --- | --- | --- | --- | --- |
| *Buthus delafuentei* | -6.54661 | 37.00808 | 26 | 58 | 71 | 24 | 7 | 16 | 70.48974 | 2 | 503 | 515.2778 | 41.66667 | 10.375 | 17.84583 |
| *Buthus delafuentei* | -6.40065 | 37.28125 | 27 | 58 | 64 | 77 | 15 | 15 | 67.42243 | 2 | 521 | 562.3063 | 41.45377 | 11.35833 | 17.75417 |
| *Buthus delafuentei* | -6.7371 | 37.10525 | 25 | 59 | 67 | 49 | 10 | 16 | 70.29258 | 2 | 490 | 498.5836 | 41.49233 | 9.91667 | 17.60833 |
| *Buthus delafuentei* | -5.625 | 37.48583 | 32 | 40 | 75 | 138 | 15 | 27 | 66.32407 | 2 | 569 | 605.6194 | 42.63889 | 12.79167 | 17.64583 |
| *Buthus delafuentei* | -7.53488 | 37.5758 | 32 | 46 | 64 | 139 | 19 | 22 | 66.23215 | 3 | 532 | 527.2533 | 42.94381 | 11.20833 | 17.1625 |
| *Buthus delafuentei* | -6.62744 | 37.04777 | 27 | 54 | 69 | 55 | 10 | 19 | 70.5523 | 2 | 502 | 516.9907 | 41.76667 | 10.44167 | 17.77083 |
| *Buthus delafuentei* | -6.69273 | 37.07853 | 29 | 53 | 71 | 49 | 11 | 19 | 70.50297 | 2 | 495 | 503.733 | 41.46005 | 10.03333 | 17.66667 |
| *Buthus delafuentei* | -6.69143 | 37.07235 | 29 | 49 | 73 | 29 | 8 | 22 | 69.84701 | 2 | 491 | 501.3231 | 41.42361 | 9.94167 | 17.74583 |
| *Buthus delafuentei* | -6.62638 | 37.04614 | 28 | 54 | 68 | 55 | 10 | 18 | 70.5523 | 2 | 502 | 516.9907 | 41.76667 | 10.44167 | 17.77083 |
| *Buthus delafuentei* | -6.62809 | 37.04189 | 29 | 53 | 70 | 55 | 8 | 18 | 70.5523 | 2 | 502 | 516.9907 | 41.76667 | 10.44167 | 17.77083 |
| *Buthus delafuentei* | -6.55443 | 37.02198 | 27 | 55 | 68 | 32 | 8 | 19 | 69.73547 | 2 | 501 | 520.1937 | 41.93122 | 10.56667 | 17.86667 |
| *Buthus delafuentei* | -6.54889 | 37.0147 | 25 | 54 | 68 | 28 | 12 | 21 | 70.03442 | 2 | 501 | 519.7091 | 41.86587 | 10.50833 | 17.85417 |
| *Buthus delafuentei* | -6.50466 | 36.97141 | 29 | 52 | 73 | 9 | 8 | 19 | 71.23337 | 1 | 516 | 508.7304 | 41.83743 | 10.20833 | 17.82917 |
| *Buthus delafuentei* | -6.45141 | 36.99148 | 30 | 51 | 67 | 9 | 8 | 19 | 70.83266 | 1 | 502 | 513.3142 | 42.19788 | 10.59167 | 17.9375 |
| *Buthus elongatus* | -4.88283 | 36.57615 | 35 | 46 | 63 | 862 | 17 | 19 | 70.03561 | 2 | 740 | 554.3648 | 39.22137 | 10.15833 | 14.25417 |
| *Buthus elongatus* | -5.1 | 36.66 | 35 | 41 | 68 | 1075 | 23 | 24 | 69.46303 | 2 | 814 | 594.0188 | 39.0866 | 10.98333 | 13.05833 |
| *Buthus elongatus* | -4.9 | 36.53 | 29 | 49 | 68 | 171 | 13 | 23 | 76.17278 | 1 | 637 | 460.8858 | 41.55199 | 9.05833 | 17.17083 |
| *Buthus elongatus* | -5.16 | 36.44 | 31 | 44 | 70 | 72 | 12 | 25 | 78.23482 | 1 | 647 | 448.9424 | 41.3928 | 8.81667 | 17.65833 |
| *Buthus elongatus* | -4.9 | 36.53333 | 29 | 49 | 65 | 171 | 15 | 22 | 76.17278 | 1 | 637 | 460.8858 | 41.55199 | 9.05833 | 17.17083 |
| *Buthus elongatus* | -4.86479 | 36.74415 | 35 | 39 | 71 | 601 | 26 | 26 | 69.4925 | 2 | 684 | 555.9763 | 41.44737 | 11.025 | 15.4875 |
| *Buthus elongatus* | -4.77041 | 36.62631 | 33 | 49 | 66 | 426 | 23 | 18 | 72.6991 | 1 | 643 | 531.9084 | 41.33598 | 10.41667 | 16.14167 |
| *Buthus elongatus* | -4.75975 | 36.62874 | 35 | 46 | 66 | 424 | 22 | 19 | 72.70527 | 1 | 642 | 531.4937 | 41.30435 | 10.45 | 16.15833 |
| *Buthus elongatus* | -4.75622 | 36.62854 | 30 | 50 | 63 | 369 | 25 | 20 | 73.10315 | 1 | 632 | 525.3331 | 41.7336 | 10.39167 | 16.34583 |
| *Buthus elongatus* | -4.7519 | 36.62962 | 31 | 50 | 64 | 369 | 22 | 19 | 73.10315 | 1 | 632 | 525.3331 | 41.7336 | 10.39167 | 16.34583 |
| *Buthus elongatus* | -4.75875 | 36.62204 | 33 | 47 | 64 | 507 | 26 | 20 | 72.05234 | 1 | 651 | 535.257 | 41.0105 | 10.41667 | 15.85 |
| *Buthus elongatus* | -4.60811 | 36.64141 | 32 | 47 | 66 | 375 | 25 | 21 | 71.99717 | 1 | 606 | 524.9984 | 40.92443 | 10.10833 | 16.3125 |
| *Buthus elongatus* | -4.53693 | 36.62488 | 31 | 42 | 71 | 318 | 15 | 26 | 71.93468 | 1 | 597 | 512.6644 | 40.61181 | 9.625 | 16.57083 |
| *Buthus elongatus* | -4.53652 | 36.62287 | 30 | 48 | 68 | 318 | 18 | 22 | 71.93468 | 1 | 597 | 512.6644 | 40.61181 | 9.625 | 16.57083 |
| *Buthus elongatus* | -4.66137 | 36.63384 | 33 | 44 | 64 | 451 | 23 | 23 | 71.60326 | 1 | 624 | 536.6794 | 41.0079 | 10.375 | 16.02917 |
| *Buthus elongatus* | -4.61826 | 36.60813 | 31 | 49 | 67 | 458 | 22 | 19 | 70.83607 | 1 | 633 | 523.6025 | 40.2664 | 9.825 | 15.9375 |
| *Buthus elongatus* | -4.86603 | 36.5317 | 32 | 44 | 69 | 169 | 15 | 24 | 76.22388 | 1 | 633 | 460.8102 | 41.7049 | 9.09167 | 17.1625 |
| *Buthus elongatus* | -4.89055 | 36.57029 | 34 | 48 | 63 | 890 | 18 | 18 | 69.8619 | 2 | 748 | 550.9599 | 39.42931 | 10.13333 | 14.275 |
| *Buthus elongatus* | -4.8903 | 36.57214 | 35 | 45 | 61 | 890 | 19 | 20 | 69.8619 | 2 | 748 | 550.9599 | 39.42931 | 10.13333 | 14.275 |
| *Buthus elongatus* | -4.88233 | 36.57377 | 34 | 46 | 62 | 826 | 18 | 19 | 70.43671 | 2 | 733 | 545.8832 | 39.42057 | 10.09167 | 14.49583 |
| *Buthus elongatus* | -4.89675 | 36.58474 | 37 | 46 | 66 | 1073 | 18 | 17 | 69.21259 | 2 | 782 | 570.1489 | 38.67924 | 10.25 | 13.46667 |
| *Buthus elongatus* | -4.89469 | 36.58419 | 38 | 44 | 66 | 1073 | 24 | 18 | 69.21259 | 2 | 782 | 570.1489 | 38.67924 | 10.25 | 13.46667 |
| *Buthus elongatus* | -5.09403 | 36.66767 | 35 | 41 | 68 | 1107 | 22 | 24 | 69.47366 | 2 | 822 | 598.8467 | 38.96714 | 11.06667 | 12.94167 |
| *Buthus elongatus* | -5.08311 | 36.66406 | 35 | 41 | 66 | 1069 | 17 | 24 | 69.34099 | 2 | 809 | 592.7759 | 39.10714 | 10.95 | 13.01667 |
| *Buthus elongatus* | -5.05134 | 36.67938 | 37 | 38 | 68 | 1277 | 24 | 25 | 68.11819 | 3 | 853 | 610.106 | 38.80208 | 11.175 | 12.14583 |
| *Buthus elongatus* | -4.96411 | 36.72222 | 36 | 45 | 64 | 1120 | 26 | 20 | 67.80581 | 3 | 800 | 596.3576 | 37.87879 | 10.41667 | 12.66667 |
| *Buthus elongatus* | -4.93509 | 36.72334 | 39 | 35 | 73 | 669 | 20 | 26 | 69.86441 | 2 | 706 | 552.4135 | 39.27649 | 10.13333 | 14.51667 |

| *Buthus garcialorcai* | -3.53409 | 37.2359 | 37 | 41 | 69 | 1223 | 27 | 22 | 52.80608 | 5 | 520 | 671.2963 | 39.93711 | 12.7 | 12.725 |
| --- | --- | --- | --- | --- | --- | --- | --- | --- | --- | --- | --- | --- | --- | --- | --- |
| *Buthus garcialorcai* | -3.4 | 37.1 | 38 | 44 | 64 | 2276 | 26 | 18 | 50.50738 | 12 | 847 | 759.6899 | 35.19608 | 11.96667 | 6.03333 |
| *Buthus garcialorcai* | -3.47 | 37.37 | 35 | 44 | 67 | 1007 | 29 | 21 | 51.38554 | 4 | 455 | 684.1484 | 40.50505 | 13.36667 | 13.8 |
| *Buthus garcialorcai* | -3.32 | 37.02 | 36 | 43 | 65 | 2444 | 23 | 21 | 48.97701 | 14 | 904 | 745.5325 | 37.43855 | 12.69167 | 5.9625 |
| *Buthus garcialorcai* | -3.99 | 36.99 | 37 | 40 | 75 | 917 | 18 | 24 | 58.25664 | 3 | 517 | 637.1504 | 42.03587 | 13.28333 | 14.31667 |
| *Buthus garcialorcai* | -3.64 | 37.05 | 38 | 31 | 77 | 960 | 20 | 31 | 54.5748 | 4 | 468 | 654.6332 | 41.69279 | 13.3 | 14.44167 |
| *Buthus garcialorcai* | -3.51 | 37.69 | 37 | 39 | 73 | 1009 | 24 | 23 | 50.28177 | 5 | 473 | 694.8883 | 40.56225 | 13.46667 | 13.81667 |
| *Buthus garcialorcai* | -3.53874 | 37.24114 | 36 | 40 | 71 | 1223 | 24 | 24 | 52.80608 | 5 | 520 | 671.2963 | 39.93711 | 12.7 | 12.725 |
| *Buthus garcialorcai* | -3.46058 | 37.18053 | 38 | 43 | 66 | 1207 | 26 | 19 | 52.34317 | 5 | 503 | 674.1003 | 40.13975 | 12.925 | 12.94583 |
| *Buthus garcialorcai* | -3.54924 | 37.17235 | 35 | 45 | 71 | 949 | 24 | 20 | 52.84938 | 4 | 448 | 660.416 | 41.22257 | 13.15 | 14.45833 |
| *Buthus garcialorcai* | -3.55064 | 37.17369 | 35 | 46 | 72 | 942 | 26 | 20 | 52.97799 | 4 | 448 | 661.5035 | 41.11979 | 13.15833 | 14.5375 |
| *Buthus garcialorcai* | -3.5108 | 37.12822 | 34 | 40 | 71 | 1069 | 22 | 26 | 52.99942 | 4 | 479 | 662.9564 | 39.86773 | 12.55833 | 13.24583 |
| *Buthus garcialorcai* | -3.53874 | 37.24114 | 36 | 40 | 71 | 1223 | 24 | 24 | 52.80608 | 5 | 520 | 671.2963 | 39.93711 | 12.7 | 12.725 |
| *Buthus garcialorcai* | -3.73447 | 36.98011 | 36 | 44 | 69 | 1271 | 23 | 20 | 55.31018 | 5 | 567 | 665.131 | 40.22989 | 12.83333 | 12.65833 |
| *Buthus garcialorcai* | -3.52908 | 37.08321 | 37 | 42 | 70 | 1284 | 22 | 22 | 53.06424 | 5 | 542 | 671.2182 | 38.9862 | 12.24167 | 12.09583 |
| *Buthus halius* | -7.39579 | 41.28179 | 36 | 47 | 63 | 246 | 31 | 18 | 54.37619 | 17 | 1258 | 423.9095 | 43.31723 | 8.96667 | 14.04167 |
| *Buthus halius* | -8.13454 | 40.8892 | 30 | 56 | 54 | 834 | 21 | 14 | 55.63508 | 21 | 1515 | 513.3779 | 36.87316 | 8.33333 | 11.925 |
| *Buthus halius* | -7.54931 | 40.37264 | 40 | 42 | 63 | 1189 | 22 | 18 | 57.4181 | 16 | 1453 | 568.5781 | 32.37886 | 7.35 | 10.24167 |
| *Buthus halius* | -7.54638 | 40.38579 | 39 | 41 | 63 | 865 | 30 | 19 | 57.03187 | 16 | 1341 | 553.298 | 35.11396 | 8.21667 | 11.825 |
| *Buthus halius* | -8.36439 | 37.36972 | 32 | 48 | 63 | 156 | 17 | 19 | 69.25877 | 2 | 558 | 442.3652 | 43.59259 | 9.80833 | 16.4375 |
| *Buthus halius* | -8.11752 | 37.24364 | 35 | 41 | 66 | 296 | 17 | 24 | 70.60775 | 2 | 588 | 480.9718 | 41.84174 | 9.95833 | 16.15417 |
| *Buthus halius* | -4.12779 | 39.13706 | 36 | 46 | 68 | 712 | 23 | 17 | 43.83385 | 7 | 416 | 717.7916 | 37.03416 | 11.925 | 14.44583 |
| *Buthus halius* | -3.49132 | 39.32845 | 36 | 46 | 73 | 715 | 20 | 18 | 42.55761 | 8 | 407 | 735.1433 | 37.67677 | 12.43333 | 14.60833 |
| *Buthus halius* | -4.64028 | 41.55783 | 30 | 56 | 73 | 700 | 13 | 14 | 33.11058 | 16 | 405 | 640.9468 | 40.23179 | 12.15 | 12.30833 |
| *Buthus halius* | -4.80915 | 41.54194 | 27 | 60 | 70 | 700 | 15 | 13 | 33.31438 | 17 | 423 | 633.6694 | 39.92705 | 11.85833 | 12.2375 |
| *Buthus halius* | -3.99994 | 40.7631 | 31 | 56 | 62 | 1555 | 22 | 13 | 41.11571 | 22 | 840 | 643.0648 | 31.14379 | 7.94167 | 8.0625 |
| *Buthus halius* | -8.46 | 38.21 | 21 | 66 | 61 | 90 | 11 | 13 | 64.13635 | 4 | 614 | 455.4624 | 45.21276 | 10.625 | 16.4625 |
| *Buthus halius* | -8.08 | 38.51 | 32 | 50 | 62 | 231 | 15 | 18 | 61.00609 | 4 | 638 | 503.2672 | 44.3904 | 11.40833 | 16.1375 |
| *Buthus halius* | -8.07 | 38.44 | 27 | 55 | 63 | 181 | 15 | 18 | 61.09791 | 4 | 613 | 508.0107 | 45.12579 | 11.95833 | 16.24583 |
| *Buthus halius* | -8.03 | 38.52 | 32 | 51 | 63 | 212 | 17 | 16 | 61.07029 | 4 | 622 | 500.3565 | 44.58496 | 11.45833 | 16.12083 |
| *Buthus halius* | -8.02 | 38.53 | 34 | 50 | 65 | 224 | 13 | 17 | 61.12473 | 4 | 625 | 500.9815 | 44.45525 | 11.425 | 16.0375 |
| *Buthus halius* | -8 | 38.53 | 29 | 57 | 64 | 244 | 13 | 14 | 60.99718 | 4 | 631 | 510.7857 | 44.49746 | 11.65833 | 15.99583 |
| *Buthus halius* | -7.91 | 37.19 | 32 | 49 | 60 | 323 | 22 | 19 | 70.86333 | 2 | 594 | 494.1031 | 41.5978 | 10.06667 | 16.03333 |
| *Buthus halius* | -7.81 | 41.36 | 29 | 61 | 53 | 1079 | 17 | 9 | 51.38025 | 20 | 1317 | 524.5554 | 35.35127 | 7.88333 | 10.29167 |
| *Buthus halius* | -7.8 | 41.2 | 36 | 44 | 64 | 203 | 23 | 20 | 55.93103 | 16 | 1255 | 421.3082 | 44.13431 | 9.09167 | 14.2625 |
| *Buthus halius* | -7.47 | 41.67 | 28 | 60 | 56 | 698 | 21 | 12 | 52.46816 | 22 | 1352 | 511.3327 | 39.02003 | 9.09167 | 11.89583 |
| *Buthus halius* | -7.43 | 40.2 | 28 | 61 | 59 | 479 | 25 | 12 | 58.52318 | 10 | 1029 | 545.9645 | 39.48413 | 9.95 | 14.45 |
| *Buthus halius* | -7.02 | 40.14 | 36 | 49 | 60 | 404 | 22 | 15 | 55.18619 | 9 | 784 | 567.1273 | 39.53489 | 10.2 | 15.06667 |
| *Buthus halius* | -6.79 | 40.16 | 29 | 56 | 61 | 513 | 21 | 15 | 54.72471 | 10 | 762 | 568.7431 | 37.58234 | 9.50833 | 14.3875 |
| *Buthus halius* | -6.56 | 37.89 | 35 | 47 | 68 | 656 | 22 | 18 | 62.54305 | 4 | 677 | 601.447 | 39.26901 | 11.19167 | 14.69583 |
| *Buthus halius* | -6.44 | 38.41 | 36 | 41 | 67 | 525 | 20 | 23 | 60.0291 | 6 | 635 | 644.8267 | 40.9188 | 12.76667 | 15.51667 |

**Table S7.** Continued.

**Table S7.** Continued.

| *Buthus halius* | -6.41 | 39.7 | 34 | 50 | 62 | 334 | 18 | 16 | 53.97627 | 7 | 549 | 645.0382 | 37.48558 | 10.83333 | 16.23333 |
| --- | --- | --- | --- | --- | --- | --- | --- | --- | --- | --- | --- | --- | --- | --- | --- |
| *Buthus halius* | -6.36 | 40.5 | 40 | 43 | 64 | 877 | 17 | 17 | 55.12473 | 15 | 1016 | 577.6946 | 37.6615 | 9.71667 | 11.90833 |
| *Buthus halius* | -6.33 | 38.98 | 26 | 60 | 62 | 289 | 18 | 14 | 59.06242 | 6 | 546 | 652.1166 | 40.46858 | 12.66667 | 16.51667 |
| *Buthus halius* | -6.32 | 40.9 | 31 | 57 | 61 | 733 | 15 | 12 | 55.93499 | 17 | 1091 | 591.275 | 39.53069 | 10.95 | 12.43333 |
| *Buthus halius* | -6.23 | 38.1 | 38 | 44 | 62 | 761 | 23 | 18 | 61.50015 | 5 | 712 | 643.2923 | 39.05229 | 11.95 | 14.325 |
| *Buthus halius* | -6.23 | 38.97 | 32 | 47 | 67 | 277 | 19 | 21 | 59.06109 | 6 | 545 | 646.1688 | 40.20563 | 12.38333 | 16.525 |
| *Buthus halius* | -5.84 | 39.46 | 24 | 60 | 61 | 517 | 17 | 16 | 52.37703 | 6 | 565 | 660.8666 | 39.22829 | 12.2 | 15.14167 |
| *Buthus halius* | -5.67 | 38.25 | 35 | 42 | 70 | 525 | 17 | 23 | 60.31855 | 5 | 632 | 663.4076 | 41.58998 | 13.55833 | 15.5375 |
| *Buthus halius* | -5.63 | 37.49 | 32 | 41 | 74 | 164 | 16 | 26 | 66.5498 | 2 | 574 | 605.9008 | 42.55853 | 12.725 | 17.52917 |
| *Buthus halius* | -5.57 | 39.88 | 34 | 49 | 66 | 315 | 16 | 17 | 46.86467 | 6 | 435 | 634.717 | 39.43701 | 11.79167 | 15.69583 |
| *Buthus halius* | -5.5 | 38.18 | 35 | 48 | 63 | 499 | 24 | 17 | 61.68034 | 4 | 612 | 658.0773 | 40.72623 | 12.99167 | 15.67083 |
| *Buthus halius* | -5.42 | 39.4 | 38 | 43 | 64 | 717 | 21 | 19 | 50.83635 | 6 | 580 | 641.9962 | 38.19209 | 11.26667 | 14.05 |
| *Buthus halius* | -5.42 | 40.33 | 29 | 58 | 64 | 1136 | 16 | 13 | 42.38844 | 15 | 649 | 617.5101 | 36.41636 | 9.94167 | 10.4875 |
| *Buthus halius* | -5.22 | 39.19 | 36 | 46 | 65 | 457 | 19 | 18 | 52.52902 | 5 | 491 | 628.792 | 39.03061 | 11.475 | 15.12917 |
| *Buthus halius* | -5.01 | 40.3 | 32 | 52 | 67 | 929 | 26 | 16 | 36.39203 | 13 | 462 | 620.76 | 36.19311 | 9.80833 | 11.75417 |
| *Buthus halius* | -5.01 | 38.18 | 36 | 46 | 66 | 544 | 24 | 18 | 58.92695 | 5 | 589 | 658.709 | 40.48742 | 12.875 | 15.32083 |
| *Buthus halius* | -4.61 | 40.3 | 29 | 56 | 65 | 688 | 22 | 15 | 34.23711 | 11 | 353 | 633.7531 | 36.56584 | 10.275 | 13.37083 |
| *Buthus halius* | -4.51 | 39.61 | 36 | 48 | 66 | 859 | 21 | 17 | 40.67141 | 10 | 462 | 678.9274 | 35.57047 | 10.6 | 13.26667 |
| *Buthus halius* | -4.4 | 40.33 | 26 | 58 | 66 | 814 | 21 | 16 | 34.44141 | 12 | 363 | 659.7314 | 35.59814 | 10.21667 | 13.1 |
| *Buthus halius* | -4.21 | 39.75 | 35 | 45 | 68 | 862 | 23 | 20 | 39.25439 | 10 | 433 | 710.1807 | 36.92348 | 11.74167 | 13.84583 |
| *Buthus halius* | -4.15 | 40.54 | 27 | 56 | 69 | 969 | 19 | 17 | 34.61158 | 15 | 413 | 676.0485 | 36.04452 | 10.525 | 12.1875 |
| *Buthus halius* | -4.14 | 40.9 | 36 | 41 | 74 | 1026 | 18 | 23 | 30.74261 | 20 | 463 | 672.7403 | 35.73944 | 10.15 | 11.075 |
| *Buthus halius* | -4.03 | 38.84 | 32 | 48 | 73 | 685 | 17 | 21 | 46.1543 | 6 | 421 | 723.2911 | 38.30321 | 12.71667 | 14.65 |
| *Buthus halius* | -3.76 | 38.36 | 35 | 47 | 66 | 659 | 27 | 18 | 49.87177 | 5 | 435 | 696.3654 | 39.0528 | 12.575 | 15.07083 |
| *Buthus halius* | -3.73 | 38.1 | 36 | 35 | 76 | 302 | 10 | 28 | 53.08477 | 4 | 399 | 691.4887 | 41.21074 | 13.55833 | 17.47917 |
| *Buthus halius* | -3.49 | 38.39 | 37 | 43 | 67 | 911 | 26 | 19 | 47.41573 | 6 | 463 | 724.47 | 38.45382 | 12.76667 | 13.875 |
| *Buthus halius* | -3.34 | 38.82 | 31 | 45 | 74 | 739 | 13 | 24 | 44.50734 | 7 | 419 | 749.2891 | 39.76191 | 13.91667 | 14.775 |
| *Buthus halius* | -2.78 | 38.52 | 34 | 46 | 71 | 700 | 18 | 21 | 46.24841 | 6 | 401 | 752.2874 | 40.81921 | 14.45 | 14.8 |
| *Buthus halius* | -2.72 | 38.28 | 37 | 39 | 75 | 752 | 27 | 24 | 46.1581 | 6 | 406 | 727.7877 | 39.58333 | 13.3 | 14.325 |
| *Buthus halius* | -2.71 | 41.17 | 36 | 37 | 76 | 944 | 14 | 26 | 31.5183 | 16 | 435 | 666.3344 | 40.79365 | 12.85 | 11.63333 |
| *Buthus halius* | -8.91946 | 39.42217 | 33 | 43 | 66 | 336 | 20 | 24 | 58.95102 | 8 | 812 | 411.551 | 39.58333 | 7.6 | 14.81667 |
| *Buthus halius* | -8.60313 | 39.6521 | 36 | 43 | 70 | 177 | 13 | 20 | 56.83577 | 8 | 805 | 444.5887 | 42.91101 | 9.48333 | 15.5 |
| *Buthus halius* | -8.25194 | 39.55206 | 34 | 49 | 57 | 223 | 22 | 16 | 57.64583 | 7 | 803 | 475.4589 | 41.66666 | 9.54167 | 15.67917 |
| *Buthus halius* | -7.18933 | 40.80297 | 37 | 49 | 64 | 480 | 21 | 14 | 56.05324 | 15 | 1072 | 505.1387 | 41.20956 | 9.76667 | 13.61667 |
| *Buthus halius* | -8.21425 | 40.89012 | 37 | 43 | 56 | 799 | 28 | 20 | 55.32523 | 21 | 1500 | 500.231 | 37.17949 | 8.21667 | 11.90833 |
| *Buthus halius* | -3.74112 | 41.68605 | 35 | 45 | 77 | 799 | 14 | 20 | 29.39675 | 12 | 369 | 652.7289 | 39.58334 | 11.875 | 11.9125 |
| *Buthus halius* | -6.84397 | 42.46284 | 39 | 41 | 61 | 760 | 23 | 20 | 44.5209 | 30 | 1100 | 576.504 | 38.56961 | 10.06667 | 11.575 |
| *Buthus halius* | -7.86617 | 42.28559 | 28 | 57 | 56 | 273 | 18 | 15 | 49.34858 | 32 | 1635 | 510.0839 | 43.62934 | 11.3 | 13.69167 |
| *Buthus halius* | -5.96723 | 42.75669 | 35 | 45 | 63 | 1091 | 18 | 20 | 31.98557 | 41 | 982 | 592.297 | 38.50682 | 10.35833 | 9.24583 |
| *Buthus halius* | -6.48211 | 42.20176 | 35 | 48 | 61 | 1470 | 23 | 16 | 39.93204 | 35 | 1130 | 608.1118 | 34.73819 | 9.06667 | 7.7 |
| *Buthus halius* | -6.33354 | 41.87811 | 34 | 51 | 55 | 891 | 22 | 15 | 45.35843 | 21 | 825 | 597.7147 | 37.46959 | 10.26667 | 10.90833 |

**Table S7.** Continued.

| *Buthus iaspis* | -2.3034 | 37.0852 | 35 | 38 | 80 | 497 | 12 | 27 | 48.62464 | 3 | 298 | 646.2038 | 42.57188 | 13.325 | 16.14583 |
| --- | --- | --- | --- | --- | --- | --- | --- | --- | --- | --- | --- | --- | --- | --- | --- |
| *Buthus iaspis* | -2.26311 | 36.82814 | 30 | 49 | 78 | 35 | 12 | 21 | 52.26197 | 1 | 213 | 549.481 | 42.61006 | 11.29167 | 17.94583 |
| *Buthus iaspis* | -2.14305 | 36.73411 | 25 | 47 | 75 | 52 | 22 | 28 | 53.09848 | 1 | 229 | 532.4022 | 41.76509 | 10.60833 | 17.77083 |
| *Buthus iaspis* | -1.91 | 36.98 | 30 | 43 | 81 | 48 | 17 | 27 | 51.45484 | 2 | 231 | 544.8582 | 42.20945 | 11.01667 | 17.825 |
| *Buthus iaspis* | -2.07 | 37 | 32 | 44 | 80 | 231 | 14 | 24 | 49.63649 | 2 | 254 | 587.1439 | 42.8655 | 12.21667 | 17.11667 |
| *Buthus iaspis* | -2.1 | 37.1 | 31 | 44 | 79 | 333 | 15 | 24 | 46.57534 | 3 | 275 | 607.2383 | 42.66552 | 12.45833 | 16.54583 |
| *Buthus iaspis* | -2.41 | 37.04 | 32 | 47 | 77 | 389 | 22 | 21 | 48.57231 | 3 | 278 | 618.1955 | 41.92177 | 12.325 | 16.52083 |
| *Buthus iaspis* | -2.59 | 37.13 | 36 | 42 | 79 | 820 | 19 | 22 | 48.5605 | 4 | 364 | 673.6749 | 41.64087 | 13.45 | 14.83333 |
| *Buthus iaspis* | -2.63 | 37.17 | 32 | 47 | 74 | 1106 | 22 | 21 | 47.58969 | 5 | 439 | 684.5635 | 41.7422 | 13.81667 | 13.05 |
| *Buthus iaspis* | -2.72 | 37.43 | 36 | 40 | 79 | 931 | 17 | 24 | 47.73555 | 5 | 400 | 703.3506 | 40.57214 | 13.59167 | 13.87917 |
| *Buthus iaspis* | -2.7 | 37.54 | 36 | 35 | 78 | 742 | 11 | 29 | 47.05778 | 5 | 366 | 707.1693 | 41.47116 | 14.14167 | 14.82917 |
| *Buthus iaspis* | -1.34 | 37.59 | 31 | 46 | 79 | 92 | 16 | 23 | 46.11878 | 3 | 269 | 571.0893 | 43.11071 | 11.94167 | 17.84583 |
| *Buthus iaspis* | -1.81 | 37.53 | 34 | 37 | 79 | 450 | 10 | 29 | 42.03793 | 5 | 310 | 645.6725 | 42.80785 | 13.44167 | 16.14583 |
| *Buthus iaspis* | -2.03299 | 36.91618 | 31 | 43 | 80 | 262 | 16 | 26 | 51.31993 | 2 | 255 | 577.7915 | 42.44885 | 11.75833 | 16.92917 |
| *Buthus iaspis* | -2.13966 | 37.1046 | 31 | 49 | 79 | 426 | 14 | 20 | 46.4052 | 3 | 291 | 623.0424 | 42.5864 | 12.73333 | 16.25 |
| *Buthus iaspis* | -2.53812 | 37.15995 | 35 | 44 | 76 | 1011 | 20 | 21 | 47.11818 | 5 | 416 | 674.5553 | 41.07143 | 13.225 | 13.42917 |
| *Buthus iaspis* | -2.35787 | 37.09434 | 33 | 42 | 80 | 507 | 16 | 25 | 48.53663 | 3 | 297 | 648.7448 | 42.72152 | 13.5 | 16.26667 |
| *Buthus iaspis* | -2.30348 | 37.08472 | 35 | 38 | 80 | 497 | 12 | 27 | 48.62464 | 3 | 298 | 646.2038 | 42.57188 | 13.325 | 16.14583 |
| *Buthus iaspis* | -2.34767 | 37.07273 | 37 | 37 | 79 | 462 | 13 | 25 | 48.95837 | 3 | 287 | 641.0897 | 42.31392 | 13.075 | 16.2875 |
| *Buthus iaspis* | -2.39477 | 37.05315 | 38 | 36 | 81 | 408 | 15 | 26 | 48.57231 | 3 | 278 | 625.4072 | 42 | 12.6 | 16.475 |
| *Buthus iaspis* | -2.41918 | 37.01761 | 33 | 43 | 80 | 347 | 19 | 24 | 48.41181 | 3 | 278 | 607.3008 | 41.75287 | 12.10833 | 16.6625 |
| *Buthus iaspis* | -2.24604 | 36.8422 | 28 | 51 | 80 | 60 | 16 | 21 | 52.7791 | 1 | 218 | 557.8259 | 42.62701 | 11.46667 | 17.88333 |
| *Buthus iaspis* | -2.24606 | 36.81621 | 32 | 47 | 81 | 8 | 6 | 21 | 53.14512 | 1 | 213 | 545.3182 | 42.48737 | 11.21667 | 18.04167 |
| *Buthus iaspis* | -2.29654 | 36.83232 | 30 | 48 | 79 | 17 | 11 | 22 | 51.38217 | 1 | 211 | 543.4045 | 41.98595 | 10.95833 | 17.90417 |
| *Buthus iaspis* | -2.2007 | 36.75913 | 31 | 41 | 73 | 26 | 18 | 28 | 53.9721 | 1 | 217 | 532.5788 | 40.93916 | 10.31667 | 17.80833 |
| *Buthus iaspis* | -2.10458 | 36.77939 | 31 | 40 | 78 | 68 | 18 | 29 | 54.25451 | 1 | 226 | 544.5828 | 42.40102 | 11.06667 | 17.825 |
| *Buthus iaspis* | -2.19406 | 36.77191 | 31 | 47 | 76 | 48 | 17 | 22 | 53.2228 | 1 | 220 | 541.2155 | 41.34241 | 10.625 | 17.7875 |
| *Buthus iaspis* | -1.86874 | 37.06544 | 30 | 46 | 73 | 131 | 17 | 25 | 49.59322 | 2 | 250 | 554.4606 | 42.32705 | 11.21667 | 17.45833 |
| *Buthus ibericus* | -5.66458 | 36.62787 | 36 | 41 | 70 | 127 | 16 | 23 | 73.32302 | 0 | 632 | 482.1346 | 39.62963 | 8.91667 | 17.20833 |
| *Buthus ibericus* | -5.42347 | 36.70732 | 37 | 38 | 72 | 825 | 18 | 24 | 70.15685 | 2 | 809 | 563.3724 | 37.96296 | 9.90833 | 13.79583 |
| *Buthus ibericus* | -5.65 | 36.39 | 31 | 43 | 71 | 45 | 15 | 26 | 74.3387 | 0 | 710 | 449.8053 | 38.65854 | 7.925 | 17.59583 |
| *Buthus ibericus* | -5.66 | 36.52 | 33 | 47 | 63 | 335 | 17 | 20 | 72.77503 | 1 | 740 | 494.8845 | 39.53824 | 9.13333 | 16.43333 |
| *Buthus ibericus* | -5.42 | 36.68 | 38 | 35 | 69 | 1020 | 25 | 28 | 69.93053 | 2 | 853 | 581.3614 | 37.70287 | 10.06667 | 13.19167 |
| *Buthus ibericus* | -5.67 | 36.62 | 34 | 43 | 65 | 311 | 19 | 23 | 72.04559 | 1 | 701 | 497.7381 | 39.10534 | 9.03333 | 16.525 |
| *Buthus ibericus* | -5.805 | 36.759 | 37 | 33 | 79 | 111 | 13 | 30 | 70.65289 | 1 | 616 | 508.3217 | 41.97031 | 10.36667 | 17.55 |
| *Buthus ibericus* | -5.42 | 36.92 | 34 | 41 | 72 | 515 | 17 | 25 | 69.75719 | 1 | 703 | 550.402 | 40.87452 | 10.75 | 15.51667 |
| *Buthus ibericus* | -5.28 | 36.77 | 36 | 46 | 63 | 809 | 19 | 18 | 70.65267 | 2 | 763 | 572.47 | 39.92674 | 10.9 | 14.20833 |
| *Buthus ibericus* | -5.59 | 36.02 | 29 | 46 | 70 | 68 | 12 | 24 | 79.73013 | 0 | 839 | 442.9413 | 38.48039 | 7.85 | 17.71667 |
| *Buthus ibericus* | -5.85 | 36.6 | 31 | 50 | 70 | 116 | 12 | 19 | 70.93328 | 1 | 634 | 501.8277 | 41.39119 | 10.01667 | 17.50833 |
| *Buthus ibericus* | -5.81 | 36.76 | 36 | 33 | 78 | 153 | 13 | 30 | 70.19558 | 1 | 632 | 512.9165 | 41.93333 | 10.48333 | 17.41667 |
| *Buthus ibericus* | -5.39469 | 36.74628 | 39 | 37 | 69 | 1236 | 20 | 24 | 67.99429 | 3 | 895 | 593.4121 | 37.14637 | 10.06667 | 12.18333 |

**Table S7.** Continued.

| *Buthus ibericus* | -5.39564 | 36.76826 | 37 | 36 | 68 | 1106 | 22 | 26 | 68.8988 | 2 | 851 | 584.5374 | 37.46891 | 10.04167 | 12.82083 |
| --- | --- | --- | --- | --- | --- | --- | --- | --- | --- | --- | --- | --- | --- | --- | --- |
| *Buthus ibericus* | -5.49647 | 36.75441 | 35 | 45 | 69 | 508 | 25 | 20 | 70.85883 | 1 | 735 | 536.5653 | 40.69011 | 10.41667 | 15.625 |
| *Buthus ibericus* | -5.49016 | 36.7572 | 34 | 43 | 68 | 758 | 25 | 23 | 70.3751 | 2 | 791 | 557.2557 | 39.74116 | 10.49167 | 14.62083 |
| *Buthus ibericus* | -5.46252 | 36.76374 | 36 | 40 | 68 | 562 | 21 | 23 | 70.37696 | 1 | 749 | 527.769 | 39.49865 | 9.71667 | 14.94167 |
| *Buthus ibericus* | -5.45636 | 36.76294 | 35 | 44 | 65 | 737 | 22 | 21 | 70.32679 | 1 | 786 | 543.693 | 38.72355 | 9.75833 | 14.22083 |
| *Buthus ibericus* | -5.38901 | 36.80125 | 36 | 41 | 67 | 728 | 21 | 23 | 70.76572 | 1 | 752 | 561.4731 | 38.66539 | 10.09167 | 14.59583 |
| *Buthus ibericus* | -5.37699 | 36.7871 | 38 | 36 | 69 | 1192 | 20 | 25 | 68.62485 | 3 | 865 | 596.9652 | 37.46959 | 10.26667 | 12.75833 |
| *Buthus ibericus* | -5.44158 | 36.76634 | 39 | 39 | 66 | 1143 | 16 | 22 | 68.03876 | 3 | 880 | 578.884 | 37.53156 | 9.90833 | 12.62917 |
| *Buthus ibericus* | -5.30947 | 36.68434 | 36 | 38 | 68 | 1156 | 22 | 27 | 69.86622 | 2 | 858 | 599.4409 | 37.39471 | 10.35833 | 12.62083 |
| *Buthus ibericus* | -5.59195 | 36.5262 | 35 | 41 | 61 | 673 | 18 | 24 | 73.16916 | 1 | 816 | 509.0715 | 38.14655 | 8.85 | 14.60833 |
| *Buthus ibericus* | -5.57474 | 36.59988 | 35 | 43 | 68 | 331 | 15 | 21 | 72.9826 | 1 | 715 | 496.3799 | 39.61925 | 9.19167 | 16.32917 |
| *Buthus ibericus* | -5.35879 | 36.74954 | 37 | 40 | 71 | 1144 | 25 | 23 | 69.06087 | 2 | 855 | 592.6908 | 37.68248 | 10.325 | 12.7125 |
| *Buthus ibericus* | -5.33682 | 36.7453 | 37 | 44 | 65 | 809 | 14 | 19 | 70.60335 | 2 | 776 | 567.4437 | 38.72181 | 10.3 | 14.01667 |
| *Buthus ibericus* | -5.33441 | 36.74358 | 36 | 46 | 65 | 809 | 13 | 18 | 70.60335 | 2 | 776 | 567.4437 | 38.72181 | 10.3 | 14.01667 |
| *Buthus manchego* | -0.86022 | 38.64095 | 35 | 41 | 78 | 539 | 17 | 24 | 36.6811 | 10 | 362 | 665.6113 | 41.40379 | 13.125 | 14.97083 |
| *Buthus manchego* | -0.52825 | 38.42624 | 32 | 45 | 80 | 177 | 14 | 23 | 44.98509 | 6 | 338 | 563.1505 | 43.88069 | 11.89167 | 17.17917 |
| *Buthus manchego* | -0.5252 | 38.28249 | 30 | 50 | 76 | 5 | 9 | 20 | 48.37321 | 5 | 300 | 520.5584 | 43.50736 | 10.83333 | 18.04167 |
| *Buthus manchego* | -1.58035 | 37.81443 | 35 | 44 | 77 | 651 | 17 | 21 | 40.35989 | 6 | 352 | 656.3032 | 41.10215 | 12.74167 | 14.72083 |
| *Buthus manchego* | -3.00345 | 39.0631 | 39 | 38 | 77 | 743 | 20 | 23 | 44.6167 | 7 | 424 | 747.3864 | 39.45087 | 13.65 | 14.55 |
| *Buthus manchego* | -2.72649 | 38.91908 | 34 | 42 | 75 | 923 | 16 | 24 | 43.20354 | 8 | 458 | 762.8837 | 40.48611 | 14.575 | 13.6125 |
| *Buthus manchego* | -2.1373 | 40.29821 | 37 | 44 | 65 | 1204 | 22 | 18 | 35.06757 | 18 | 556 | 669.5419 | 40.01068 | 12.48333 | 11.1 |
| *Buthus manchego* | -1.96651 | 40.02898 | 37 | 44 | 68 | 1241 | 21 | 19 | 31.5575 | 19 | 540 | 672.9897 | 40.03165 | 12.65 | 10.71667 |
| *Buthus manchego* | -2.29 | 40.23 | 36 | 40 | 77 | 957 | 16 | 24 | 37.40616 | 15 | 505 | 683.5318 | 38.9862 | 12.24167 | 12.67917 |
| *Buthus manchego* | -1.24 | 39.61 | 31 | 47 | 79 | 784 | 12 | 22 | 30.97046 | 18 | 398 | 667.699 | 41.03773 | 13.05 | 13.30833 |
| *Buthus manchego* | -1.82 | 39.54 | 32 | 48 | 75 | 880 | 17 | 19 | 32.22644 | 15 | 427 | 701.623 | 39.81199 | 13.05833 | 13.19583 |
| *Buthus manchego* | -1.05 | 39.09 | 34 | 42 | 76 | 566 | 17 | 23 | 34.64341 | 12 | 373 | 664.6068 | 39.63745 | 12.20833 | 14.57917 |
| *Buthus manchego* | -1.02 | 38.68 | 34 | 46 | 80 | 656 | 15 | 20 | 37.20261 | 10 | 368 | 681.1398 | 40.13499 | 12.88333 | 14.31667 |
| *Buthus manchego* | -1.36 | 38.49 | 33 | 46 | 80 | 592 | 11 | 21 | 37.07088 | 9 | 350 | 682.184 | 40.68323 | 13.1 | 14.975 |
| *Buthus manchego* | -1.64 | 38.5 | 35 | 43 | 81 | 480 | 15 | 22 | 36.43258 | 9 | 345 | 681.9885 | 41.10082 | 13.31667 | 15.60833 |
| *Buthus manchego* | -2.83 | 39.12 | 38 | 41 | 77 | 748 | 11 | 21 | 43.69277 | 7 | 425 | 752.2159 | 39.86742 | 14.03333 | 14.53333 |
| *Buthus manchego* | -2.66873 | 39.68769 | 36 | 40 | 77 | 875 | 16 | 24 | 39.9341 | 11 | 462 | 717.6661 | 38.73598 | 12.66667 | 13.45833 |
| *Buthus manchego* | -2.25433 | 40.18696 | 36 | 45 | 75 | 1032 | 16 | 19 | 36.35072 | 15 | 516 | 676.2299 | 39.5032 | 12.325 | 12.1625 |
| *Buthus manchego* | -2.42828 | 39.40165 | 28 | 53 | 78 | 723 | 13 | 19 | 40.76063 | 9 | 429 | 729.8488 | 39.66963 | 13.40833 | 14.44583 |
| *Buthus montanus* | -3.02317 | 37.10985 | 35 | 50 | 63 | 2127 | 24 | 15 | 47.74924 | 12 | 761 | 729.1043 | 36.59091 | 12.075 | 7.42083 |
| *Buthus montanus* | -3.25324 | 37.27948 | 35 | 41 | 73 | 1073 | 20 | 24 | 50.3316 | 5 | 453 | 690.0762 | 40.40786 | 13.375 | 13.5875 |
| *Buthus montanus* | -3.3 | 36.82 | 34 | 45 | 68 | 1199 | 24 | 20 | 53.55968 | 5 | 510 | 644.4993 | 40.44118 | 12.375 | 13.09583 |
| *Buthus montanus* | -3.06 | 37.18 | 35 | 41 | 76 | 1209 | 19 | 24 | 48.81568 | 6 | 483 | 714.0294 | 40.82125 | 14.08333 | 12.91667 |
| *Buthus montanus* | -3.21 | 37.13 | 36 | 43 | 64 | 2031 | 23 | 21 | 48.43407 | 11 | 742 | 754.2007 | 36.49759 | 12.59167 | 7.0875 |
| *Buthus montanus* | -3 | 37.11 | 36 | 45 | 63 | 2133 | 26 | 19 | 47.56698 | 12 | 759 | 731.3731 | 36.4394 | 12.025 | 7.37083 |
| *Buthus montanus* | -2.97 | 37.09 | 38 | 43 | 63 | 2164 | 22 | 19 | 47.73102 | 12 | 782 | 720.6861 | 37.18145 | 12.15833 | 7.59583 |
| *Buthus montanus* | -3.17 | 36.95 | 33 | 46 | 75 | 969 | 20 | 21 | 51.51052 | 4 | 442 | 653.1583 | 40.95011 | 12.85833 | 14.27083 |

**Table S7.** Continued.

| *Buthus montanus* | -3.01 | 36.96 | 35 | 41 | 75 | 534 | 19 | 24 | 51.93305 | 3 | 355 | 616.1985 | 41.58221 | 12.30833 | 16.1125 |
| --- | --- | --- | --- | --- | --- | --- | --- | --- | --- | --- | --- | --- | --- | --- | --- |
| *Buthus montanus* | -3.06 | 36.95 | 38 | 38 | 79 | 535 | 13 | 24 | 52.87891 | 3 | 355 | 616.0683 | 41.55405 | 12.3 | 16.19167 |
| *Buthus montanus* | -3.15 | 37.37 | 30 | 45 | 75 | 891 | 15 | 25 | 49.24664 | 5 | 408 | 697.5325 | 41.51961 | 14.11667 | 14.53333 |
| *Buthus montanus* | -3.17868 | 37.1761 | 35 | 47 | 67 | 1368 | 20 | 18 | 49.90833 | 6 | 529 | 704.5502 | 39.67066 | 13.25 | 11.95 |
| *Buthus montanus* | -3.20041 | 36.96475 | 36 | 43 | 72 | 1418 | 20 | 22 | 51.06964 | 6 | 558 | 673.4155 | 40.50207 | 13.04167 | 11.87083 |
| *Buthus montanus* | -2.91273 | 37.09186 | 39 | 44 | 66 | 2108 | 24 | 18 | 47.74615 | 11 | 760 | 723.1874 | 37.23404 | 12.25 | 7.8 |
| *Buthus montanus* | -2.88406 | 37.09862 | 37 | 46 | 64 | 2205 | 25 | 17 | 47.36528 | 12 | 784 | 730.908 | 36.80766 | 12.18333 | 7.40833 |
| *Buthus montanus* | -2.83213 | 37.07841 | 36 | 43 | 68 | 2293 | 24 | 21 | 47.15311 | 13 | 805 | 733.6346 | 37.33734 | 12.43333 | 7.16667 |
| *Buthus occitanus* | 4.145548 | 43.80966 | 38 | 40 | 70 | 117 | 15 | 22 | 37.04921 | 24 | 736 | 618.2985 | 35.61174 | 9.50833 | 14.14583 |
| *Buthus occitanus* | 3.83626 | 43.88868 | 42 | 34 | 75 | 238 | 21 | 24 | 33.1582 | 25 | 718 | 609.3941 | 35.16026 | 9.14167 | 13.27083 |
| *Buthus occitanus* | 3.74023 | 43.81738 | 40 | 36 | 73 | 217 | 16 | 23 | 32.74612 | 24 | 687 | 606.4182 | 35.30362 | 9.10833 | 13.42917 |
| *Buthus occitanus* | 2.59025 | 42.61301 | 39 | 42 | 60 | 587 | 20 | 19 | 20.61981 | 34 | 758 | 562.5826 | 35.26971 | 8.5 | 11.89167 |
| *Buthus occitanus* | 5.770039 | 43.32747 | 43 | 29 | 70 | 857 | 23 | 28 | 35.19674 | 21 | 877 | 575.9793 | 35.43388 | 8.575 | 11.07917 |
| *Buthus occitanus* | 1.874397 | 41.27732 | 36 | 40 | 75 | 348 | 16 | 24 | 28.30455 | 26 | 665 | 550.9372 | 37.74105 | 9.13333 | 14.45 |
| *Buthus occitanus* | -1.23425 | 42.58432 | 43 | 28 | 70 | 536 | 15 | 28 | 24.15371 | 33 | 681 | 581.864 | 37.43734 | 9.95833 | 12.72083 |
| *Buthus occitanus* | -1.37161 | 42.00039 | 42 | 34 | 78 | 424 | 17 | 24 | 23.89698 | 23 | 422 | 624.4774 | 38.90866 | 10.93333 | 13.80833 |
| *Buthus occitanus* | -1.70457 | 42.05188 | 43 | 36 | 78 | 313 | 13 | 22 | 24.98018 | 23 | 402 | 614.7559 | 39.61309 | 11.09167 | 14.14583 |
| *Buthus occitanus* | -0.41054 | 39.69869 | 34 | 44 | 69 | 498 | 24 | 22 | 32.25394 | 16 | 424 | 587.0269 | 40.95679 | 11.05833 | 14.5625 |
| *Buthus occitanus* | -0.73151 | 40.11182 | 34 | 47 | 74 | 938 | 17 | 19 | 32.43557 | 19 | 407 | 651.5692 | 42.75362 | 13.76667 | 11.83333 |
| *Buthus occitanus* | -0.73 | 40.11 | 34 | 48 | 73 | 938 | 18 | 18 | 32.43557 | 19 | 407 | 651.5692 | 42.75362 | 13.76667 | 11.83333 |
| *Buthus occitanus* | -0.02 | 39.92 | 33 | 47 | 77 | 4 | 8 | 20 | 44.57354 | 11 | 427 | 535.938 | 40.34722 | 9.68333 | 16.975 |
| *Buthus occitanus* | 1.79 | 41.36 | 39 | 39 | 76 | 284 | 16 | 23 | 27.26947 | 27 | 647 | 569.8091 | 39.00919 | 9.90833 | 14.6375 |
| *Buthus occitanus* | 1.32 | 41.14 | 33 | 47 | 70 | 42 | 15 | 20 | 35.27141 | 16 | 527 | 528.5164 | 38.55508 | 8.98333 | 15.625 |
| *Buthus occitanus* | 0.43 | 40.97 | 34 | 42 | 71 | 303 | 21 | 24 | 35.99714 | 13 | 507 | 595.9961 | 39.201 | 10.46667 | 15.125 |
| *Buthus occitanus* | 0.32 | 40.91 | 37 | 38 | 71 | 556 | 24 | 24 | 32.94413 | 17 | 526 | 615.3361 | 39.68676 | 11.19167 | 13.27917 |
| *Buthus occitanus* | 0.59 | 40.77 | 33 | 46 | 77 | 64 | 14 | 22 | 40.58075 | 14 | 547 | 563.4465 | 40.6168 | 10.31667 | 16.94167 |
| *Buthus occitanus* | -0.05 | 40.99 | 42 | 35 | 79 | 543 | 15 | 23 | 31.27569 | 19 | 443 | 654.4375 | 41.53226 | 12.875 | 13.67083 |
| *Buthus occitanus* | -0.62 | 40.81 | 38 | 41 | 71 | 1048 | 21 | 20 | 36.65 | 22 | 459 | 650.7542 | 43.18885 | 13.95 | 10.70833 |
| *Buthus occitanus* | -1.3 | 40.95 | 35 | 44 | 75 | 875 | 15 | 21 | 46.01616 | 16 | 409 | 661.35 | 43 | 13.975 | 12.05417 |
| *Buthus occitanus* | -1.34 | 39.88 | 33 | 47 | 78 | 986 | 14 | 20 | 30.45207 | 23 | 420 | 680.4872 | 40.95092 | 13.35 | 12.09167 |
| *Buthus occitanus* | -0.8 | 39.42 | 37 | 38 | 77 | 393 | 25 | 26 | 34.7229 | 14 | 387 | 598.2 | 41.30435 | 11.4 | 15.375 |
| *Buthus occitanus* | -2.09 | 42.25 | 38 | 40 | 76 | 577 | 22 | 22 | 26.33867 | 24 | 440 | 600.9267 | 39.95187 | 11.06667 | 12.49167 |
| *Buthus occitanus* | -1.53 | 42.18 | 41 | 34 | 77 | 315 | 13 | 25 | 24.16863 | 24 | 458 | 611.6365 | 38.8285 | 10.71667 | 14.10833 |
| *Buthus occitanus* | 3.56 | 43.49 | 39 | 38 | 71 | 85 | 12 | 23 | 34.33096 | 18 | 613 | 592.3201 | 34.8916 | 8.58333 | 14.775 |
| *Buthus occitanus* | 0.72 | 41.51 | 45 | 31 | 80 | 299 | 14 | 24 | 33.52219 | 14 | 420 | 692.2346 | 38.58521 | 12 | 14.425 |
| *Buthus occitanus* | -2.65805 | 42.54025 | 42 | 37 | 76 | 507 | 14 | 22 | 23.74131 | 29 | 543 | 571.3872 | 41.28499 | 10.81667 | 12.23333 |
| *Buthus occitanus* | -2.51726 | 42.48847 | 42 | 35 | 74 | 431 | 16 | 23 | 23.76531 | 26 | 472 | 586.0091 | 40.69549 | 10.825 | 12.95417 |
| *Buthus occitanus* | -1.38282 | 42.77203 | 44 | 29 | 73 | 480 | 11 | 27 | 24.70879 | 39 | 813 | 561.9487 | 36.61518 | 9.48333 | 12.45 |
| *Buthus occitanus* | 6.561199 | 43.54034 | 38 | 36 | 77 | 236 | 13 | 26 | 36.61735 | 17 | 804 | 566.9149 | 36.53061 | 8.95 | 13.96667 |
| *Buthus occitanus* | 4.7595 | 44.21258 | 39 | 35 | 68 | 122 | 14 | 26 | 34.38991 | 35 | 840 | 670.1724 | 35.30093 | 10.16667 | 13.70833 |
| *Buthus occitanus* | 4.371048 | 44.53548 | 40 | 34 | 72 | 264 | 14 | 26 | 30.1923 | 43 | 839 | 637.883 | 35.53114 | 9.7 | 12.49167 |

**Table S7.** Continued.

| *Buthus occitanus* | 4.840213 | 44.28937 | 35 | 41 | 77 | 100 | 12 | 24 | 33.82277 | 37 | 854 | 682.0568 | 36.03604 | 10.66667 | 13.68333 |
| --- | --- | --- | --- | --- | --- | --- | --- | --- | --- | --- | --- | --- | --- | --- | --- |
| *Buthus occitanus* | 5.7578 | 43.69792 | 37 | 40 | 72 | 306 | 19 | 23 | 31.0065 | 20 | 695 | 632.4388 | 37.82506 | 10.66667 | 13.36667 |
| *Buthus occitanus* | 4.254613 | 44.19413 | 36 | 42 | 68 | 183 | 17 | 22 | 33.53759 | 33 | 794 | 637.3601 | 35.89976 | 9.90833 | 13.2125 |
| *Buthus occitanus* | 3.158814 | 43.5689 | 38 | 44 | 58 | 389 | 19 | 19 | 28.50706 | 24 | 692 | 578.2871 | 33.68201 | 8.05 | 12.725 |
| *Buthus occitanus* | 2.43246 | 43.13375 | 39 | 36 | 64 | 361 | 16 | 25 | 23.6387 | 29 | 766 | 570.2192 | 34.72803 | 8.3 | 12.5 |
| *Buthus occitanus* | 2.219906 | 42.84241 | 41 | 34 | 58 | 533 | 31 | 25 | 16.62938 | 42 | 801 | 564.8939 | 34.97211 | 8.35833 | 11.5875 |
| *Buthus occitanus* | 2.304855 | 42.56757 | 36 | 41 | 68 | 906 | 16 | 23 | 16.90133 | 49 | 855 | 569.1639 | 37.07827 | 9.15833 | 10.32083 |
| *Buthus occitanus* | 2.580894 | 42.62216 | 38 | 40 | 62 | 590 | 23 | 22 | 20.84066 | 34 | 759 | 564.8309 | 35.35666 | 8.59167 | 11.94583 |
| *Buthus occitanus* | 2.712176 | 42.5458 | 38 | 42 | 62 | 453 | 17 | 21 | 23.22486 | 29 | 708 | 559.1891 | 35.61549 | 8.58333 | 12.83333 |
| *Buthus occitanus* | 3.033312 | 42.50252 | 34 | 47 | 61 | 559 | 22 | 19 | 26.82984 | 25 | 724 | 557.7408 | 32.93304 | 7.54167 | 12.47917 |
| *Buthus occitanus* | 3.066205 | 42.44297 | 35 | 42 | 65 | 330 | 22 | 23 | 28.69963 | 20 | 636 | 555.236 | 34.08441 | 7.94167 | 13.4875 |
| *Buthus occitanus* | 3.186801 | 42.32315 | 28 | 52 | 69 | 112 | 19 | 20 | 33.16392 | 16 | 537 | 559.4674 | 35.68465 | 8.6 | 14.94167 |
| *Buthus occitanus* | 3.202972 | 42.06595 | 32 | 44 | 66 | 100 | 19 | 24 | 34.38679 | 15 | 506 | 551.3336 | 37.24066 | 8.975 | 14.8875 |
| *Buthus occitanus* | 3.140017 | 42.05969 | 33 | 41 | 72 | 200 | 17 | 26 | 31.49759 | 18 | 561 | 561.5176 | 37.66801 | 9.34167 | 14.45417 |
| *Buthus occitanus* | 2.588036 | 42.08745 | 37 | 39 | 62 | 427 | 19 | 23 | 21.50847 | 39 | 768 | 560.2764 | 37.6826 | 9.45833 | 12.8375 |
| *Buthus occitanus* | 2.397528 | 41.98384 | 40 | 39 | 67 | 662 | 19 | 20 | 21.20123 | 44 | 762 | 588.4808 | 36.15435 | 9.29167 | 11.9375 |
| *Buthus occitanus* | 2.165395 | 41.69073 | 39 | 39 | 74 | 473 | 23 | 21 | 23.45438 | 40 | 727 | 574.2558 | 37.51634 | 9.56667 | 13.68333 |
| *Buthus occitanus* | 1.763511 | 41.92846 | 38 | 40 | 73 | 691 | 20 | 22 | 27.5063 | 37 | 738 | 607.3015 | 37.59259 | 10.15 | 12.03333 |
| *Buthus occitanus* | 1.253792 | 41.94598 | 41 | 30 | 73 | 604 | 19 | 29 | 24.77083 | 35 | 643 | 633.0032 | 36.99405 | 10.35833 | 12.39583 |
| *Buthus occitanus* | 0.009154 | 41.50838 | 43 | 37 | 79 | 327 | 14 | 21 | 33.26314 | 13 | 400 | 684.1313 | 39.24731 | 12.16667 | 14.90833 |
| *Buthus occitanus* | -0.28234 | 42.23745 | 37 | 41 | 67 | 741 | 19 | 23 | 24.41441 | 24 | 606 | 654.0011 | 35.62207 | 10.11667 | 13.04167 |
| *Buthus occitanus* | 0.069509 | 42.2981 | 44 | 35 | 71 | 857 | 13 | 21 | 22.51706 | 31 | 680 | 646.9878 | 36.97917 | 10.65 | 12.125 |
| *Buthus occitanus* | 0.111873 | 41.70485 | 45 | 32 | 79 | 231 | 13 | 23 | 30.65869 | 16 | 420 | 695.3301 | 37.83711 | 11.69167 | 15.42083 |
| *Buthus occitanus* | 0.656148 | 42.05131 | 37 | 39 | 71 | 738 | 23 | 24 | 23.33364 | 35 | 676 | 652.939 | 36.39748 | 10.59167 | 12.17917 |
| *Buthus occitanus* | 1.199121 | 42.10546 | 39 | 37 | 69 | 1065 | 17 | 24 | 21.57348 | 48 | 784 | 613.3527 | 37.48474 | 10.23333 | 10.08333 |
| *Buthus occitanus* | 1.409587 | 42.18145 | 37 | 39 | 69 | 1005 | 21 | 23 | 22.40823 | 46 | 780 | 610.7794 | 37.05081 | 9.96667 | 10.11667 |
| *Buthus occitanus* | -0.63606 | 41.37162 | 39 | 38 | 80 | 255 | 13 | 22 | 32.62488 | 16 | 345 | 669.0919 | 40.038 | 12.29167 | 15.42917 |
| *Buthus occitanus* | -1.08145 | 41.57325 | 41 | 35 | 75 | 584 | 18 | 24 | 30.95052 | 22 | 378 | 658.174 | 39.85149 | 12.075 | 13.32083 |
| *Buthus occitanus* | -0.79385 | 42.35344 | 41 | 34 | 75 | 663 | 18 | 25 | 21.69395 | 29 | 601 | 601.6988 | 36.62197 | 9.55833 | 12.7375 |
| *Buthus occitanus* | -0.50899 | 42.33837 | 40 | 40 | 66 | 963 | 15 | 20 | 21.24504 | 31 | 670 | 624.2891 | 36.75758 | 10.10833 | 11.1375 |
| *Buthus occitanus* | -1.62982 | 41.96439 | 40 | 38 | 79 | 408 | 14 | 22 | 26.03625 | 22 | 396 | 619.778 | 39.75939 | 11.29167 | 13.72917 |

**Table S8.** Bioclimatic variables used in ecological niche modeling valid species of *Buthus* Leach, 1815 scorpions in the Iberian Peninsula and Southeastern France.

| **Code** | **Bioclimatic variable** |
| --- | --- |
| BIO1 | annual mean temperature |
| BIO2 | mean diurnal range (mean monthly maximum tem­perature -minimum temperature) |
| BIO3 | isothermality (bio2/bio7) (×100) |
| BIO4 | temperature seasonality (standard devi­ation × 100) |
| BIO5 | maximum temperature of warmest month |
| BIO6 | min temperature of coldest month |
| BIO7 | temperature annual range (bio5 - bio6) |
| BIO8 | mean temperature of wettest quarter |
| BIO9 | mean temperature of driest quarter |
| BIO10 | mean temperature of warmest quarter |
| BIO11 | mean temperature of coldest quarter |
| BIO12 | annual precipitation |
| BIO13 | precipitation of wettest month |
| BIO14 | precipitation of driest month |
| BIO15 | precipitation seasonality (coefficient of variation) |
| BIO16 | precipitation of wettest quarter |
| BIO17 | precipitation of driest quarter |
| BIO18 | precipitation of warmest quarter |
| BIO19 | precipitation of coldest quarter |

**Table S9.** Performance of models for valid species of *Buthus* Leach, 1815 scorpions in the Iberian Peninsula and Southeastern France, indicating number of non-zero coefficients (parameters), feature class (hinge, linear, quadratic), omission rate for 10^th^ percentile training values, Akaike Information Criterion (AICc) for small sample sizes, and Area Under the Curve (AUC) metric.

| **Species** | **Parameters** | **Feature** | **AICc** | **Omission (10%)** | **AUC** |
| --- | --- | --- | --- | --- | --- |
| *Buthus delafuentei* | 9 | LQ | 52.79 | 0.0 | 0.998 |
| *Buthus elongatus* | 15 | HLQ | 62.57 | 0.074 | 0.999 |
| *Buthus garcialorcai* | 7 | LQ | 24.21 | 0.071 | 0.998 |
| *Buthus halius* | 35 | HLQ | 146.82 | 0.09 | 0.971 |
| *Buthus iaspis* | 20 | HLQ | 149.51 | 0.071 | 0.999 |
| *Buthus ibericus* | 17 | HLQ | 90.63 | 0.037 | 0.999 |
| *Buthus manchego* | 16 | HLQ | 296.89 | 0.053 | 0.993 |
| *Buthus montanus* | 13 | HLQ | 198.81 | 0.0 | 0.998 |
| *Buthus occitanus* | 61 | HLQ | 3898.89 | 0.094 | 0.983 |

**Table S10.** Bioclimatic and soil variables with highest permutation importance for valid species of *Buthus* Leach, 1815 scorpions in the Iberian Peninsula and Southeastern France following Goodman et al. (2022): optimal MaxEnt model, with % of permutation importance and contribution of each environmental variable, and response curve behavior expressed with that variable in relation to suitability.

| **Species** | **Environmental variable** | **Permutation (%)** | **Contribution (%)** | **Response** |
| --- | --- | --- | --- | --- |
| *Buthus delafuentei* | annual mean temperature (bio1) | 75.2 | 63.2 | positive threshold |
|  | precipitation seasonality (bio15) | 18 | 25.4 | positive threshold |
|  | sand | 2.9 | 9.1 | positive sigmoidal |
|  | isothermality (bio3) | 2.1 | 0.1 | negative hinge |
|  | annual precipitation (bio12) | 1.4 | 1.2 | negative threshold |
| *Buthus elongatus* | precipitation seasonality (bio15) | 95.8 | 79.4 | positive threshold |
|  | elevation (m) | 2.4 | 14.1 | positive threshold |
|  | annual precipitation (bio12) | 1.6 | 0.7 | positive quadratic |
|  | pH | 0.1 | 1.7 | positive quadratic |
|  | temperature seasonality (bio4) | 0.1 | 0.4 | positive sigmoidal |
| *Buthus garcialorcai* | precipitation of driest month (bio14) | 57 | 20.5 | negative threshold |
|  | elevation (m) | 20.4 | 18.1 | positive sigmoidal |
|  | mean diurnal range (bio2) | 12.1 | 27.5 | positive sigmoidal |
|  | coarse | 8.1 | 15.2 | positive sigmoidal |
|  | silt | 2.3 | 0.1 | positive hinge |
| *Buthus halius* | precipitation seasonality (bio15) | 29.6 | 68.7 | positive hinge |
|  | pH | 17.5 | 9.3 | positive quadratic |
|  | annual precipitation (bio12) | 14.3 | 1.7 | negative hinge |
|  | elevation (m) | 10 | 3.6 | positive quadratic |
|  | coarse | 9.7 | 11.5 | positive hinge |
| *Buthus iaspis* | isothermality (bio3) | 68.3 | 1.1 | positive threshold |
|  | annual precipitation (bio12) | 17.8 | 17.6 | negative threshold |
|  | precipitation of driest month (bio14) | 11.9 | 12.1 | negative threshold |
|  | elevation (m) | 1.3 | 1.6 | negative hinge |
|  | annual mean temperature (bio1) | 0.6 | 1 | positive hinge |

**Table S10.** Continued.

| **Species** | **Environmental variable** | **Permutation (%)** | **Contribution (%)** | **Response** |
| --- | --- | --- | --- | --- |
| *Buthus ibericus* | annual precipitation (bio12) | 50 | 1.2 | positive threshold |
|  | precipitation of driest month (bio14) | 47.8 | 6.8 | positive hinge |
|  | pH | 1.4 | 0.6 | positive sigmoidal |
|  | isothermality (bio3) | 0.5 | 1.6 | negative hinge |
|  | precipitation seasonality (bio15) | 0.2 | 73 | positive quadratic |
| *Buthus manchego* | pH | 63.5 | 52.8 | positive threshold |
|  | annual mean temperature (bio1) | 12.3 | 3.8 | positive threshold |
|  | elevation (m) | 12.2 | 27.6 | positive quadratic |
|  | precipitation of driest month (bio14) | 7.7 | 2.5 | positive quadratic |
|  | mean diurnal range (bio2) | 2 | 0.5 | positive hinge |
| *Buthus montanus* | elevation (m) | 53.5 | 51 | positive threshold |
|  | precipitation of driest month (bio14) | 44.8 | 9.6 | negative threshold |
|  | precipitation seasonality (bio15) | 1.5 | 33.6 | positive quadratic |
|  | temperature seasonality (bio4) | 0.1 | 4.7 | positive hinge |
|  | coarse | 0.1 | 0.1 | positive sigmoidal |
| *Buthus occitanus* | precipitation of driest month (bio14) | 36.5 | 50.3 | positive hinge |
|  | annual mean temperature (bio1) | 26.5 | 6.8 | positive sigmoidal |
|  | coarse | 13.4 | 5.6 | positive threshold |
|  | isothermality (bio3) | 7.3 | 4 | negative threshold |
|  | clay | 3.7 | 18.6 | positive quadratic |

**Table S11.** Statistics of Canonical Correlation Analysis (CCA) and environmental variables used in ecological niche models of valid species of *Buthus* Leach, 1815 scorpions in the Iberian Peninsula and Southeastern France.

| **Explanatory variables** | **Axis 1** | **Axis 2** | **χ^2^** | ***F*** | ***p*-value** |
| --- | --- | --- | --- | --- | --- |
| bio1 | -0.24 | 0.241 | 0.046 | 2.381 | 0.018 |
| bio2 | -0.013 | -0.782 | 0.072 | 3.713 | 0.001 |
| bio3 | -0.291 | -0.237 | 0.108 | 5.583 | 0.001 |
| bio4 | 0.265 | -0.684 | 0.095 | 4.907 | 0.001 |
| bio12 | -0.136 | 0.459 | 0.131 | 6.775 | 0.001 |
| bio14 | 0.8 | 0.207 | 0.103 | 5.298 | 0.001 |
| bio15 | -0.941 | 0.228 | 0.61 | 31.319 | 0.001 |
| clay | 0.072 | -0.123 | 0.041 | 2.151 | 0.02 |
| coarse | -0.134 | -0.162 | 0.131 | 6.752 | 0.001 |
| elevation | -0.182 | -0.534 | 0.479 | 24.59 | 0.001 |
| pH | 0.249 | -0.439 | 0.342 | 17.579 | 0.001 |
| sand | -0.276 | 0.067 | 0.313 | 16.088 | 0.001 |
| silt | 0.378 | 0.001 | 0.4 | 20.545 | 0.001 |

**Supplementary Figures**


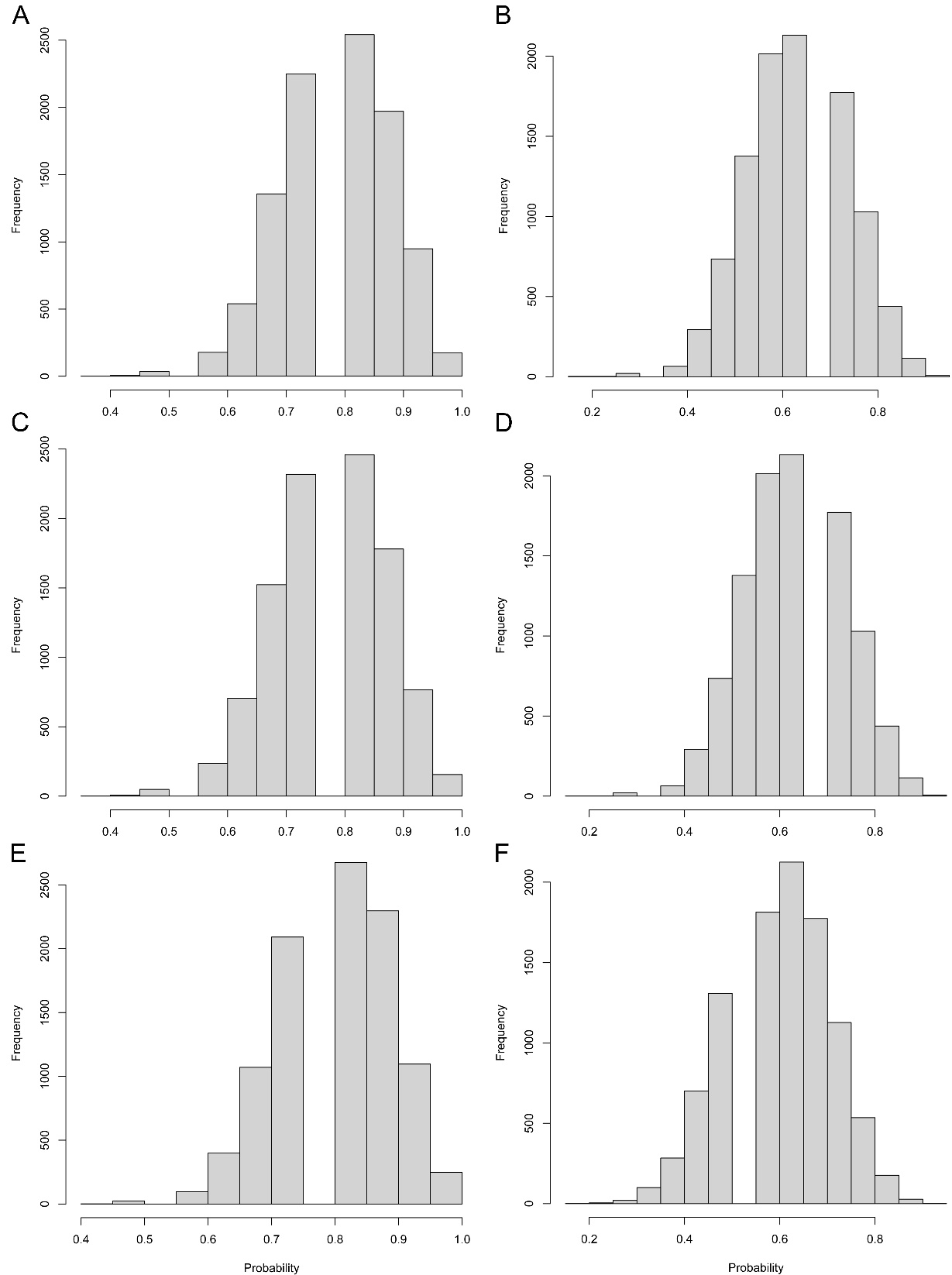


**Figure S1.** Histograms showing accuracy across 10,000 cross-validated Linear Discriminant Analysis (LDA) models based on morphometric data from *Buthus* Leach, 1815 scorpions in the Iberian Peninsula and Southeastern France. X-axis represents probability that random male (**A, C, E**) or female (**B, D, F**) specimens were correctly assigned to grouping factor, whereas y-axis shows frequency of model probabilities. Grouping factors establsihed based on nuDNA lineages (**A, B**), mtDNA lineages (**C, D**), and valid species identified through simultaneous analysis of morphology and DNA (**E, F**).


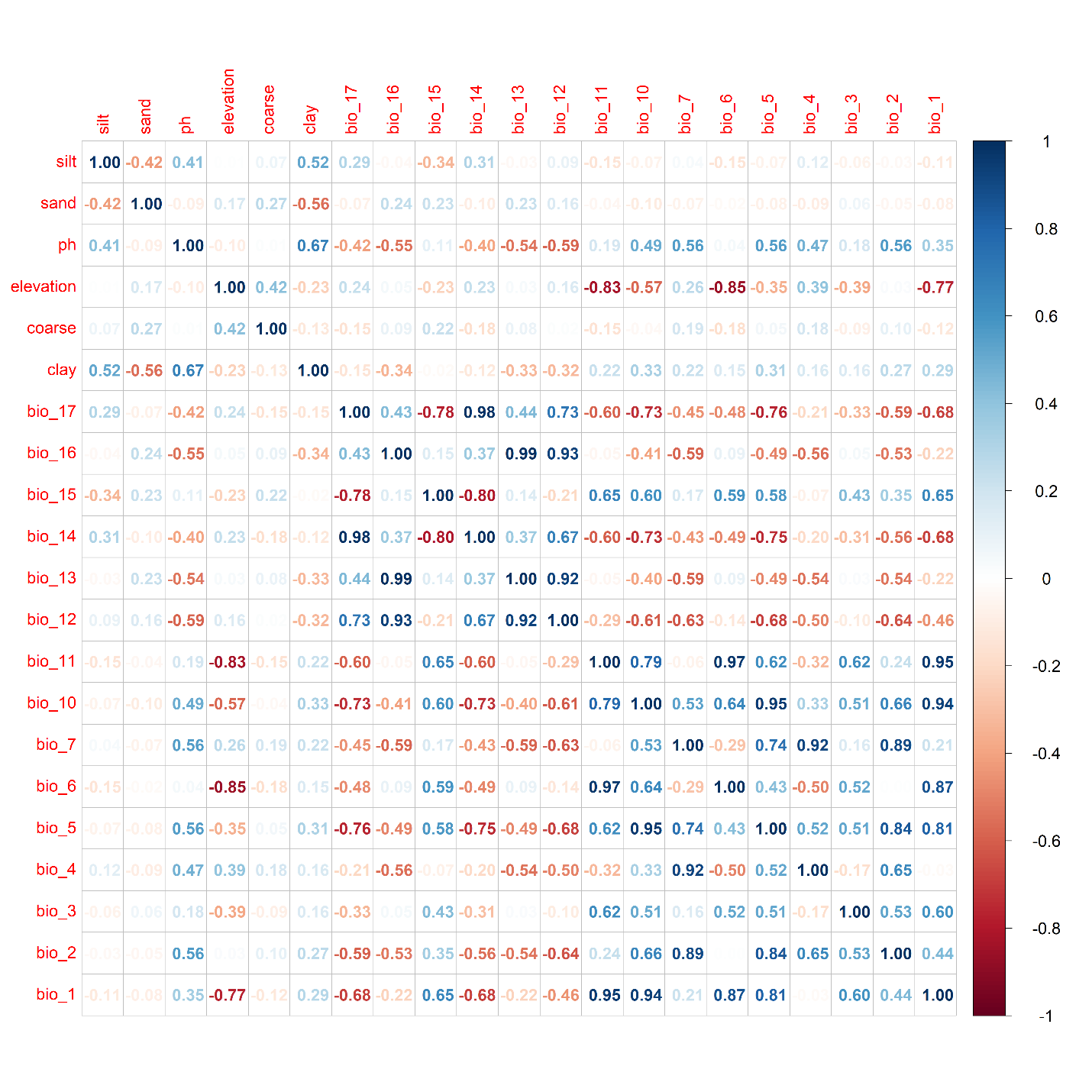


**Figure S2.** Correlation indices between environmental variables used in ecological niche models of valid species of *Buthus* Leach, 1815 scorpions in the Iberian Peninsula and Southeastern France.


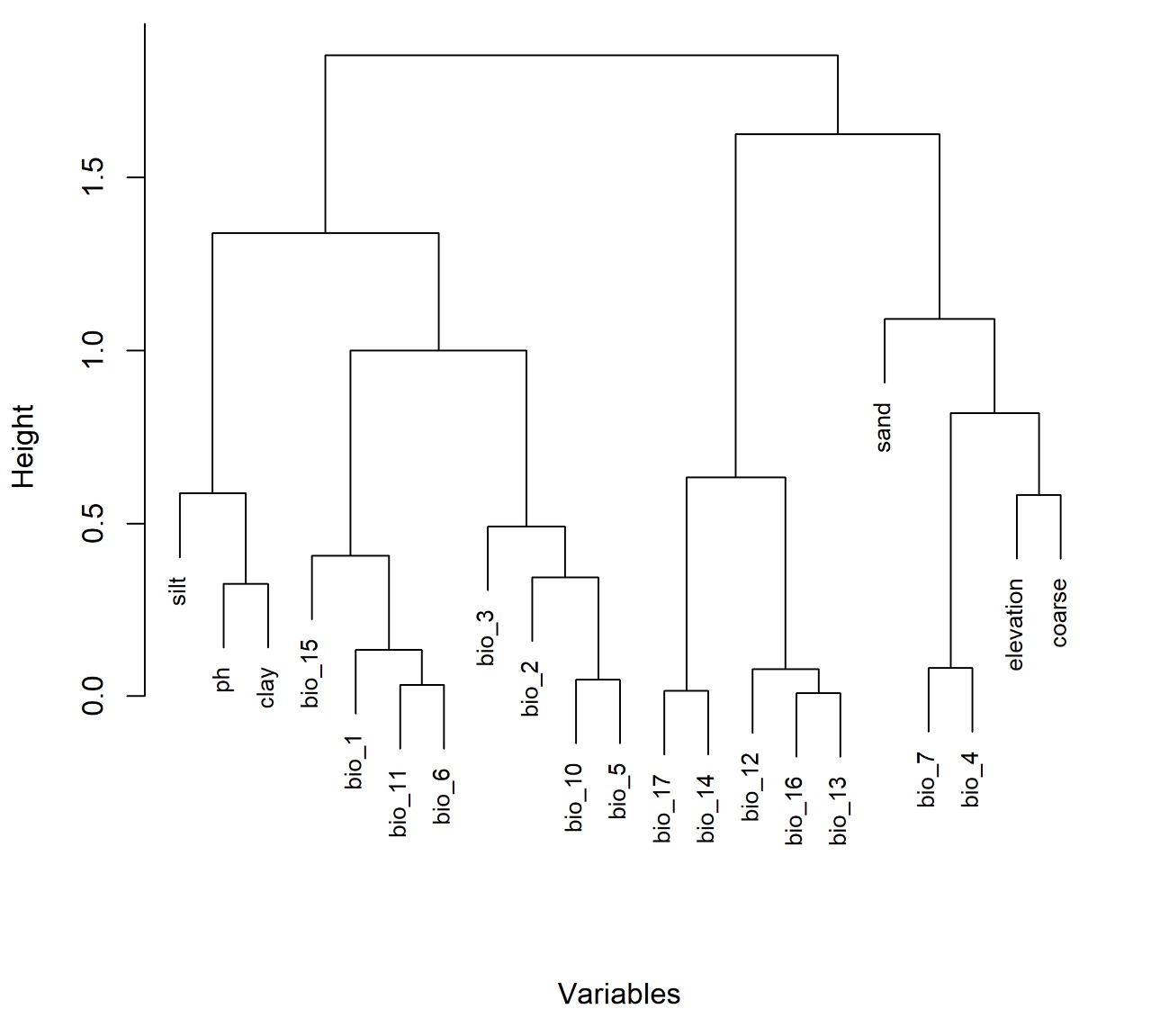


**Figure S3.** Dendrogram from hierarchical cluster analyses of matrix of correlation indices among environmental variables used in ecological niche models of valid species of *Buthus* Leach, 1815 scorpions in the Iberian Peninsula and Southeastern France (Figure S2). Clustered variable pairs/trios with a correlation >80% considered collinear and removed from subsequent analyses (i.e., ecological niche modeling and Canonical Correlation analysis).

**Supplementary References**

[1] G. Giribet, S. Carranza, J. Baguna, M. Riutort, C. Ribera, First molecular evidence for the existence of a Tardigrada + Arthropoda clade, Mol. Biol. Evol. 13 (1996) 76–84. https://doi.org/10.1093/oxfordjournals.molbev.a025573.

[2] W.C. Wheeler, R. Bang, R.T. Schuh, Cladistic relationships among higher groups of Heteroptera: congruence between morphological and molecular data sets, Insect Syst. Evol. 24 (1993) 121–137. https://doi.org/10.1163/187631293X00235.

[3] G.B. Nunn, B.F. Theisen, B. Christensen, P. Arctander, Simplicity-correlated size growth of the nuclear 28S ribosomal RNA D3 expansion segment in the crustacean order isopoda, J. Mol. Evol. 42 (1996) 211–223. https://doi.org/10.1007/BF02198847.

[4] L. Prendini, W.C. Wheeler, Scorpion higher phylogeny and classification, taxonomic anarchy, and standards for peer review in online publishing, Cladistics 21 (2005) 446–494. https://doi.org/10.1111/j.1096-0031.2005.00073.x.

[5] Y. Ji, D. Zhang, L. He, Evolutionary conservation and versatility of a new set of primers for amplifying the ribosomal internal transcribed spacer regions in insects and other invertebrates, Mol. Ecol. Notes 3 (2003) 581–585. https://doi.org/10.1046/j.1471-8286.2003.00519.x.

[6] T.D. Kocher, W.K. Thomas, A. Meyer, S. V Edwards, S. Pääbo, F.X. Villablanca, A.C. Wilson, Dynamics of mitochondrial DNA evolution in animals: amplification and sequencing with conserved primers., Proc. Natl. Acad. Sci. 86 (1989) 6196–6200. https://doi.org/10.1073/pnas.86.16.6196.

[7] B. Gantenbein, V. Fet, C.R. Largiadèr, A. Scholl, First DNA phylogeny of Euscorpius and its bearing on taxonomy and biogeography of this genus, Biogeographica 1876 (1999).

[8] C. Simon, A. Francke, A. Martin, The polymerase chain reaction: DNA extraction and amplification., in: G. Witt, A. Johnson, J. Young (Eds.), Mol. Tech. Taxon., New York: Springer Verlag, 1991: pp. 329–355.

[9] O. Folmer, M. Black, W. Hoeh, R. Lutz, R. Vrijenhoek, DNA primers for amplification of mitochondrial cytochrome c oxidase subunit I from diverse metazoan invertebrates., Mol. Mar. Biol. Biotechnol. 3 (1994) 294–299. https://doi.org/10.1071/ZO9660275.

[10] R.W. Bryson, B.R. Riddle, M.R. Graham, B.T. Smith, L. Prendini, As old as the hills: Montane Scorpions in southwestern North America reveal ancient associations between biotic diversification and landscape history, PLoS One 8 (2013) e52822. https://doi.org/10.1371/journal.pone.0052822.

[11] L. Prendini, T.M. Crowe, W.C. Wheeler, Systematics and biogeography of the family Scorpionidae (Chelicerata : Scorpiones), with a discussion on phylogenetic methods, Invertebr. Syst. 17 (2003) 185. https://doi.org/10.1071/IS02016.

[12] N.A. Valdez-Cruz, S. Dávila, A. Licea, M. Corona, F.Z. Zamudio, J. García-Valdes, L. Boyer, L.D. Possani, Biochemical, genetic and physiological characterization of venom components from two species of scorpions: Centruroides exilicauda Wood and Centruroides sculpturatus Ewing, Biochimie 86 (2004) 387–396. https://doi.org/10.1016/j.biochi.2004.05.005.

[13] Harrison R. G., D.M. Rand, W.C. Wheeler, Mitochondrial DNA variation in field crickets across a narrow hybrid zone, Mol. Biol. Evol. 4 (1987) 144–158. https://doi.org/10.1093/oxfordjournals.molbev.a040436.
